# Diversity-oriented synthesis of 17-spirosteroids

**DOI:** 10.3762/bjoc.16.79

**Published:** 2020-04-28

**Authors:** Benjamin Laroche, Thomas Bouvarel, Martin Louis-Sylvestre, Bastien Nay

**Affiliations:** 1 Unité Molécules de Communication et Adaptations des Micro-organismes (MCAM), Muséum National d'Histoire Naturelle, CNRS, Paris, France; 2 Laboratoire de Synthèse Organique, Ecole Polytechnique, CNRS, ENSTA, Institut Polytechnique de Paris, Palaiseau Cedex, France

**Keywords:** diversity-oriented strategy, 17-ethynyl-17-hydroxysteroids, ring-closing enyne metathesis, spirosteroids, steroids

## Abstract

A diversity-oriented synthesis (DOS) approach has been used to functionalize 17-ethynyl-17-hydroxysteroids through a one-pot procedure involving a ring-closing enyne metathesis (RCEYM) and a Diels–Alder reaction on the resulting diene, under microwave irradiations. Taking advantage of the propargyl alcohol moiety present on commercially available steroids, this classical strategy was applied to mestranol and lynestrenol, giving a collection of new complex 17-spirosteroids.

## Introduction

Diversity-oriented synthesis (DOS) is a powerful approach to access collections of structurally diverse compounds in a few synthetic steps [1–7]. It can be more relevant when the chemical diversity is centred on biologically validated scaffolds [8–10], referred to as privileged structures in medicinal chemistry [11–14]. Spanning unexplored chemical space, DOS strategies have been successfully applied to the generation of biologically active libraries for screening, leading to the discovery of medicinally relevant compounds [15–17].

**Scheme 1:**
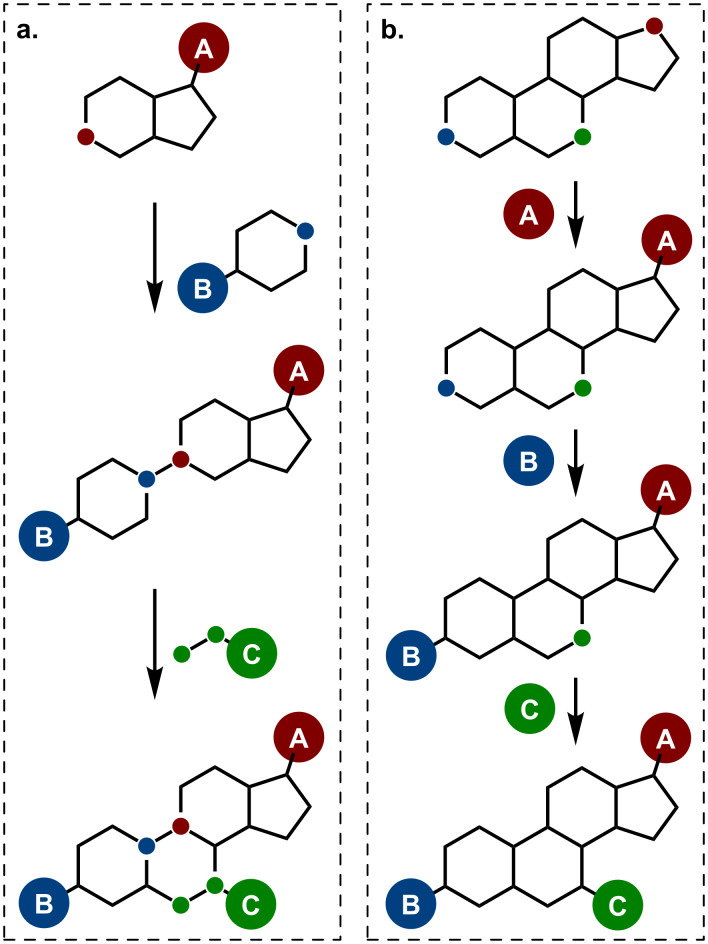
Two diversity-oriented strategies: (a) Diversity installed during the construction of the skeleton; (b) Diversity installed lately by functionalization.

Once a biologically relevant scaffold has been identified, the diversity-generating workflow can basically follow two complementary approaches, either by de novo synthesis sequentially incorporating the diversity [15,18–19], or by late functionalization of easily accessible biologically relevant materials. This second strategy can take benefit of existing functional groups on the substrate, or rely on direct C–H functionalization [20–33]. Taking steroids as an example of highly targeted privileged structures, numerous diversity-oriented approaches were developed, generating diversity either at the construction stage (Scheme 1a) [34–35], or by transformation of the main steroidal core (Scheme 1b) [36–39].

**Figure 1:**
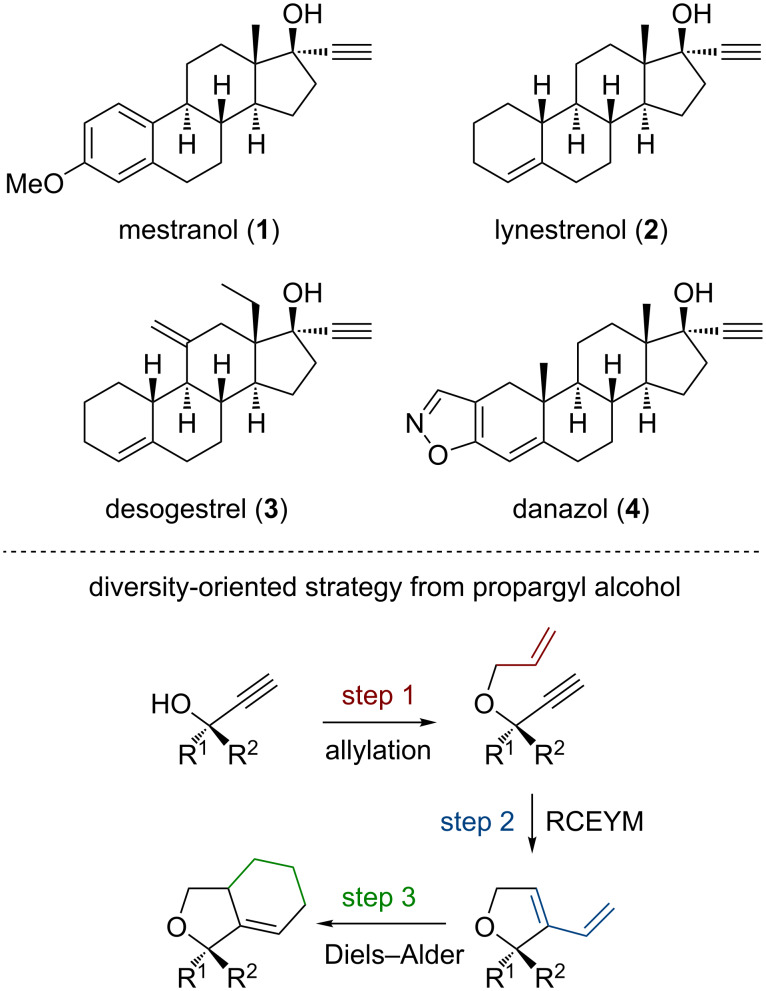
Structure of four 17-ethynyl-17-hydroxysteroids **1**–**4** (top) and how they could be used to generate diversity (down).

An important class of steroid medicines, also bearing a crucial propargylic alcohol function for our targeted strategy, lies on the 17-ethynyl-17-hydroxysteroids (Figure 1). They are commonly used as contraceptive while their skeleton is structurally diversified, especially at ring A. They are thus synthetically relevant as starting materials in a DOS approach. Indeed, after installing an alkenyl ether on the propargylic alcohol, the resulting enyne is a good substrate for ring closing enyne metathesis (RCEYM) towards new diene substrates [40–42], which can be employed in Diels–Alder reactions with a variety of dienophiles (Figure 1) [43–54]. This strategy has been previously used to generate medicinally relevant diversity in other compound series [49,55–58]. However, it has surprisingly never been applied to 17-ethynyl-17-hydroxysteroids.

Most steroids bind cytosolic receptors that are then taken up into the nucleus to modulate gene transcription, or directly bind nuclear receptors [59–61]. In principle, steroids could thus be used as cargo molecules to deliver new chemical entities inside the nucleus, highlighting the potential of hybrid steroid molecules [62]. For example, De Riccardis designed steroid scaffolds directly fused to a cytotoxic anthraquinone moiety and obtained positive, yet limited, cytotoxic activities [63–64]. Furthermore, a steroidal anti-estrogen–doxorubicin conjugate was synthesized by Hanson, showing a 70-fold increase of activity compared to doxorubicin in inhibiting cell proliferation and promoting cell death [65]. Today, the research on steroids is still extremely active thanks to their huge medicinal potential [66–67].

In this context and owing to the commercial availability of 17-ethynyl-17-hydroxysteroids, we decided to apply a DOS approach to the synthesis of steroid analogues based on mestranol (**1**) and lynestrenol (**2**). By using the sequence of RCEYM/Diels–Alder reaction, we rapidly synthesized "17-spirosteroid" compounds, orthogonally linking a non-steroidal polycyclic moiety to the steroid part at position 17. Spirocyclic systems play an important role in current medicinal chemistry thanks to their characteristic 3D geometry [68]. In particular, with 17-spirosteroids [69–76], the three dimensional complexity of the steroid CD-ring system is expected to be substantially affected.

## Results and Discussion

**Scheme 2:**
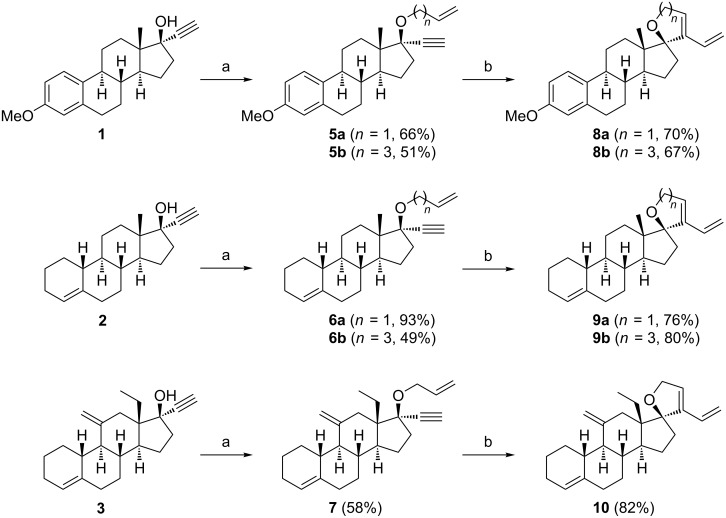
Synthesis of spirocyclic steroid derivatives by the RCEYM reaction. Conditions: (a) NaH (2 equiv), allyl or 4-pentenyl bromide (2 equiv), DMF, 0 °C→rt; (b) Second-generation Grubbs catalyst (**G-II)**, toluene, under microwave at 120 °C (**5a**, **6a**, **7**) or 170 °C (**5b**, **6b**).

The first goal of this work aimed at synthesizing spirocyclic derivatives of several steroidal skeletons, starting from available 17-ethynyl-17-hydroxysteroid compounds **1**–**3** (Scheme 2). Alkylation of the alcohol function proceeded well under classical conditions (NaH then RCH_2_Br in DMF), either in the presence of allyl bromide or of 4-pentenyl bromide. Mestranol (**1**), lynestrenol (**2**) and desogestrel (**3**) gave alkylated products **5**–**7** in good yields and were readily engaged in the RCEYM reaction.

**Figure 2:**
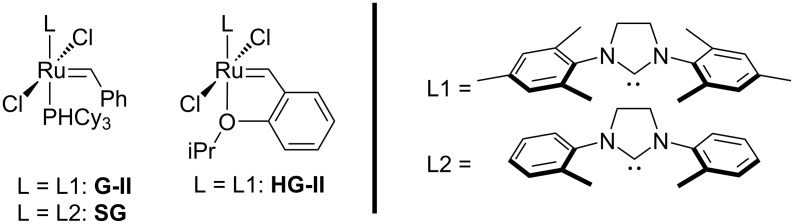
Structure of metathesis catalysts.

The RCEYM reaction was then undertaken post alkylation. In fact, these quaternary propargyl ethers are particularly hindered. For such hindered substrates, we previously employed the Stewart–Grubbs catalyst combined with microwave heating at 120 °C (allyl ethers) or 170 °C (4-pentenyl ethers), demonstrating improved yields [77]. These temperatures were needed for the reaction to quickly go to completion. With 17-*O*-allylmestranol (**5a**), a rapid screening of the Stewart–Grubbs (**SG**), second-generation Grubbs (**G-II**) and second generation Hoveyda–Grubbs catalysts (**HG-II**) (Figure 2) showed that all catalysts are efficient at performing the RCEYM transformation into **8a**, with the best performance by **SG** (93%). However, the less costly catalyst **G-II** worked well and was retained for the next study (Scheme 2). Thus, in the presence of **G-II** in toluene and under microwave heating, the reaction provided 17-spirosteroid dienes **8**–**10** in good yields (67–82%).

**Scheme 3:**
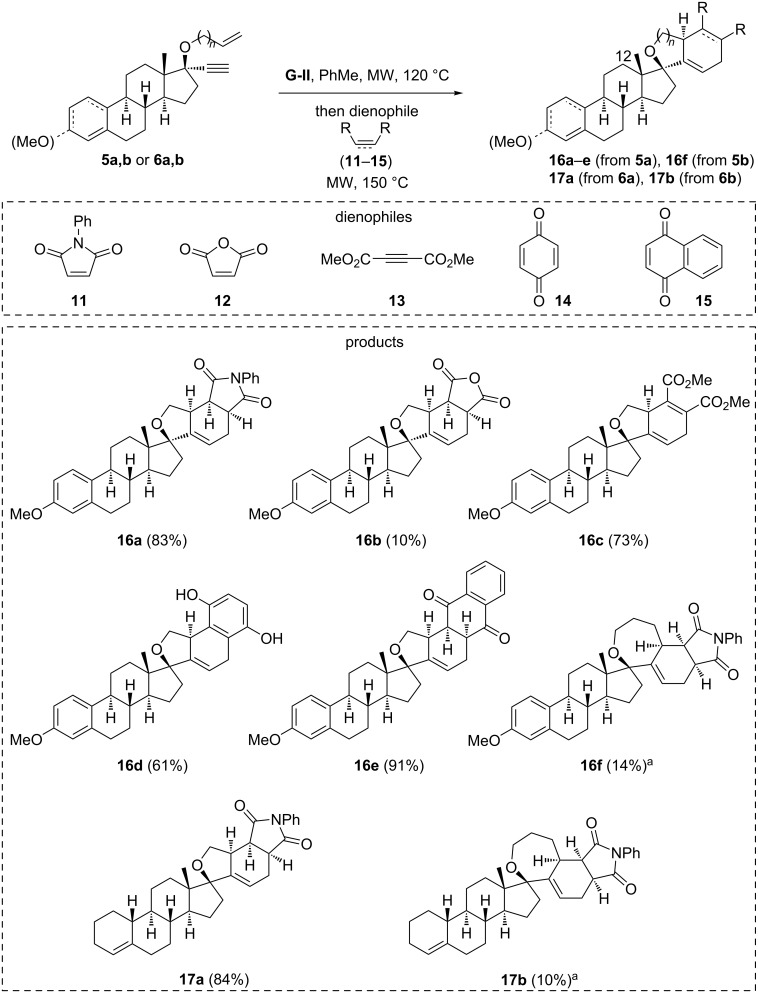
One-pot RCEYM/Diels–Alder reaction strategy applied to the mestranol and lynestrenol cores. Notes: ^a^Isolated among other unseparable stereoisomers (overall cycloadduct yields: 65% and 50%, for the respective reactions).

Spirocyclic vinylcyclopentene substrates **8a**,**b** and **9a**,**b** were engaged in Diels–Alder reactions with a variety of dienophiles (**11**–**15**). The reaction proved efficient when adding the dienophile and heating in the same pot just after the RCEYM (Scheme 3). From mestranol derivative **5a**, *endo* cycloadducts **16a**–**e** were obtained in good to excellent yields. However, the reaction with maleic anhydride (**12**) gave a poor yield attributed to the reactivity of the obtained succinic anhydride derivative (**16b**) upon purification. Other unsymmetrical dienophiles (methyl acrylate, methyl propiolate) gave complex mixtures of regio- and/or stereoisomers. The reaction of lynestrenol enyne **6a** with *N*-phenylmaleimide was also performed to furnish compound **17a** in 84% yield.

Concerning the use of oxepane-type dienes **8b** and **9b**, none of the attempted Diels–Alder reactions proved efficient, systematically leading to a mixture of diastereoisomers and difficult purifications. Only cycloadducts **16f** and **17b** could be isolated in minute amounts (Scheme 3).

**Figure 3:**
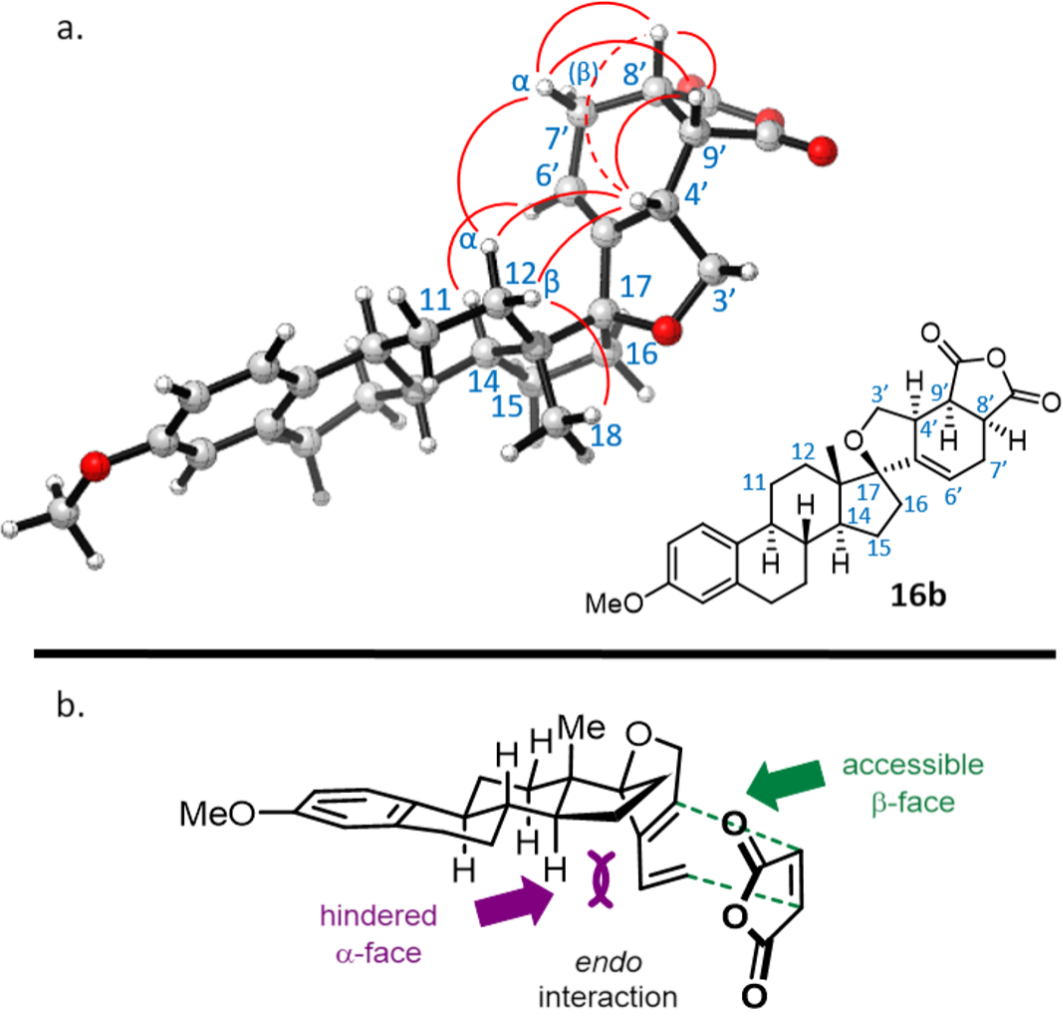
Rationale for the observed stereochemistry: (a) NOESY correlations observed on compound **16b**, showing key correlations between protons of the cyclohexene α-face and both protons H-12 (the dotted line indicates a weak correlation); (b) Model to explain the diene–dienophile interaction during the Diels–Alder reaction.

Finally, the structure and stereochemistry of the products were determined by 2D NMR analysis. Taking **16b** as an example, the key NOESY correlations between proton H-4' at 2.79 ppm and steroid protons H-12 at 1.33/1.65 ppm demonstrated that H-4' is on the α-face of the cyclohexene (Figure 3a). The *endo* character of the Diels–Alder cycloadducts was demonstrated thanks to NOESY correlations observed between protons H-4', H-7'α at 2.30 ppm, H-8' at 3.46 ppm, and H-9' at 3.54 ppm, all on the same cyclohexene α-face. Overall, the facial selectivity of this Diels–Alder reaction seems controlled by the steric hindrance imposed by the steroid skeleton on the dienyl tetrahydrofuran α-face, imposing orbital interactions with the dienophile from the β-face (Figure 3b). All these structures have an extended skeleton, with the steroid part being perpendicular to the cycloadduct moiety thanks to the spiro junction.

## Conclusion

A one-pot RCEYM/Diels–Alder reaction sequence was used to generate a new type of structural diversity in steroids, taking benefit of the propargylic alcohol group of 17-ethynyl-17-hydroxysteroids. After an alkenylation of the alcohol present on the commercially available steroids, providing with enyne substrates, the one-pot reaction manifold enabled the anchorage of non-steroidal spirocyclic moieties at C-17. The procedure proved efficient and stereoselective with mestranol and lynestrenol derivatives incorporating a 2-tetrahydrofuranyl spirocycle at C-17. It was more difficult with 2-oxepanyl structures due to the poor diastereoselectivity of the Diels–Alder reaction in this case. Finally, we expect to apply this strategy to complementary reactions like the Pauson–Khand reaction that may be useful to cyclize enyne substrates like **5**–**7** and expand the spirosteroid diversity. All compounds will be engaged in biological screening campaign in the near future.

## Experimental

### General procedure A for alkylation of 17-ethynyl-17-hydroxysteroids

To a suspension of 17-ethynyl-17-hydroxysteroids (1 mmol) in dry DMF (10 mL), NaH (2 mmol, used as a 60% stabilized mixture in oil) was added at 0 °C. After stirring for 1.5 hours, the alkyl bromide (2 mmol) was added dropwise at 0 °C and the resulting mixture was stirred at room temperature for 16 hours. The reaction was quenched by the addition of water (10 mL). The solution was extracted with diethyl ether (3 × 20 mL), the combined organic layers were then washed with water (20 mL), brine (20 mL), and dried over MgSO_4_, filtered, and concentrated under reduced pressure. The crude material was purified by chromatography on silica gel, using a 98:2 petroleum ether/EtOAc eluent, to afford the alkylated compound.

### General procedure B for ring closing enyne metathesis

The enyne substrate (1 mmol) was dissolved in dry toluene (*c* 0.03 M). The solution was placed in a sealed microwave tube containing a magnetic stirrer under argon. The Grubbs second generation catalyst (2 mol %) was added and the reaction mixture was stirred at 120 °C for 17-allyloxysteroids, or at 170 °C for 17-(4-penten-1-yloxy)steroids, for 1 h under microwave irradiation. The solvent was removed by evaporation, and the resulting crude material was purified by chromatography on silica gel (petroleum ether/EtOAc 99:1) to afford the spirocyclic diene.

### General procedure C for one-pot RCEYM/Diels–Alder reaction

The enyne (1 mmol) was dissolved in dry toluene (*c* 0.03 M). The solution was placed in a sealed microwave tube containing a magnetic stirrer under argon. The Grubbs second generation catalyst (2 mol %) was added and the reaction mixture was stirred at 120 °C for 1 h under microwave irradiation. The microwave tube was opened and the dienophile (1.2 mmol) was added in one portion. The mixture was stirred at 150 °C for 35 min. The solvent was removed and the crude material was purified by chromatography on silica gel (petroleum ether/EtOAc 8:3) to afford the product.

## Supporting Information

File 1 General experimental details, compound descriptions, ^1^H and ^13^C NMR spectra.
