# Supplementary material for: Diversity-oriented synthesis of 17-spirosteroids
Source: Beilstein J Org Chem. 2020 Apr 28;16:880–7. doi: 10.3762/bjoc.16.79 (PMC7214869; doi:10.3762/bjoc.16.79)

BEILSTEIN JOURNAL OF ORGANIC CHEMISTRY

Supporting Information

for

Diversity-oriented synthesis of 17-spirosteroids

Benjamin Laroche, Thomas Bouvarel, Martin Louis-Sylvestre and Bastien Nay

Beilstein J. Org. Chem. **2020,** *16,* 880–887. doi:10.3762/bjoc.16.79

General experimental details, compound descriptions, ^{1}H and ^{13}C NMR spectra

License and Terms: This is a supporting information file under the terms of the Creative Commons Attribution License (http://creativecommons.org/licenses/by/4.0). Please note that the reuse, redistribution and reproduction in particular requires that the authors and source are credited.
The license is subject to the *Beilstein Journal of Organic Chemistry* terms and conditions: (https://www.beilstein-journals.org/bjoc)

Content

General experimental methods S1

General procedures S1

Compound description S2

Copies of NMR spectra S11

General experimental methods

All reactions were carried out in oven-dried vessels under an atmosphere of argon in anhydrous solvents. Dry toluene was distilled in the presence of the sodium/benzophenone couple before use. Dry DMF was purchased (Aldrich). Reactions under microwave conditions were carried out in an Anton Paar Monowave 300 apparatus (850) equipped with an infrared external temperature sensor for temperature monitoring and a stirrer. Sealed microwave tubes were utilized for microwave experiments. Reactions were monitored by TLC on Merck silica gel 60 F254 aluminum sheets, using UV absorption then vanillin-H_2SO_4 (1% vanillin in ethanol + 2% H_2SO_4) or basic permanganate (1% $KMnSO_4$ + 15% Na_2CO_3 in water). The products were purified by flash silica gel column chromatography (Geduran silica gel Si 60, 40-63 µm). NMR spectra were recorded on Bruker 400 or 600 MHz Avance III spectrometers. Chemical shifts (δ) are quoted in ppm with internal calibration from the residual solvent peak ($CHCl_3$: 7.27, 77.0 ppm for ^1H and ^{13}C NMR, respectively). The following abbreviations are used to designate multiplicities: *s* = singlet, *d* = doublet, *t* = triplet, *q* = quadruplet, *m* = multiplet, *quint* = quintet, *br* = broad. High resolution mass spectra (HRMS) of synthesized compounds were measured on a Qq-ToF spectrometer using electrospray ionization (ESI). Infrared spectra were recorded on a Shimadzu 8400S FTIR spectrometer. Specific rotations were recorded on a Perkin Elmer 341 polarimeter at 20 °C. Melting points were measured on a Büchi B-545 apparatus.

General procedures

See the experimental part of the article.

Compound description

17-*O*-Allylmestranol (5a)

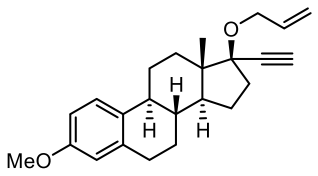

White powder (66% from **1** following procedure A).

M.p. = 67-69 °C.

[α]$_D$^{20} = +10 ($CHCl_3$, *c* 0.1).

^{1}H NMR (600 MHz, $CDCl_3$) δ ppm: 0.91 (*s*, 3H), 1.31-1.54 (*m*, 4H), 1.72-1.90 (*m*, 4H), 1.97-2.07 (*m*, 2H), 2.18-2.37 (*m*, 3H), 2.61 (*s*, 1H), 2.79-2.91 (*m*, 2H), 3.77 (*s*, 3H), 4.16 (*m*, 2H), 5.12 (*m*, 1H), 5.29 (*m*, 1H), 5.89-5.98 (*m*, 1H), 6.64 (*d*, J = 2.8 Hz, 1H), 6.72 (*dd*, J = 2.8, 8.6 Hz, 1H), 7.22 (*d*, J = 8.6 Hz, 1H).

^{13}C NMR (150 MHz, $CDCl_3$) δ ppm: 12.8, 22.8, 26.5, 27.2, 29.8, 34.1, 37.2, 39.2, 43.5, 47.7, 49.5, 55.2, 66.7, 75.7, 85.0, 85.2, 111.5, 113.8, 115.7, 126.4, 132.6, 135.4, 137.9, 157.4.

HRMS (ESI+) *m/z*: calculated for $C_{24}H_{31}O_2^+$ [MH^+]: 351.2319; found: 351.2321.

17-*O*-(4-Penten-1-yl)mestranol (5b)

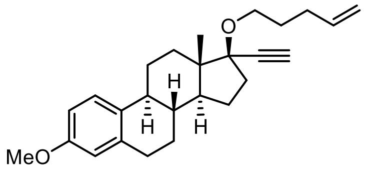

White powder (51% from **1** following procedure A).

M.p. = 52 °C.

[α]$_D$^{20} = +10 ($CHCl_3$, *c* 0.1).

^{1}H NMR (600 MHz, $CDCl_3$) δ ppm: 0.88 (*s*, 3H), 1.33-1.53 (*m*, 4H), 1.61-1.71 (m, 2H), 1.74-1.83 (*m*, 3H), 1.86-1.90 (m, 1H), 1.97-2.02 (*m*, 2H), 2.12-2.17 (m, 2H), 2.22-2.27 (*m*, 2H), 2.31-2.35 (m, 1H), 2.58 (*s*, 1H), 2.82-2.90 (*m*, 2H), 3.55 (*pseudo dt*, J = 6.6, 8.8 Hz, 1H), 3.67 (*pseudo dt*, J = 6.2, 8.8 Hz, 1H), 3.78 (*s*, 3H), 4.95-4.98 (*m*, 1H), 5.02-5.05 (*m*, 1H), 5.81-5.88 (*m*, 1H), 6.63 (*d*, J = 2.8 Hz, 1H), 6.72 (*dd*, J = 2.8, 8.5 Hz, 1H), 7.22 (*d*, J = 8.5 Hz, 1H).

^{13}C NMR (150 MHz, $CDCl_3$) δ ppm: 12.7, 22.8, 26.5, 27.2, 29.4, 29.8, 30.5, 34.1, 37.0, 39.2, 43.5, 47.6, 49.4, 55.2, 64.7, 75.3, 84.9, 85.4, 111.5, 113.8, 114.5, 126.4, 132.7, 138.0, 138.6, 157.4.

HRMS (EI+) *m/z*: calculated for $C_{26}H_{34}O_2^+$ [M^+]: 378.2559; found: 378.2556.

17-*O*-Allyllynestrenol (6a)

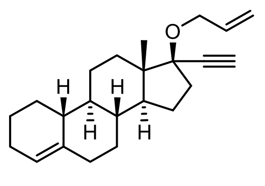

White powder (93% from **2** following procedure A).

M.p. = 80 °C.

[α]$_D$^{20} = +16 ($CHCl_3$, *c* 0.1).

^{1}**H NMR** (600 MHz, $CDCl_3$) δ ppm: 0.70 (*ddd*, J = 4.0, 10.6, 22.1 Hz, 1H), 0.84-0.96 (*m,* 4H), 1.05-1.42 (*m,* 6H), 1.51-1.61 (*ddd,* J = 7.6, 11.6, 11.6 Hz, 1H), 1.63-2.05 (*m,* 11H), 2.17-2.27 (*m,* 2H), 2.57 (*s,* 1H), 4.11 (*ddt*, J = 5.2, 12.6, 1.6 Hz, 1H), 4.17 (*ddt*, J = 5.2, 12.6, 1.6 Hz, 1H), 5.13 (*dq*, J = 1.6, 10.4 Hz, 1H), 5.29 (*dq*, J = 1.8, 17.2 Hz, 1H), 5.38-5.41 (*brs*, 1H), 5.94 (*ddt*, 5.2, 10.4, 17.2 Hz, 1H).

NMR ^{13}C (150 MHz, $CDCl_3$) δ ppm: 12.9, 22.1, 23.0, 25.5, 26.2, 28.8, 31.7, 34.1, 35.5, 37.1, 41.4, 42.0, 47.5, 49.5, 49.9, 66.6, 75.5, 85.1, 85.2, 115.7, 119.9, 135.4, 140.3.

HRMS (ESI+) *m/z*: calculated for $C_{23}H_{32}ONa^+$ [MNa^+]: 347.2345; found: 347.2332.

17-*O*-(4-Penten-1-yl)lynestrenol (6b)

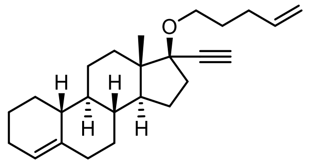

Brown resin (49% from **2** following procedure A).

$[\alpha]_D^{20}$ = +18 ($CHCl_3$, *c* 0.1).

^{1}**H NMR** (600 MHz, $CDCl_3$) δ ppm: 0.70 (*ddd*, J = 4.0, 10.8, 21.8 Hz, 1H), 0.83-0.93 (*m*, 5H), 1.08-1.24 (*m*, 3H) 1.27-1.40 (*m*, 3H), 1.52-1.58 (m, 2H), 1.62-1.71 (*m*, 4H), 1.78 (*dt*, J = 4.4, 12.8 Hz, 1H), 1.82-1.86 (m, 1H), 1.90-2.03 (*m*, 5H), 2.11-2.16 (m, 2H), 2.16-2.22 (*m*, 2H), 2.53 (*s*, 1H), 3.49-3.53 (*m*, 1H), 3.63 (*dt*, J = 8.8, 6.2 Hz, 1H), 4.94-4.97 (*m*, 1H), 5.01-5.04 (*dq*, J = 17.0, 1.7 Hz, 1H), 5.39 (*brs*, 1H), 5.80-5.87 (*m*, 1H).

^{13}**C NMR** (150 MHz, $CDCl_3$) δ ppm: 12.8, 22.1, 22.9, 25.5, 26.2, 28.8, 29.4, 30.5, 31.7, 34.0, 35.5, 37.0, 41.4, 42.0, 47.5, 49.4, 49.9, 64.7, 75.1, 84.9, 114.5, 119.9, 138.7, 140.4, 157.4.

HRMS (ESI+) *m/z*: calculated for $C_{25}H_{36}ONa^+$ [MNa^+]: 375.2658; found: 375.2642.

17-*O*-Allyldesogestrel (7)

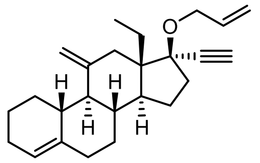

Beige powder (58% from **3** following procedure A).

M.p. = 70 °C.

$[\alpha]_D^{20}$ = +50 ($CHCl_3$, *c* 0.1).

^{1}**H NMR** (600 MHz, $CDCl_3$) δ ppm: 0.83-0.98 (*m*, 2H), 1.04 (*t*, J = 7.4 Hz, 3H), 1.12-1.19 (*m*, 1H), 1.31-1.48 (*m*, 6H), 1.61-1.69 (*m*, 3H), 1.88 (*ddd*, J = 12.7, 10.4, 7.6 Hz, 1H), 1.92-1.99 (*m*, 3H), 2.11 (*ddd*, J = 13.6, 12.1, 3.6 Hz, 1H), 2.18-2.29 (*m*, 4H), 2.34-2.36 (*d*, J = 12.3 Hz, 1H), 2.61 (*s*, 1H), 2.66 (*d*, J = 12.5 Hz, 1H), 4.08 (*ddt*, J = 12.7, 5.0, 1.6 Hz, 1H), 4.20 (ddt, J = 12.7, 5.1, 1.6 Hz, 1H), 4.77 (*brs*, 1H), 4.97 (*m*, 1H), 5.12 (*ddd*, J = 10.5, 3.3, 1.5 Hz, 1H), 5.29 (*dq*, J = 17.2, 1.8 Hz, 1H), 5.47 (*m*, 1H), 5.92 (*ddt*, J = 17.2, 10.3, 5.2 Hz, 1H).

^{13}**C NMR** (150 MHz, $CDCl_3$) δ ppm: 9.0, 20.2, 21.88, 21.95, 25.7, 29.1, 29.7, 31.7, 35.5, 36.6, 37.2, 41.4, 42.5, 51.0, 51.8, 54.7, 66.9, 75.9, 85.11, 86.4, 108.4, 115.5, 121.2, 135.3, 140.0, 147.7.

(2'*S*,8*R*,9*S*,13*S*,14*S*)-3-Methoxy-13-methyl-3'-vinyl-6,7,8,9,11,12,13,14,15,16-decahydro-5'*H*-spiro[cyclopenta[*a*]phenanthrene-17,2'-furan] (8a)

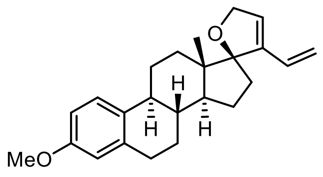

White powder (70% from **5a** following procedure B).

M.p. = 128-129 °C.

[α]$_D$^{20} = +9 ($CHCl_3$, *c* 0.1).

^{1}H NMR (600 MHz, $CDCl_3$) δ ppm : 0.96 (*s*, 3H), 1.28-1.39 (*m*, 1H), 1.42-1.60 (*m*, 6H), 1.69-1.79 (*m*, 1H), 1.87-1.94 (*m*, 1H), 1.95-2.07 (*m*, 2H), 2.07-2.14 (*m*, 1H), 2.21-2.27 (*m*, 1H), 2.76-2.93 (*m*, 2H), 3.77 (*s*, 3H), 4.53 (*s*, 2H), 5.13 (*dd*, J = 1.8, 10.8 Hz, 1H), 5.50 (*dd*, J = 1.8, 17.2 Hz, 1H), 5.96 (*s*, 1H), 6.18-6.29 (*m*, 1H), 6.62 (*d*, J = 2.7 Hz, 1H), 6.69 (*dd*, J = 2.7, 8.5 Hz, 1H), 7.17 (*d*, J = 8.5 Hz, 1H).

^{13}C NMR (150 MHz, $CDCl_3$) δ ppm : 15.4, 23.7, 26.4, 27.4, 29.9, 32.6, 34.4, 39.3, 43.7, 47.6, 49.4, 55.2, 72.7, 99.9, 111.4, 113.7, 116.1, 122.6, 126.3, 131.2, 132.7, 134.0, 144.4, 157.4.

HRMS (ESI+) *m/z*: calculated for $C_{24}H_{31}O_2^+$ [MH$^+$]: 351.2319; found: 351.2318.

(2'*S*,8*R*,9*S*,13*S*,14*S*)-3-Methoxy-13-methyl-3'-vinyl-6,6',7,7',8,9,11,12,13,14,15,16-dodecahydro-5'*H*-spiro[cyclopenta[*a*]phenanthrene-17,2'-oxepine] (8b)

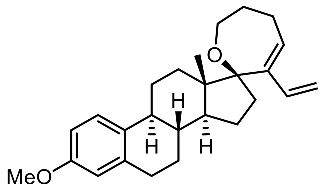

White powder (67% from **5b** following procedure B).

M.p. = 168 °C.

[α]$_D$^{20} = +14.6 ($CHCl_3$, *c* 0.1).

^{1}H NMR (600 MHz, $CDCl_3$) δ ppm : 0.96 (*s*, 3H), 1.25-1.32 (*m*, 1H), 1.45-1.60 (*m*, 5H), 1.66-1.73 (*m*, 2H), 1.80-1.94 (*m*, 5H), 2.06 (*dt*, J = 4.0, 11.5 Hz, 1H), 2.21-2.29 (*m*, 2H), 2.72 (*pseudo hept.*, J = 6.5 Hz, 1H), 2.79-2.88 (*m*, 2H), 3.60 (*ddd*, J = 6.4, 11.6, 11.6 Hz, 1H), 3.77 (*s*, 3H, OCH_3), 3.77-3.80 (m, 1H), 4.84 (*dd*, J = 2.0, 10.5 Hz, 1H), 5.28 (*dd*, J = 2.0, 16.4 Hz, 1H,), 6.05 (*dd*, J = 6.7, 8.9 Hz, 1H), 6.28 (dd, J = 10.5, 16.4 Hz, 1H), 6.61 (*d*, J = 2.7 Hz, 1H), 6.69 (*dd*, J = 2.7, 8.5 Hz, 1H), 7.18 (*d*, J = 8.5 Hz, 1H).

^{13}C NMR (150 MHz, $CDCl_3$) δ ppm : 14.9, 22.5, 24.1, 26.3, 26.35, 27.6, 29.8, 32.5, 34.0, 39.2, 43.6, 48.7, 48.9, 55.2, 61.7, 95.3, 111.3, 112.7, 113.7, 126.2, 126.4, 132.9, 137.9, 140.4, 143.5, 157.3.

HRMS (ESI+) *m/z*: calculated for $C_{26}H_{35}O_2^+$ [MH$^+$]: 379.2632; found: 379.2635.

(2'*S*,8*R*,9*S*,10*R*,13*S*,14*S*)-13-Methyl-3'-vinyl-1,2,3,6,7,8,9,10,11,12,13,14,15,16-tetradecahydro-5'*H*-spiro[cyclopenta[*a*]phenanthrene-17,2'-furan] (9a)

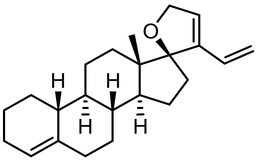

White powder (76% from **6a** following procedure B).

M.p. = 60 °C.

[α]D20 = +205 ($CHCl_3$, *c* 0.1).

1H NMR (600 MHz, $CDCl_3$) δ ppm : 0.57 (*ddd*, J = 4.2, 10.6, 21.7 Hz, 1H), 0.83-0.91 (*ddd*, J = 3.9, 12.8, 24.7 Hz, 1H), 0.96 (*s,* 3H), 1.04-1.11 (*m*, 1H), 1.17 (*ddd*, J = 3.3, 12.2, 24.7 Hz, 1H), 1.21-1.45 (*m,* 6H), 1.62-1.80 (*m,* 5H), 1.86-2.06 (*m,* 6H), 2.16-2.24 (*m,* 1H), 4.43-4.59 (*m,* 2H), 5.09 (*dd*, J = 1.9, 10.8 Hz, 1H), 5.39 (*brs,* 1H), 5.47 (*dd*, J = 1.8, 17.2 Hz, 1H), 5.93 (*s,* 1H), 6.18 (*dd*, J = 10.8, 17.2 Hz, 1H).

13C NMR (150 MHz, $CDCl_3$) δ ppm: 15.5, 22.1, 23.8, 25.5, 26.0, 28.8, 31.8, 32.6, 34.4, 35.5, 41.6, 42.0, 47.5, 49.4, 50.1, 72.6, 99.9, 115.9, 119.9, 122.4, 131.2, 140.2, 144.5.

HRMS (ESI+) *m/z*: calculated for $C_{23}H_{33}O^+$ [MH^+]: 325.2526; found: 325.2515.

(2'*S*,8*R*,9*S*,10*R*,13*S*,14*S*)-13-Methyl-3'-vinyl-1,2,3,6,6',7,7',8,9,10,11,12,13,14,15,16-hexadecahydro-5'*H*-spiro[cyclopenta[*a*]phenanthrene-17,2'-oxepine] (9b)

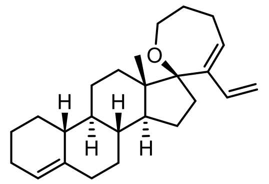

Colorless resin (80% from **6b** following procedure B).

[α]D20 = +126 ($CHCl_3$, *c* 0.1).

1H NMR (600 MHz, $CDCl_3$) δ ppm : 0.52 (*ddd*, J = 4.3, 10.9, 21.6 Hz, 1H), 0.78-0.90 (*m*, 3H), 0.96 (*s*, 3H), 1.02-1.09 (*m*, 2H), 1.14-1.28 (*m*, 2H), 1.30-1.50 (*m*, 4H), 1.50-1.62 (*m*, 2H), 1.63-1.69 (*m*, 3H), 1.84-2.02 (*m*, 6H), 2.17-2.23 (*m*, 2H), 2.68 (*ddd*, J = 6.5, 12.8, 19.3 Hz, 1H), 3.57 (*ddd*, J = 6.3, 11.2, 12.0 Hz, 1H), 3.76 (*dd*, J = 8.0, 12.2 Hz, 1H), 4.79 (*dd*, J = 2.1, 10.5 Hz, 1H), 5.24 (*dd*, J = 2.1, 16.5 Hz, 1H), 5.38 (*brs*, 1H), 6.01 (*dd*, J = 6.5, 8.9 Hz, 1H), 6.23 (*dd*, J = 10.4, 16.5 Hz, 1H).

13C NMR (150 MHz, $CDCl_3$) δ ppm: 15.0, 22.1, 22.4, 24.2, 25.5, 26.0, 26.3, 28.8, 31.9, 32.5, 34.0, 35.5, 41.4, 42.0, 48.6, 48.8, 50.1, 61.6, 95.4, 112.6, 119.7, 126.2, 140.4, 140.7, 143.5.

HRMS (ESI+) *m/z*: calculated for $C_{25}H_{37}O^+$ [MH^+]: 353.2839; found: 353.2854.

(2'*S*,8*S*,9*S*,10*R*,13*S*,14*S*)-13-Ethyl-11-methylene-3'-vinyl-1,2,3,6,7,8,9,10,11,12,13,14,15,16-tetradecahydro-5'*H*-spiro[cyclopenta[*a*]phenanthrene-17,2'-furan] (10)

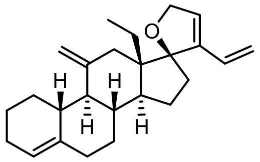

Colorless resin (82% from **7** following procedure A).

1H NMR (600 MHz, $CDCl_3$) δ ppm: 0.87-0.94 (*m*, 1H), 1.04 (*t*, J = 7.2 Hz, 3H), 1.06-1.12 (*m*, 1H), 1.20-1.24 (*m*, 1H), 1.26 (*brs*, 1H), 1.36-1.49 (*m*, 4H), 1.57-1.64 (*m*, 3H), 1.70 (*dq*, J = 12.3, 3.4 Hz, 1H), 1.77 (*d*, J = 12.3 Hz, 1H), 1.93-1.98 (*m*, 3H), 2.00-2.08

(*m*, 2H), 2.18-2.26 (*m*, 3H), 2.35 (*d*, J = 12.3 Hz, 1H), 4.46 (*d*, J = 13.6 Hz, 1H), 4.51 (*dt*, J = 13.6, 1.8 Hz, 1H), 4.73 (*brs*, 1H), 4.92 (*m*, 1H), 5.13 (*dd*, J = 10.7, 1.7 Hz, 1H), 5.46-5.51 (*m*, 2H), 5.98 (*brs*, 1H), 6.23 (*ddq*, J = 17.2, 10.8, 1.3 Hz, 1H).

^{13}C NMR (150 MHz, $CDCl_3$) δ ppm: 9.1, 21.4, 22.0, 22.8, 25.7, 29.1, 31.8, 35.1, 35.5, 36.6, 40.5, 42.6, 51.4, 52.4, 54.8, 72.2, 100.4, 108.1, 116.3, 121.2, 122.8, 131.0, 140.1, 144.7, 148.0.

HRMS (ESI+) *m/z*: calculated for $C_{25}H_{35}O^+$ [MH^+]: 351.2682; found: 351.2692.

(3'*S*,5a'*S*,8*R*,8a'*R*,8b'*S*,9*S*,13*S*,14*S*)-3-Methoxy-13-methyl-7'-phenyl-1',5',5a',6,7,8,8b',9,11,12,13,14,15,16-tetradecahydrospiro[cyclopenta[*a*]phenanthrene-17,3'-furo[3,4-*e*]isoindole]-6',8'(7'*H*,8a'*H*)-dione (16a)

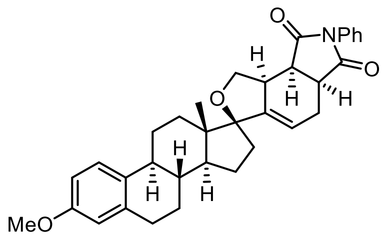

Pale brown powder (83% from **5a** following procedure C).

M.p. = 107-108 °C

$[\alpha]_D^{20}$ = +15 ($CHCl_3$, *c* 0.1).

NMR ^{1}H (600 MHz, $CDCl_3$) δ ppm : 0.97 (*s*, 3H), 1.29-1.55 (*m*, 6H), 1.63-1.75 (*m*, 3H), 1.86-1.95 (*m*, 1H), 2.09-2.31 (*m*, 4H), 2.81-2.94 (*m*, 3H), 3.00-3.08 (*m*, 1H), 3.33 (*td*, J = 1.8, 6.0 Hz, 1H), 3.40 (*t*, J = 8.5 Hz, 1H), 3.78 (*s*, 3H), 4.03-4.07 (*dd*, J = 7.8, 9.6 Hz, 1H), 4.73 (*dd*, J = 2.1, 9.6 Hz, 1H), 5.74-5.79 (*m*, 1H), 6.64 (*d*, J = 2.8 Hz, 1H), 6.71 (*dd*, J = 2.8, 8.6 Hz, 1H), 7.16 (*d*, J = 8.6 Hz, 1H), 7.19-7.22 (*m*, 2H), 7.36 (*tt*, J = 1.2, 6.8 Hz, 1H), 7.43 (*t*, J = 7.5 Hz, 2H).

NMR ^{13}C (150 MHz, $CDCl_3$) δ ppm : 13.6, 22.9, 25.1, 26.6, 27.2, 29.8, 35.1, 35.3, 39.1, 39.9, 40.8, 41.2, 43.4, 47.1, 49.1, 55.2, 65.8, 94.7, 111.5, 113.7, 117.0, 126.2, 126.4 (2C), 128.5, 129.0 (2C), 131.9, 132.4, 137.9, 151.4, 157.4, 176.2, 178.4.

HRMS (ESI+) *m/z*: calculated for $C_{34}H_{38}NO_4^+$ [MH^+]: 524.2795; found: 524.2811.

(3*S*,5a*S*,8a*R*,8b*S*,8'*R*,9'*S*,13'*S*,14'*S*)-3'-Methoxy-13'-methyl-5,5a,6',7',8',9',11',12',13',14',15',16'-dodecahydro-1*H*-spiro[benzo[1,2-*c*:3,4-*c*']difuran-3,17'-cyclopenta[*a*]phenanthrene]-6,8(8a*H*,8b*H*)-dione (16b)

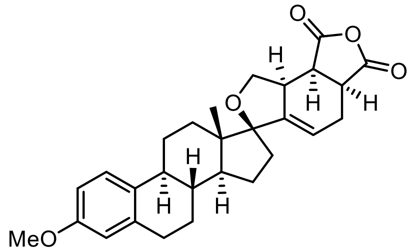

White powder (10% from **5a** following procedure C).

M.p. = 220 °C

$[\alpha]_D^{20}$ = +13 ($CHCl_3$, *c* 0.1).

NMR ^{1}H (600 MHz, $CDCl_3$) δ ppm: 0.95 (*s*, 3H), 1.29-1.38 (*m*, 2H), 1.39-1.54 (*m*, 4H), 1.63-1.76 (*m*, 3H), 1.88-1.94 (*m*, 1H), 2.07-2.17 (*m*, 2H), 2.18-2.23 (*m*, 1H), 2.27-2.31 (*m*, 1H), 2.76-2.80 (*m*, 1H), 2.82-2.88 (*m*, 2H), 2.93 (*ddd*, J = 1.7, 7.4, 15.2 Hz, 1H), 3.43-3.44 (*m*, 1H), 3.54 (*dd*, J = 8.0, 9.6 Hz, 1H), 3.78 (*s*, 3H), 4.06 (*dd*, J = 8.1, 9.9

Hz, 1H), 4.54 (*dd*, J = 2.6, 9.8 Hz, 1H), 5.79 (*dt*, J = 3.1, 7.2 Hz, 1H), 6.63 (*d*, J = 2.8 Hz, 1H), 6.70 (*dd*, J = 2.8, 8.6 Hz, 1H), 7.15 (*d*, J = 8.6 Hz, 1H).

NMR ^{13}C (150 MHz, $CDCl_3$) δ ppm: 13.6, 22.9, 25.1, 26.5, 27.2, 29.8, 34.7, 35.0, 39.0, 40.1 (2C), 42.0, 43.4, 47.2, 49.0, 55.2, 65.7, 94.8, 111.5, 113.7, 116.5, 126.2, 132.3, 137.9, 152.0, 157.5, 170.7, 174.0.

HRMS (ESI+) *m/z*: calculated for $C_{28}H_{33}O_5^+$ [MH^+]: 449.2323; found: 449.2334.

(1'*S*,3a'*R*,8*R*,9*S*,13*S*,14*S*)-Dimethyl 3-methoxy-13-methyl-3a',6,6',7,8,9,11,12,13,14,15,16-dodecahydro-3'*H*-spiro[cyclopenta[*a*]phenanthrene-17,1'-isobenzofuran]-4',5'-dicarboxylate (16c)

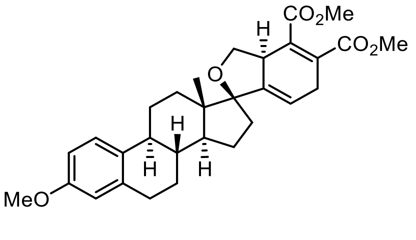

White solid (73% from **5a** following procedure C).

M.p. = 63.6 °C

[α]$_D^{20}$ = +8 ($CHCl_3$, *c* 0.1).

NMR ^{1}H (600 MHz, $CDCl_3$) δ ppm : 0.92 (*s,* 3H), 1.20-1.26 (*m*, 1H), 1.29-1.39 (*m*, 1H), 1.43-1.51 (*m,* 4H), 1,61-1,65 (*m,* 1H), 1.70-1.75 (*m,* 1H), 1.90-1.95 (*m,* 2H), 2.07-2.15 (*m,* 2H), 2.21-2.29 (*m,* 1H), 2.80-2.93 (*m*, 2H), 3.16-3.24 (*m,* 2H), 3.43-3.57 (*m,* 2H), 3.78 (*s*, 3H), 3.80 (*s*, 3H), 3.81 (*s*, 3H), 4.23 (*t*, J = 7.1 Hz, 1H), 5.55 (*m*, 1H), 6.63 (*d*, J = 2.8 Hz, 1H), 6.71 (*dd*, J = 2.8, 8.6 Hz, 1H), 7.18 (*d*, J = 8.7 Hz, 1H).

NMR ^{13}C (150 MHz, $CDCl_3$) δ ppm: 14.1, 22.9, 26.2, 27.3, 29.6, 29.9, 32.8, 36.2, 39.2, 41.3, 43.7, 46.6, 48.5, 52.3, 52.4, 55.2, 68.8, 94.1, 111.5, 113.8, 115.0, 126.3, 132.1, 132.6, 135.1, 138.0, 144.5, 157.4, 167.4, 168.4.

HRMS (ESI+) *m/z*: calculated for $C_{30}H_{37}O_6^+$ [MH^+]: 493.2585; found: 493.2599.

(3'*S*,8*R*,9*S*,9b'*R*,13*S*,14*S*)-3-Methoxy-13-methyl-5',6,7,8,9,9b',11,12,13,14,15,16-dodecahydro-1'*H*-spiro[cyclopenta[*a*]phenanthrene-17,3'-naphtho[1,2-*c*]furan]-6',9'-diol (16d)

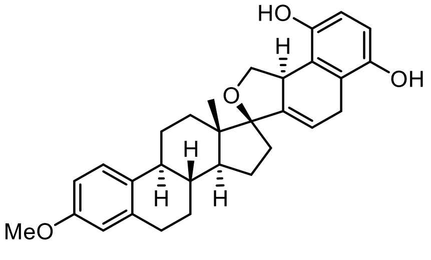

White powder (61% from **5a** following procedure C).

[α]$_D^{20}$ = +14 ($CHCl_3$, *c* 0.1).

M.p. = 116-118 °C

NMR ^{1}H (400 MHz, $CDCl_3$) δ ppm : 0.98 (*s*, 3H), 1.26-1.60 (*m*, 6H), 1.67-1.79 (*m*, 2H), 1.92-2.03 (*m*, 2H), 2.11-2.26 (*m*, 3H), 2.82-2.94 (*m*, 2H), 3.34 (*dd*, J = 7.8, 21.4 Hz, 1H), 3.51-3.60 (*m*, 2H), 3.73-3.83 (*m*, 4H), 4.81 (*t*, J = 7.8 Hz, 1H), 5.24 (br. *s,* 1H), 5.45 (br. *s,* 1H), 5.70-5.74 (*m*, 1H), 6.47 (*d*, J = 8.5 Hz, 1H), 6.54 (*d*, J = 8.5 Hz, 1H), 6.64 (*d*, J = 2.7 Hz, 1H), 6.70 (*dd*, J = 2.7, 8.5 Hz, 1H), 7.19 (*d*, J = 8.5 Hz, 1H).

NMR ^{13}C (150 MHz, $CDCl_3$) δ ppm : 14.2, 23.0, 25.5, 26.4, 26.9, 27.4, 29.9, 33.0, 36.3, 39.3, 40.0, 43.7, 46.5, 48.4, 55.2, 70.5, 93.4, 111.4, 112.8, 112.9, 113.7, 116.0, 122.7, 123.1, 132.8, 138.0, 145.2, 146.8, 147.6, 157.4.

(3*S*,5a*S*,8'*R*,9'*S*,11a*R*,11b*S*,13'*S*,14'*S*)-3'-Methoxy-13'-methyl-5,5a,6',7',8',9',11',12',13',14',15',16'-dodecahydro-1*H*-spiro[anthra[1,2-*c*]furan-3,17'-cyclopenta[*a*]phenanthrene]-6,11(11a*H*,11b*H*)-dione (16e)

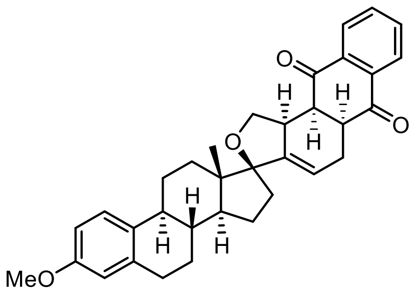

White amorphous solid (91% from **5a** following procedure C).

$[\alpha]_D^{20}$ = +36 ($CHCl_3$, *c* 0.1).

M.p. = 129.4 °C

NMR ^{1}H (400 MHz, $CDCl_3$) δ ppm : 0.96 (*s,* 3H), 1.30-1.38 (*m*, 2H), 1.43-1.53 (*m,* 4H), 1.62 (*dt*, 3.2, 11.7 Hz, 1H), 1.67-1.71 (*m*, 1H), 1.89-1.97 (*m,* 2H), 2.11-2.21 (*m*, 2H), 2.24-2.32 (*m*, 2H), 2.55-2.60 (*m*, 1H), 2.82-2.91 (*m*, 2H), 2.95-3.00 (*m*, 1H), 3.36 (*ddd*, J = 4.7, 6.6, 10.9 Hz, 1H), 3.70 (*t*, J = 5.1 Hz, 1H), 3.78 (*s*, 3H), 4.00 (*dd*, J = 7.9, 9.1 Hz, 1H), 4.38 (*t*, J = 8.1 Hz, 1H), 5.44 (*q*, J = 3.5 Hz, 1H), 6.63 (*d*, J = 2.7 Hz, 1H), 6.71 (*dd*, J = 2.7, 8.5 Hz, 1H), 7.19 (*d*, J = 8.5 Hz, 1H), 7.73-7.76 (*m*, 2H), 7.96-7.94 (*m*, 2H).

NMR ^{13}C (150 MHz, $CDCl_3$) δ ppm : 14.2, 23.0, 26.4, 26.5, 27.3, 29.9, 33.3, 35.4, 39.2, 41.6, 43.6, 46.6, 46.8, 48.5, 48.8, 55.2, 66.7, 94.7, 111.4, 113.7, 115.1, 126.3, 126.5, 127.0, 132.7 (2C^q), 134.1, 134.5, 135.6, 138.0, 146.3, 157.4, 196.4, 198.7.

HRMS (ESI+) *m/z*: calculated for $C_{34}H_{37}O_4^+$ [MH^+]: 509.2686; found: 509.2706.

(3a'*S*,6'*S*,8*R*,9*S*,10a'*S*,10b'*R*,13*S*,14*S*)-3-Methoxy-13-methyl-2'-phenyl-3a',4',6,7,8,8',9,9',10',10a',11,12,13,14,15,16-hexadecahydrospiro[cyclopenta[*a*]phenanthrene-17,6'-oxepino[4,3-e]isoindole]-1',3'(2'*H*,10b'*H*)-dione (16f)

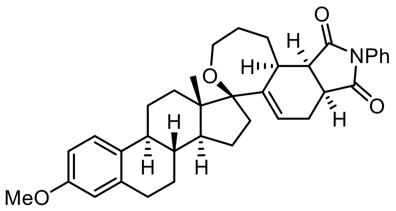

White powder (14% from **5b**, isolated from a mixture of several stereoisomeric cycloadducts, global yield: 65%).

M.p. = 280 °C

$[\alpha]_D^{20}$ = –9 ($CHCl_3$, *c* 0.1).

NMR ^{1}H (600 MHz, $CDCl_3$) δ ppm : 0.97 (*s*, 3H), 1.03 (*dt*, J = 3.9, 12.4 Hz, 1H), 1.11-1.18 (*m*, 1H), 1.31-1.57 (*m*, 7H), 1.66-1.76 (*m*, 3H), 1.88-1.92 (*m*, 1H), 1.94-1.99 (*m*, 1H), 2.06-2.12 (*m*, 2H), 2.23-2.27 (*m*, 1H), 2.51 (*ddd*, J = 2.2, 8.1, 17.5 Hz, 1H), 2.82-2.91 (*m*, 3H), 3.25-3.34 (*m*, 4H), 3.78 (*s*, 3H), 3.79-3.82 (*m*, 1H), 5.70 (*dd*, J = 2.0, 7.2

Hz, 1H), 6.63 (*d*, J = 2.6 Hz, 1H), 6.71 (*dd*, J = 2.6, 8.6 Hz, 1H), 7.18 (*d*, J = 8.6 Hz, 1H), 7.30-7.33 (*m*, 2H), 7.40-7.43 (*m*, 1H), 7.48-7.51 (*m*, 2H).

NMR ^{13}C (150 MHz, $CDCl_3$) δ ppm: 14.5, 22.2, 23.3, 26.3, 27.4, 28.1, 29.8 (2C), 34.0, 35.0, 37.8, 38.7, 39.2, 43.7, 44.1, 47.4, 48.2, 55.2, 65.5, 92.2, 111.4, 113.8, 123.6, 126.2, 126.4 (2C), 128.6, 129.2 (2C), 131.9, 132.5, 137.9, 149.2, 157.4, 178.1, 179.5.

HRMS (ESI+) *m/z*: calculated for $C_{36}H_{42}NO_4^+$ [MH^+]: 552.3108; found: 552.3122.

(3'*S*,5a'*S*,8*R*,8a'*R*,8b'*S*,9*S*,10*R*,13*S*,14*S*)-13-Methyl-7'-phenyl-1,1',2,3,5',5a',6,7,8,8b',9,10,11,12,13,14,15,16-octadecahydrospiro[cyclopenta[*a*]phenanthrene-17,3'-furo[3,4-*e*]isoindole]-6',8'(7'*H*,8a'*H*)-dione (17a)

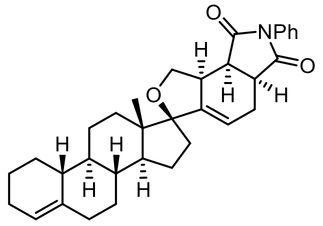

Brown powder (84% from **6a** following procedure C).

M.p. = 249 °C.

[α]$_D^{20}$ = –50 ($CHCl_3$, *c* 0.1).

^{1}H NMR (600 MHz, $CDCl_3$) δ ppm : 0.57-0.64 (*m*, 1H), 0.83-0.92 (*m*, 1H), 0.96 (*s*, 3H), 1.02-1.11 (*m*, 1H), 1.14-1.24 (*m*, 3H), 1.27-1.39 (*m*, 3H), 1.52-1.58 (m, 2H), 1.60-1.64 (*m*, 1H), 1.64-1.78 (*m*, 4H), 1.85-2.03 (*m*, 4H), 2.10 (*ddd*, J = 2.9, 11.3, 14.0 Hz, 1H), 2.17-2.26 (*m*, 2H), 2.76-2.83 (*m*, 1H), 3.00 (*ddd*, J = 2.0, 7.4, 14.7 Hz, 1H), 3.30 (*ddd*, J = 1.9, 6.0, 8.4 Hz, 1H), 3.37 (*t*, J = 8.4 Hz, 1H), 4.01 (*dd*, J = 7.7, 9.5 Hz, 1H), 4.69 (*dd*, *J* = 2.3, 9.6 Hz, 1H), 5.37-5.41 (*m*, 1H), 5.68 (*ddd*, *J* = 3.2, 7.3, 7.3 Hz, 1H), 7.15-7.21 (*m*, 2H), 7.32-7.37 (*m*, 1H), 7.39-7.44 (*m*, 2H).

NMR ^{13}C (150 MHz, $CDCl_3$) δ ppm: 13.6, 22.0, 23.1, 25.0, 25.5, 26.2, 28.7, 31.7, 35.0, 35.2, 35.4, 39.9, 40.8, 41.2, 41.8, 47.0, 49.1, 49.9, 65.8, 94.7, 116.7, 119.9, 126.4 (3C), 128.5, 129.0 (2C), 131.9, 140.2, 151.4, 176.2, 178.4.

HRMS (ESI+) *m/z*: calculated for $C_{33}H_{40}NO_3^+$ [MH^+]: 498.3003; found: 498.2988.

(3a'*S*,6'*S*,8*R*,9*S*,10*R*,10a'*S*,10b'*R*,13*S*,14*S*)-13-Methyl-2'-phenyl-1,2,3,3a',4',6,7,8,8',9,9',10,10',10a',11,12,13,14,15,16-icosahydrospiro[cyclopenta[*a*]phenanthrene-17,6'-oxepino[4,3-*e*]isoindole]-1',3'(2'*H*,10b'*H*)-dione (17b)

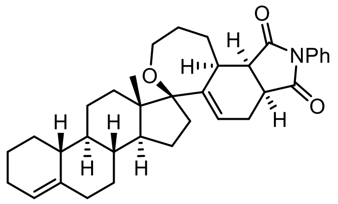

White powder (10% from **6b**, isolated from a mixture of several stereoisomeric cycloadducts, global yield: 48%).

M.p. = 194 °C

[α]$_D^{20}$ = +6 ($CHCl_3$, *c* 0.1).

NMR ^{1}H (600 MHz, $CDCl_3$) δ ppm: 0.36 (*ddd*, J = 4.4, 10.9, 22.2 Hz, 1H), 0.50 (*dt*, J = 4.3, 12.7 Hz, 1H), 0.78-0.87 (*m*, 2H), 0.88 (*s,* 3H), 0.97-1.04 (*m*, 1H), 1.08 (*ddd,* J = 8.0, 11.7, 11.7 Hz, 1H), 1.19 (*ddd*, J = 2.9, 11.0, 21.6 Hz, 1H), 1.27-1.35 (*m,* 3H), 1.41-

1.45 (*m*, 1H), 1.51-1.56 (*m*, 2H), 1.59-1.67 (*m,* 4H), 1.73-1.78 (*m,* 1H), 1.82-1.99 (*m,* 7H), 2.19 (*ddd*, J = 2.6, 3.8, 13.7 Hz, 1H), 2.47 (*ddd,* J = 2.0, 8.1, 16.6 Hz, 1H), 2.97 (*ddd*, J = 1.1, 7.3, 16.5 Hz, 1H), 3.13 (*dd*, J = 0.5, 9.9 Hz, 1H), 3.20-3.25 (*m*, 2H), 3.29-3.33 (*m*, 1H), 3.78-3.82 (*m*, 1H), 5.37 (*brs*, 1H), 5.56 (*dd*, *J* = 2.2, 7.4 Hz, 1H), 7.27-7.29 (*m*, 2H), 7.34-7.37 (*m*, 1H), 7.41-7.44 (*m*, 2H).

NMR ^{13}C (150 MHz, $CDCl_3$) δ ppm : 14.7, 22.2, 23.0, 23.6, 25.6, 28.5, 29.6, 31.7, 34.0, 35.5, 36.1, 37.3, 37.6, 38.9, 41.5, 42.0, 47.3, 47.7, 48.4, 49.8, 65.5, 92.3, 119.8, 123.1, 125.9 (3C), 128.3, 129.1 (2C), 132.1, 140.3, 149.2, 178.5, 179.3.

HRMS (ESI+) *m/z*: calculated for $C_{35}H_{44}NO_3^+$ [MH^+]: 526.3316; found: 526.3334.

17-*O*-Allylmestranol (5a): ^{1}H NMR (600 MHz, $CDCl_3$)

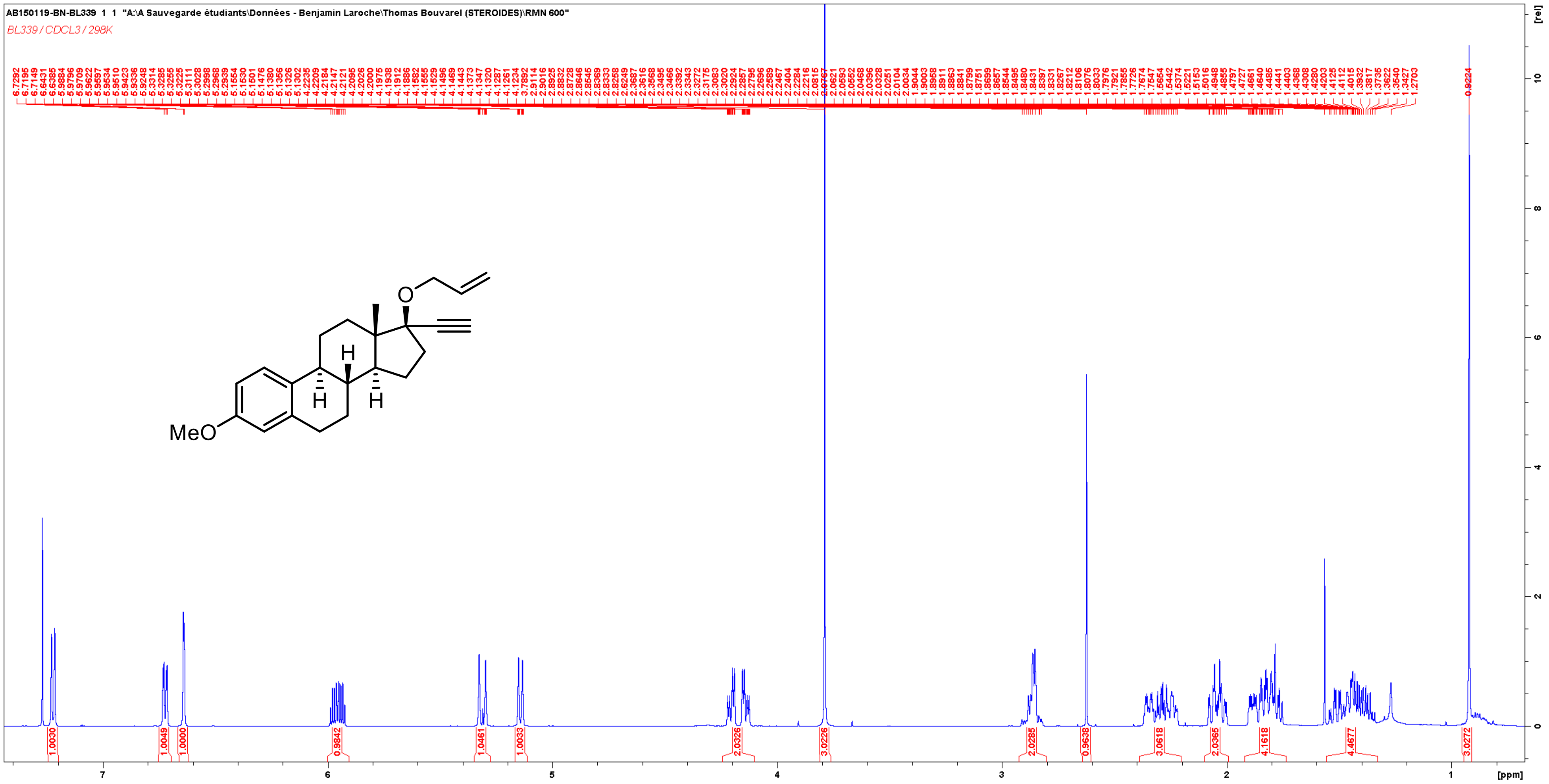

17-*O*-Allylmestranol (5a): ^{13}C NMR (150 MHz, $CDCl_3$)

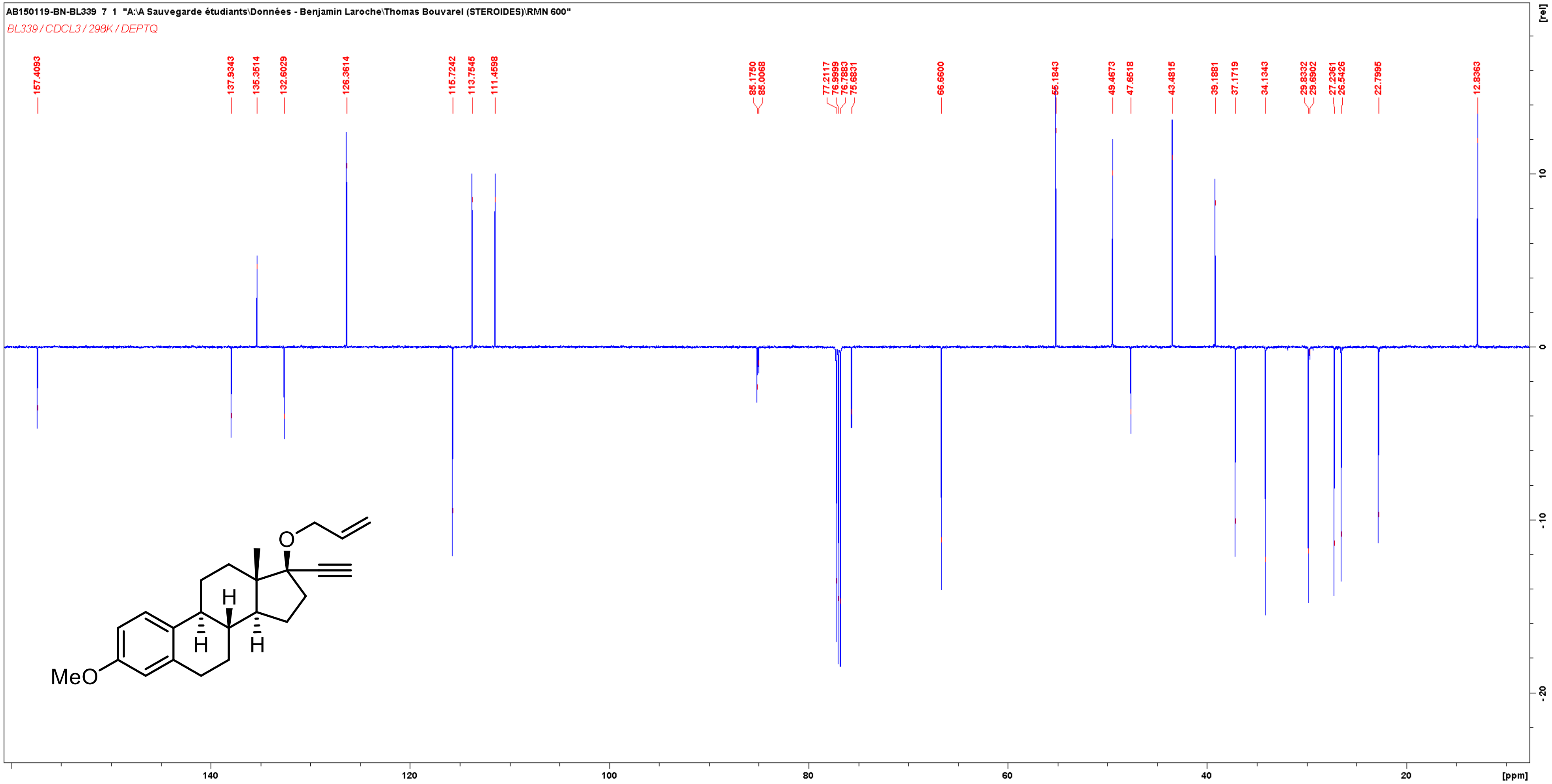

17-*O*-(4-Penten-1-yl)mestranol (5b): ^{1}H NMR (600 MHz, $CDCl_3$)

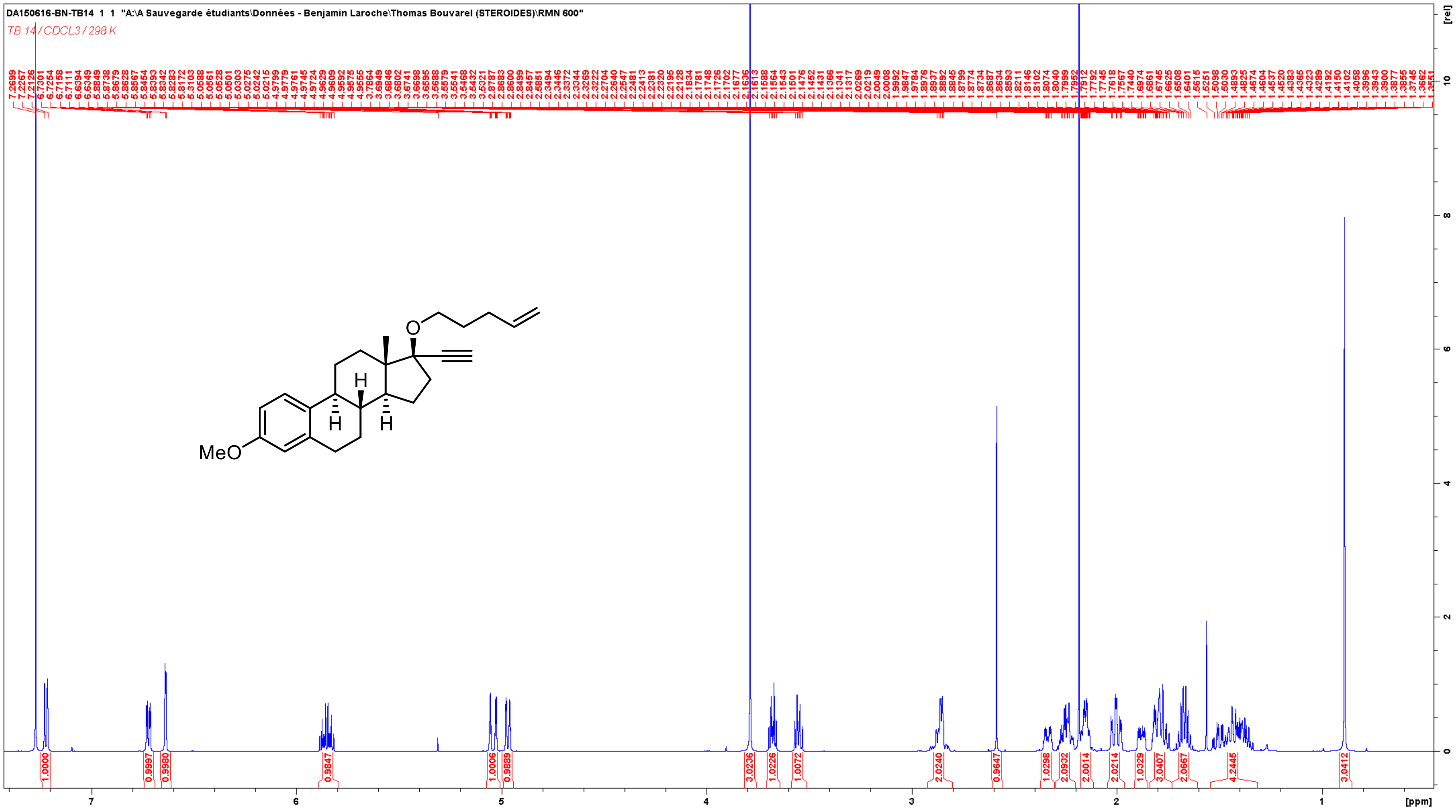

17-*O*-(4-Penten-1-yl)mestranol (5b): ^{13}C NMR (150 MHz, $CDCl_3$)

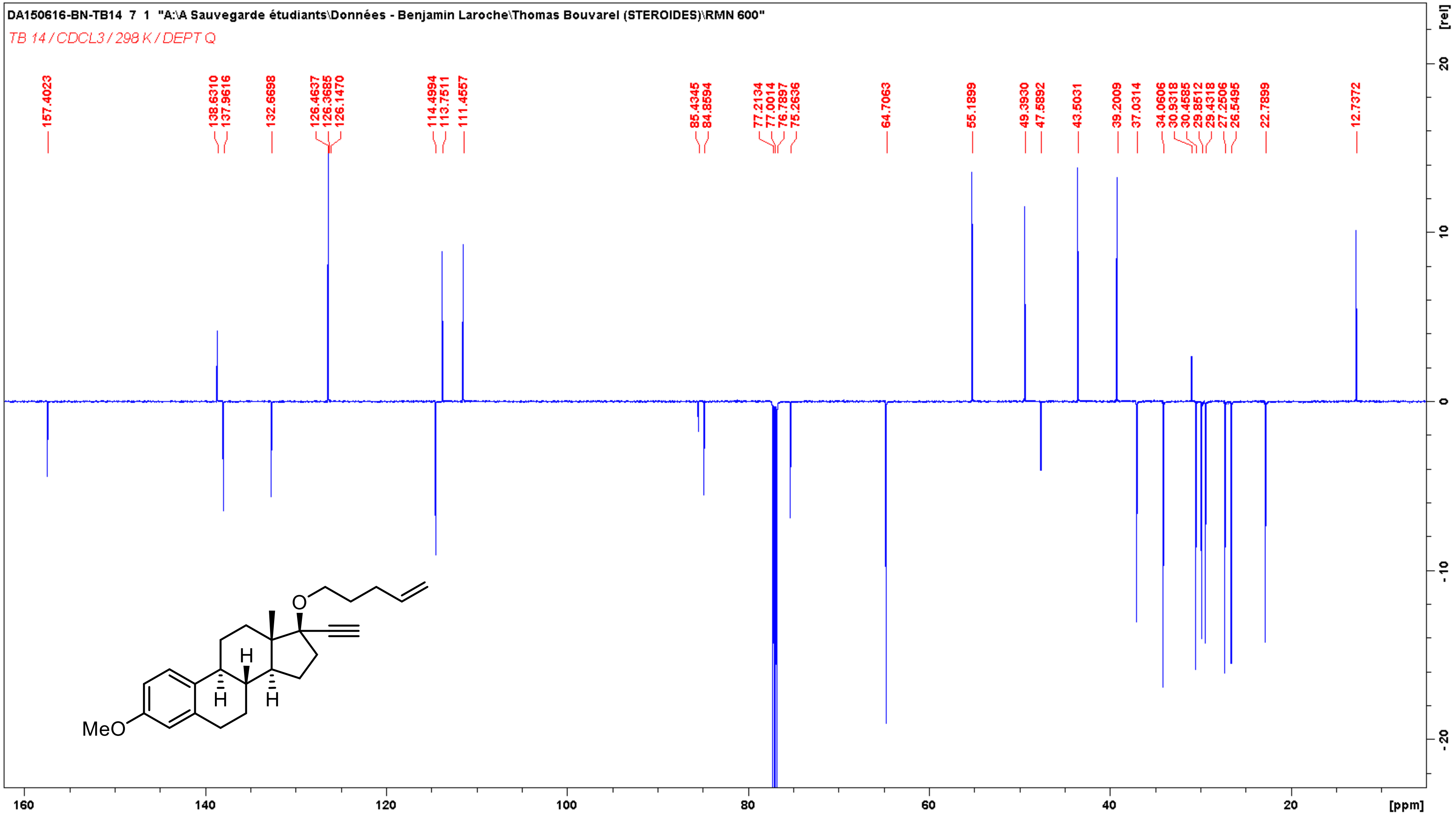

17-*O*-Allyllynestrenol (6a): ^{1}H NMR (600 MHz, $CDCl_3$)

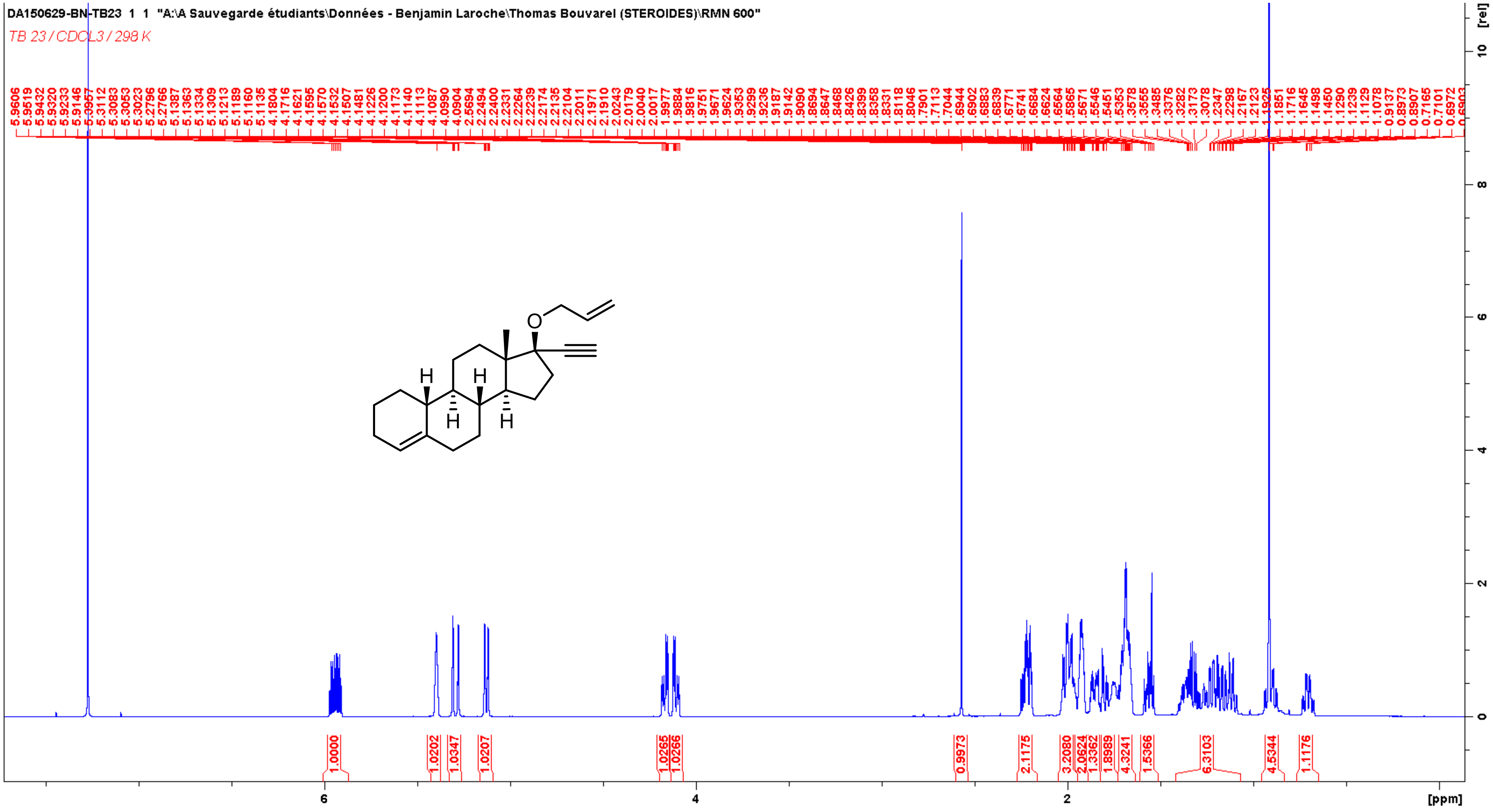

17-*O*-Allyllynestrenol (6a): ^{13}C NMR (150 MHz, $CDCl_3$)

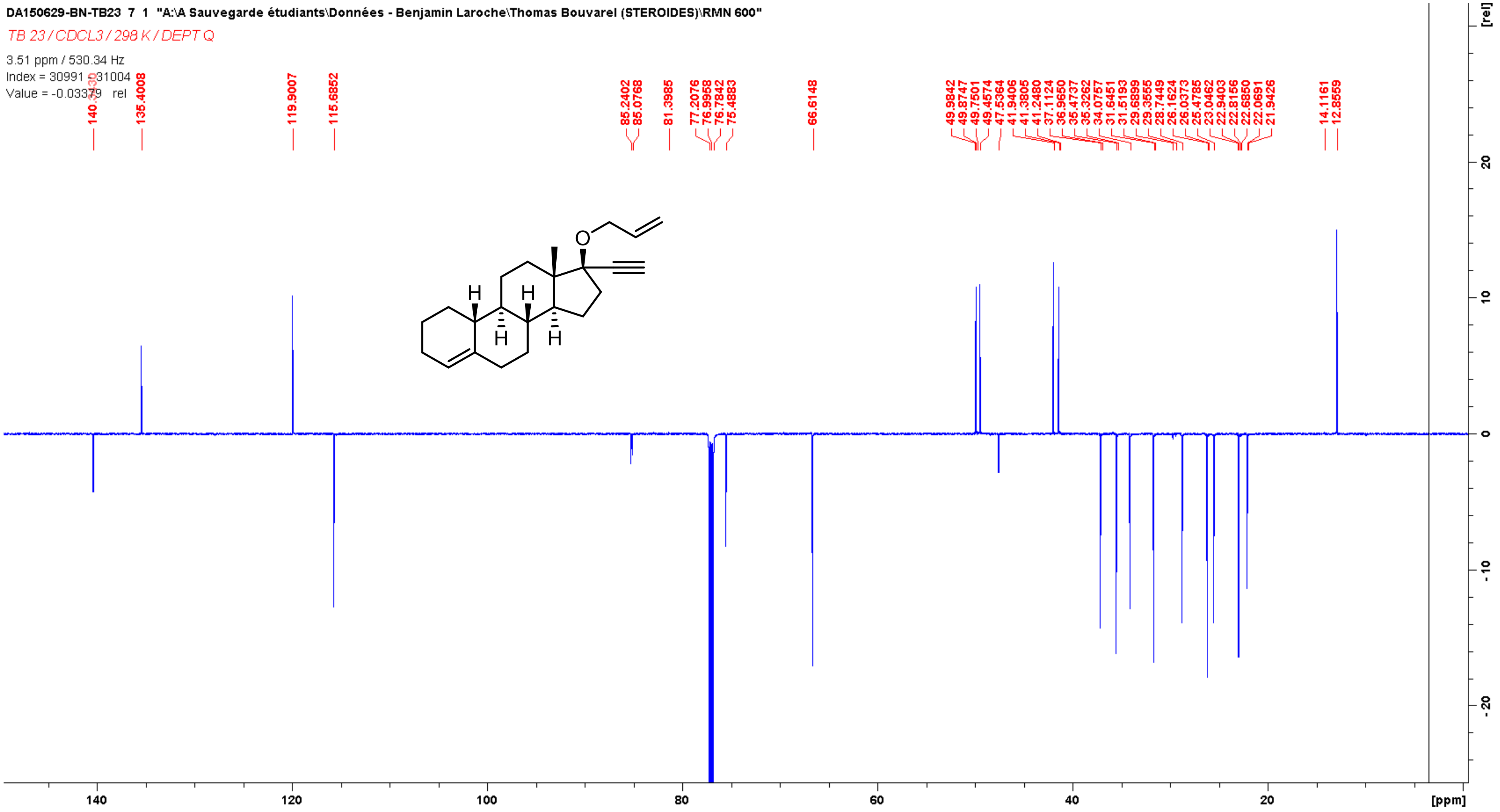

17-*O*-(4-Penten-1-yl)lynestrenol (6b): ^{1}H NMR (600 MHz, $CDCl_3$)

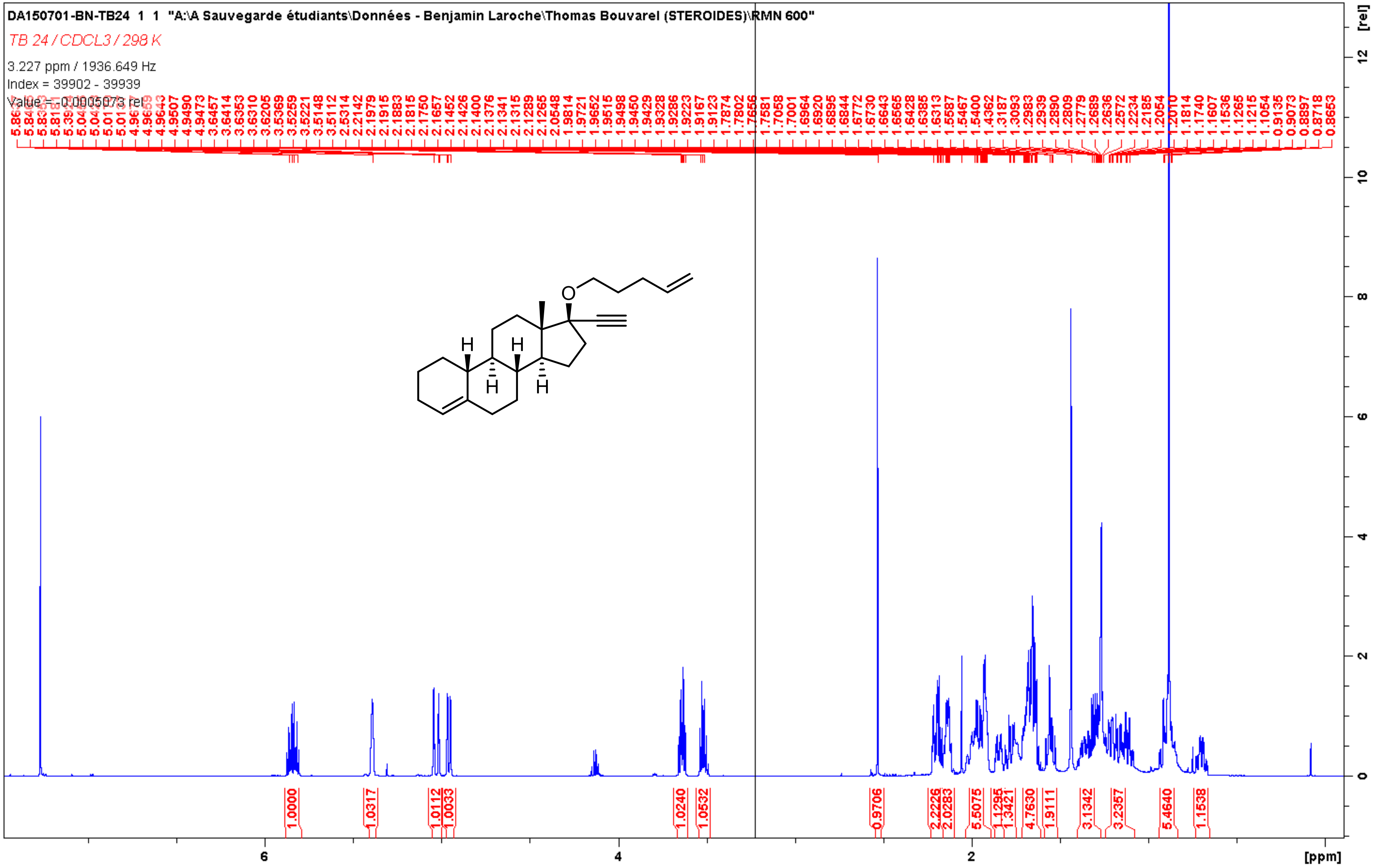

17-*O*-(4-Penten-1-yl)lynestrenol (6b): ^{13}C NMR (150 MHz, $CDCl_3$)

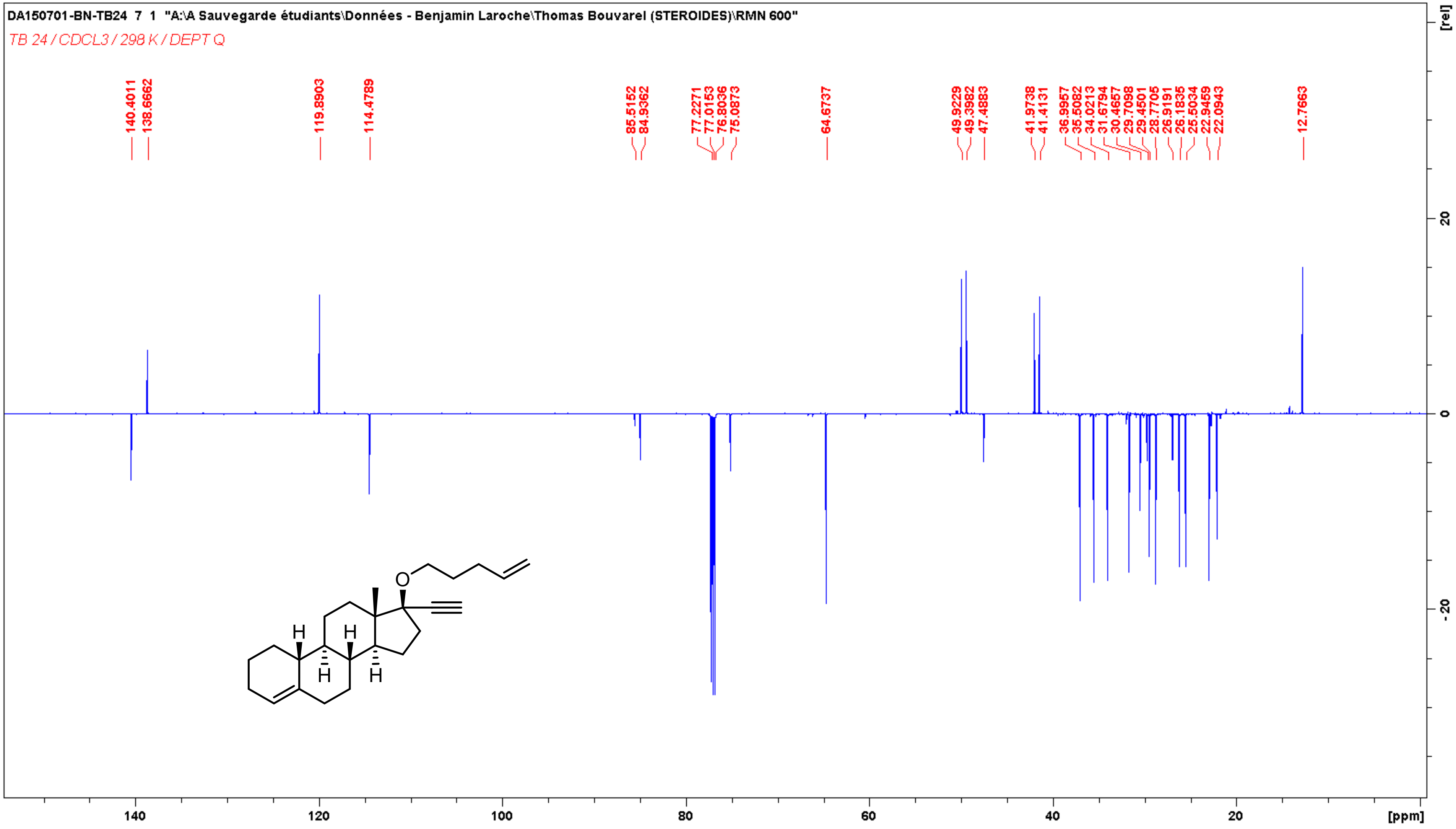

17-*O*-Allyldesogestrel (7): ^{1}H NMR (600 MHz, $CDCl_3$)

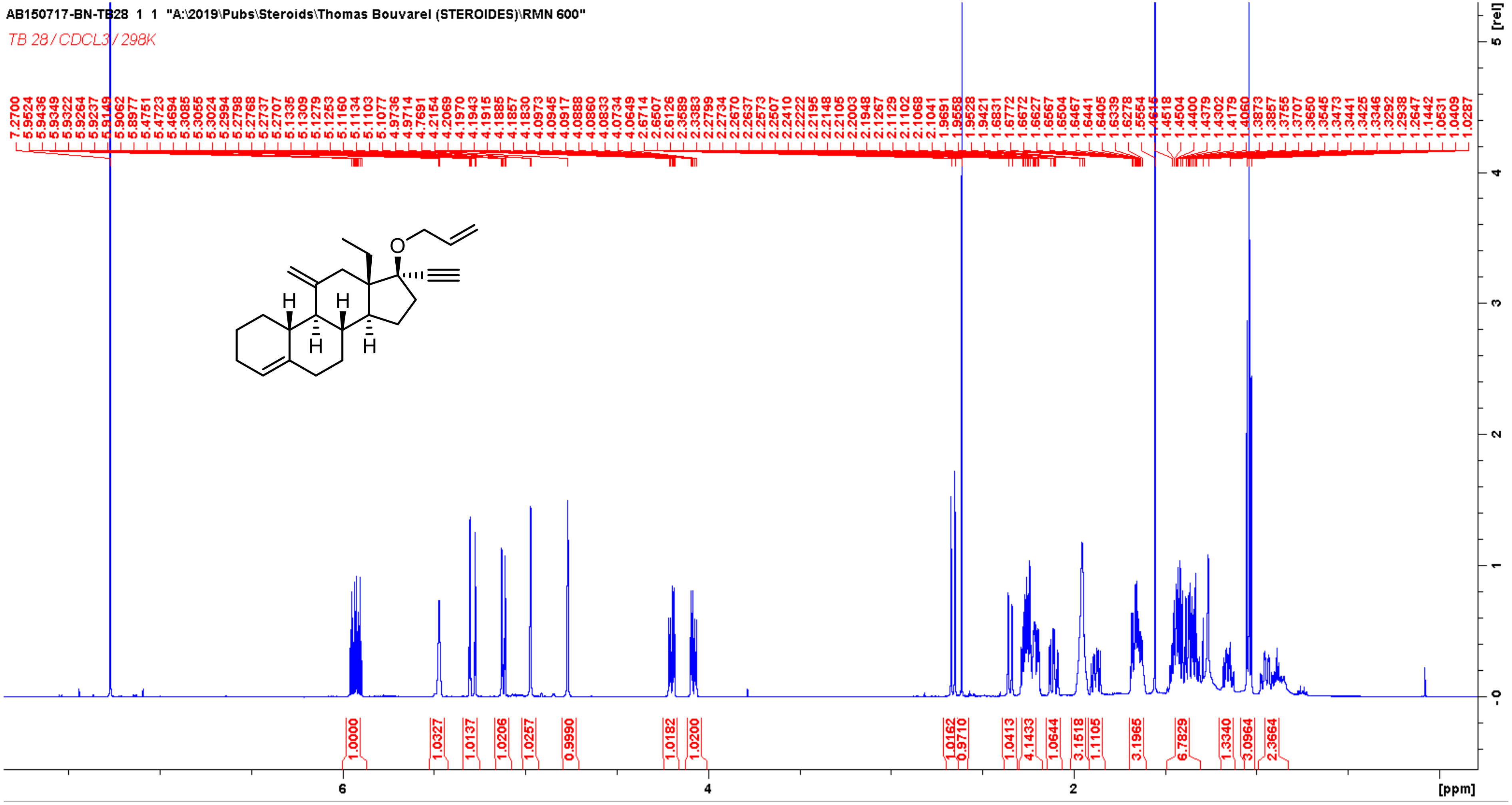

17-*O*-Allyldesogestrel (7): ^{13}C NMR (150 MHz, $CDCl_3$)

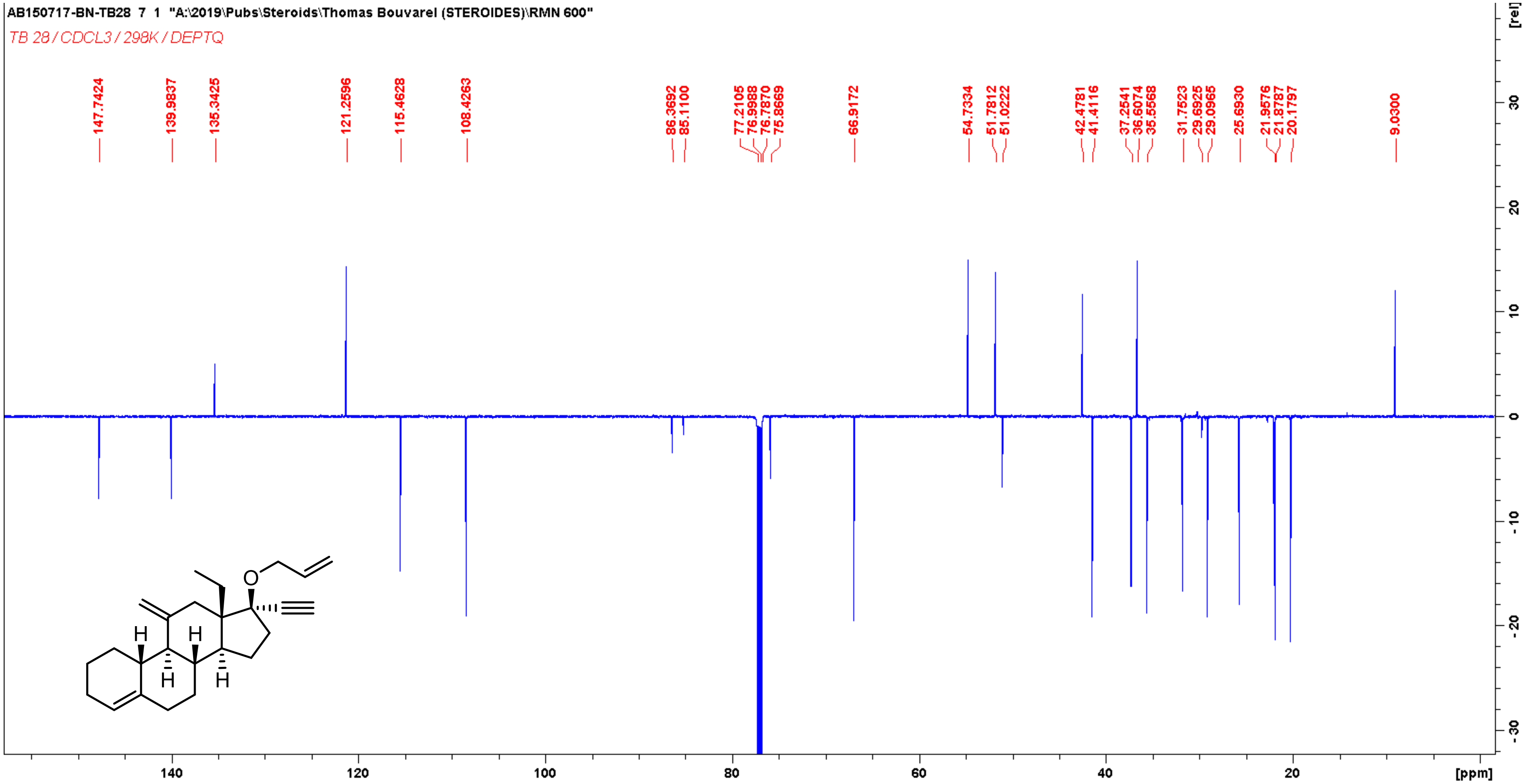

(2'*S*,8*R*,9*S*,13*S*,14*S*)-3-Methoxy-13-methyl-3'-vinyl-6,7,8,9,11,12,13,14,15,16-decahydro-5'*H*-spiro[cyclopenta[*a*]phenanthrene-17,2'-furan] (8a): ^{1}H NMR (600 MHz, CDCl$_3$)

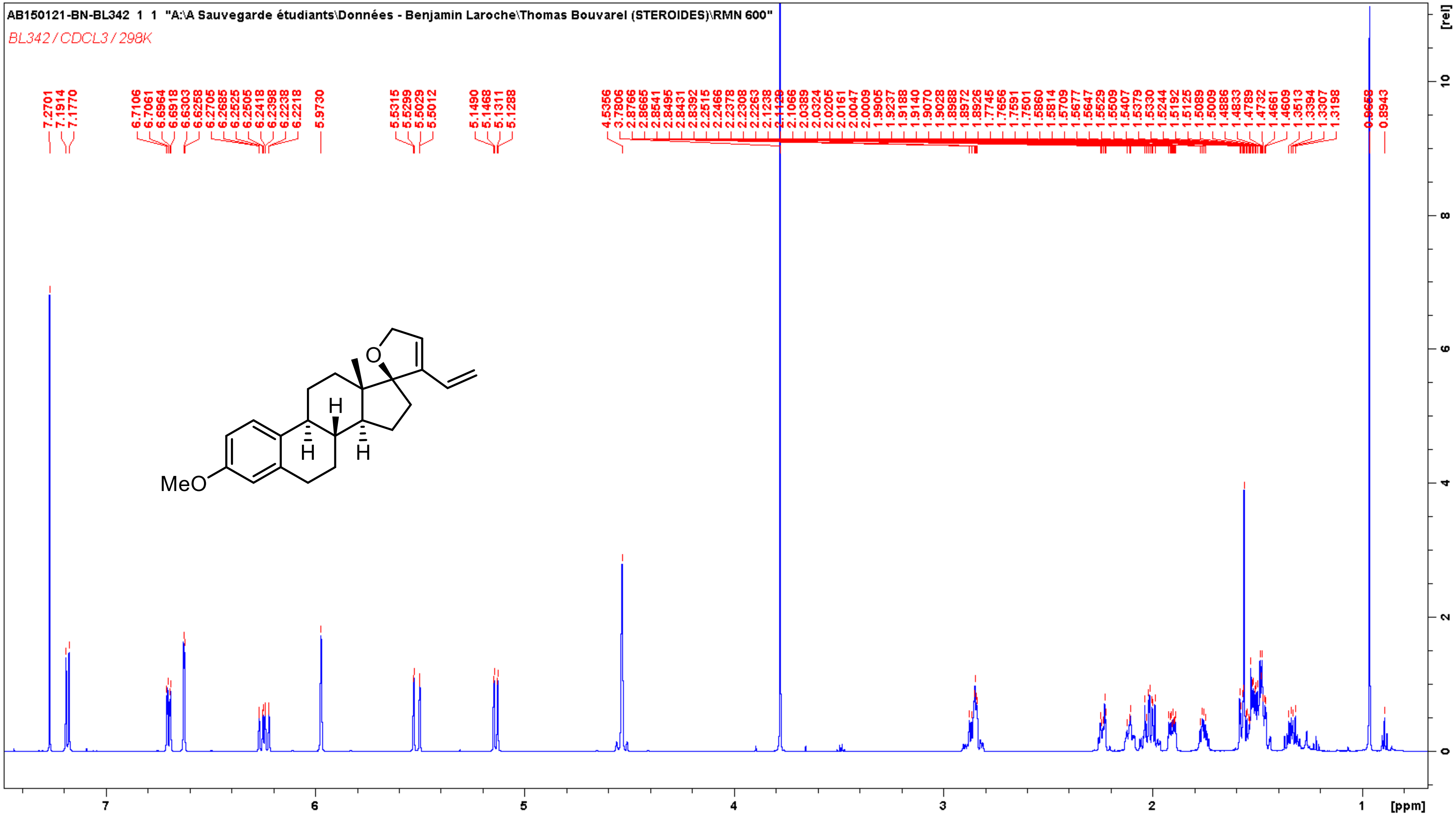

(2'*S*,8*R*,9*S*,13*S*,14*S*)-3-Methoxy-13-methyl-3'-vinyl-6,7,8,9,11,12,13,14,15,16-decahydro-5'*H*-spiro[cyclopenta[*a*]phenanthrene-17,2'-furan](8a): ^{13}C NMR (150 MHz, $CDCl_3$)

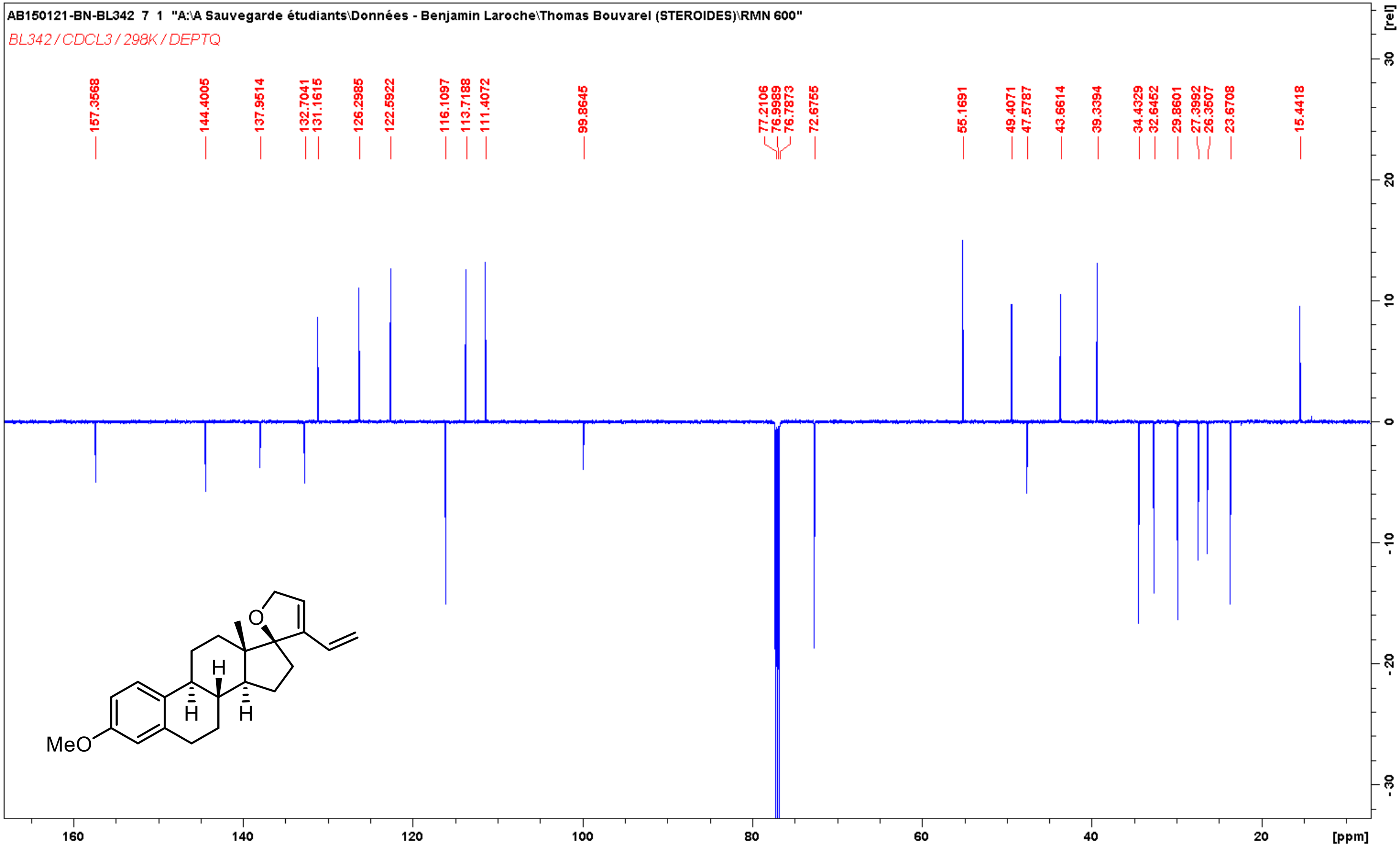

(2'*S*,8*R*,9*S*,13*S*,14*S*)-3-Methoxy-13-methyl-3'-vinyl-6,7,8,9,11,12,13,14,15,16-decahydro-5'*H*-spiro[cyclopenta[*a*]phenanthrene-17,2'-furan] (8a): COSY NMR (600 MHz, $CDCl_3$)

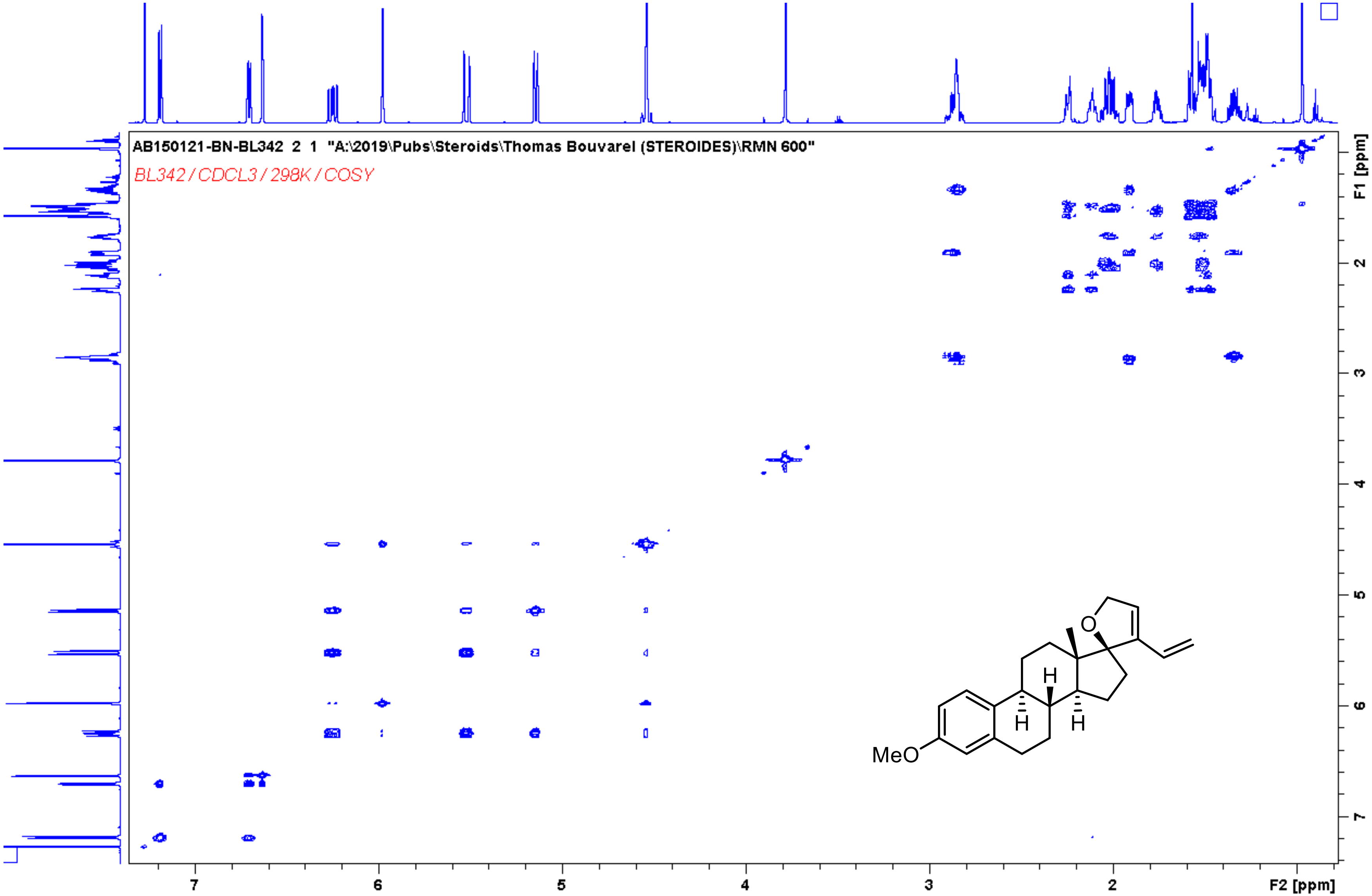

(2'*S*,8*R*,9S,13*S*,14*S*)-3-Methoxy-13-methyl-3'-vinyl-6,7,8,9,11,12,13,14,15,16-decahydro-5'*H*-spiro[cyclopenta[*a*]phenanthrene-17,2'-furan] (8a): HMBC NMR (600 MHz, $CDCl_3$)

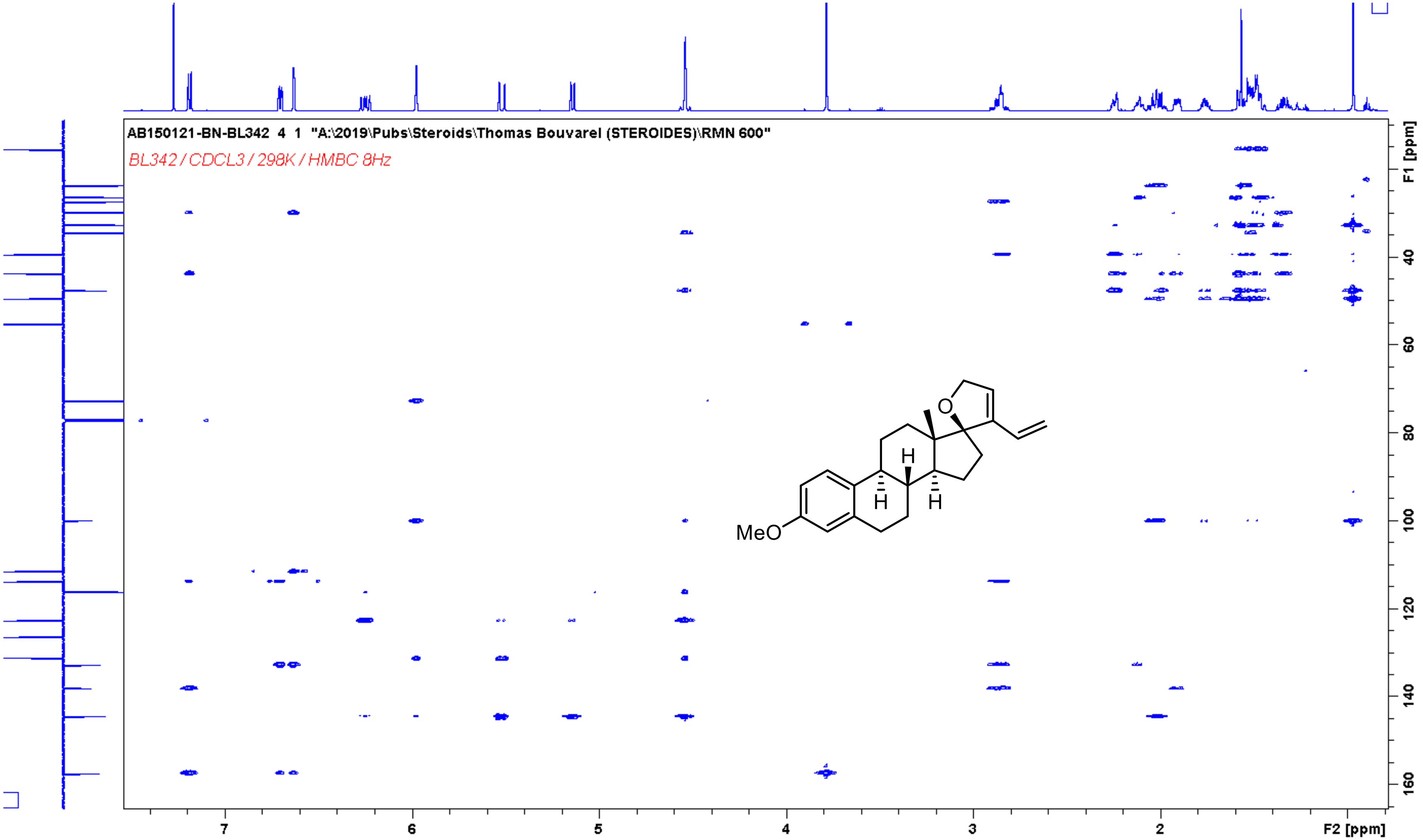

(2'*S*,8*R*,9*S*,13*S*,14*S*)-3-Methoxy-13-methyl-3'-vinyl-6,7,8,9,11,12,13,14,15,16-decahydro-5'*H*-spiro[cyclopenta[*a*]phenanthrene-17,2'-furan] (8a): HSQC NMR (600 MHz, $CDCl_3$)

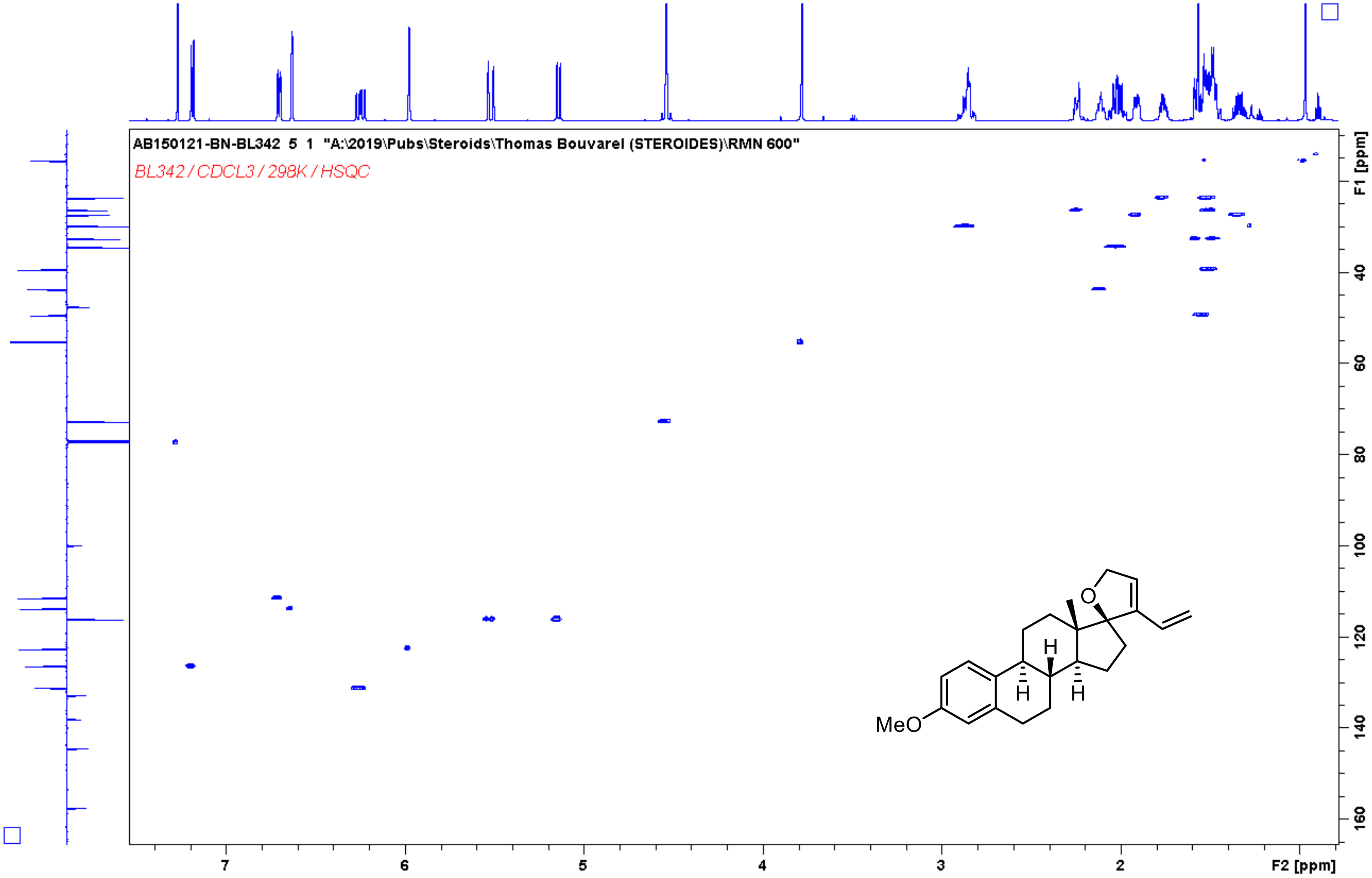

(2'*S*,8*R*,9*S*,13*S*,14*S*)-3-Methoxy-13-methyl-3'-vinyl-6,7,8,9,11,12,13,14,15,16-decahydro-5'*H*-spiro[cyclopenta[*a*]phenanthrene-17,2'-furan] (8a): NOESY NMR (600 MHz, $CDCl_3$)

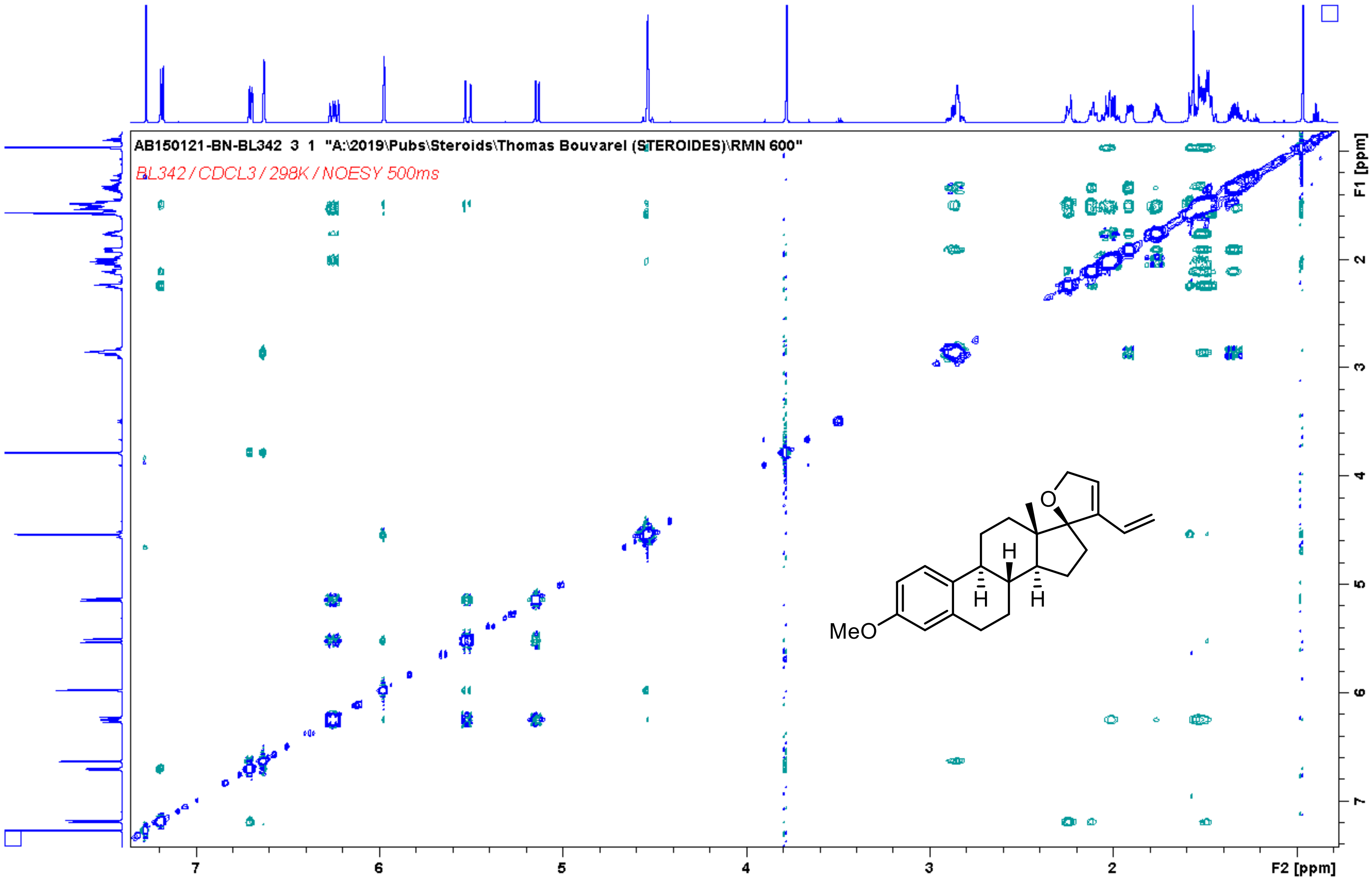

(2'*S*,8*R*,9*S*,13*S*,14*S*)-3-Methoxy-13-methyl-3'-vinyl-6,6',7,7',8,9,11,12,13,14,15,16-dodecahydro-5'*H*-spiro[cyclopenta[*a*]phenanthrene-17,2'-oxepine](8b): ^{1}H NMR (600 MHz, CDCl$_3$)

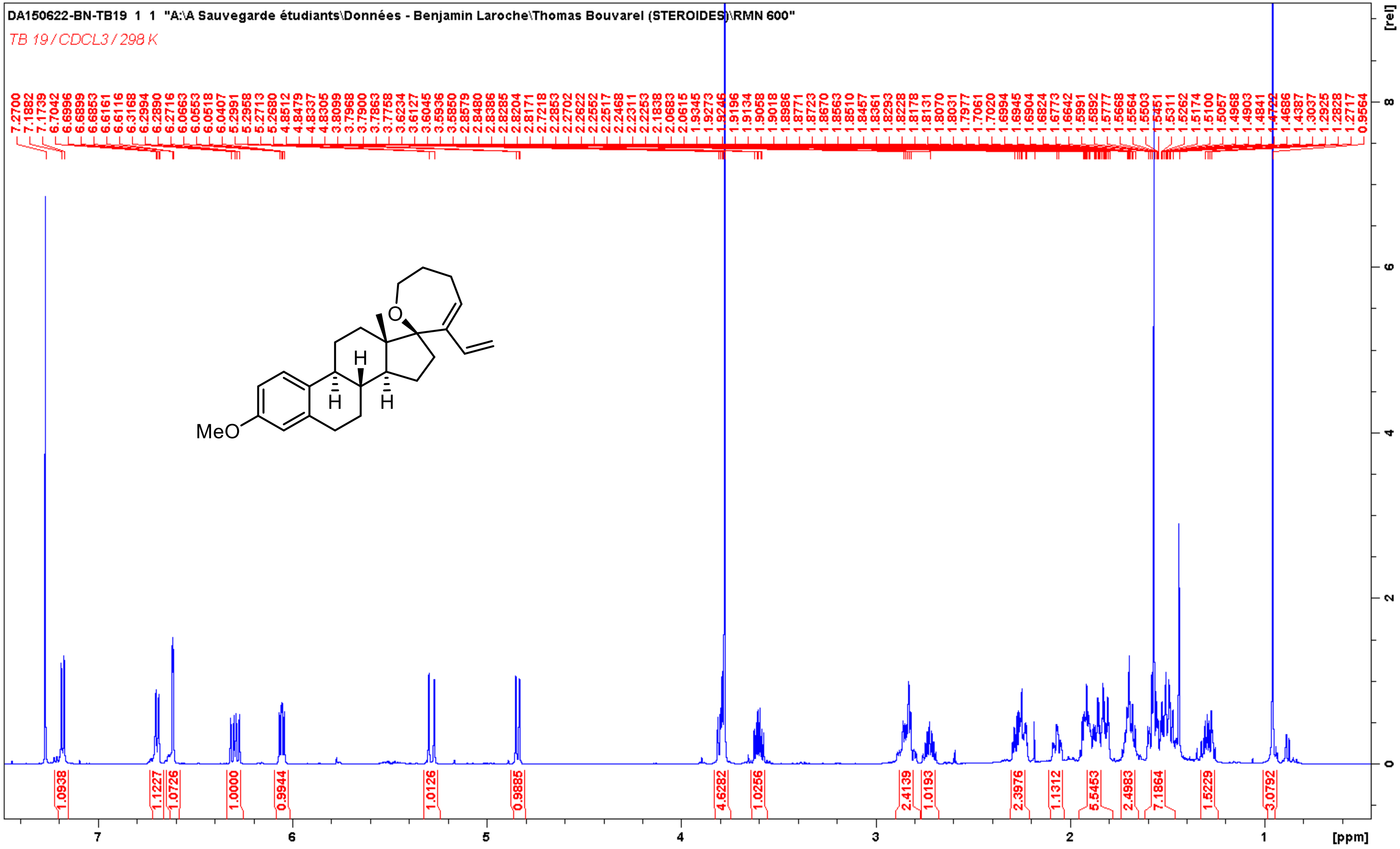

(2'*S*,8*R*,9*S*,13*S*,14*S*)-3-Methoxy-13-methyl-3'-vinyl-6,6',7,7',8,9,11,12,13,14,15,16-dodecahydro-5'*H*-spiro[cyclopenta[*a*]phenanthrene-17,2'-oxepine] (8b): ^{13}C NMR (150 MHz, CDCl$_3$)

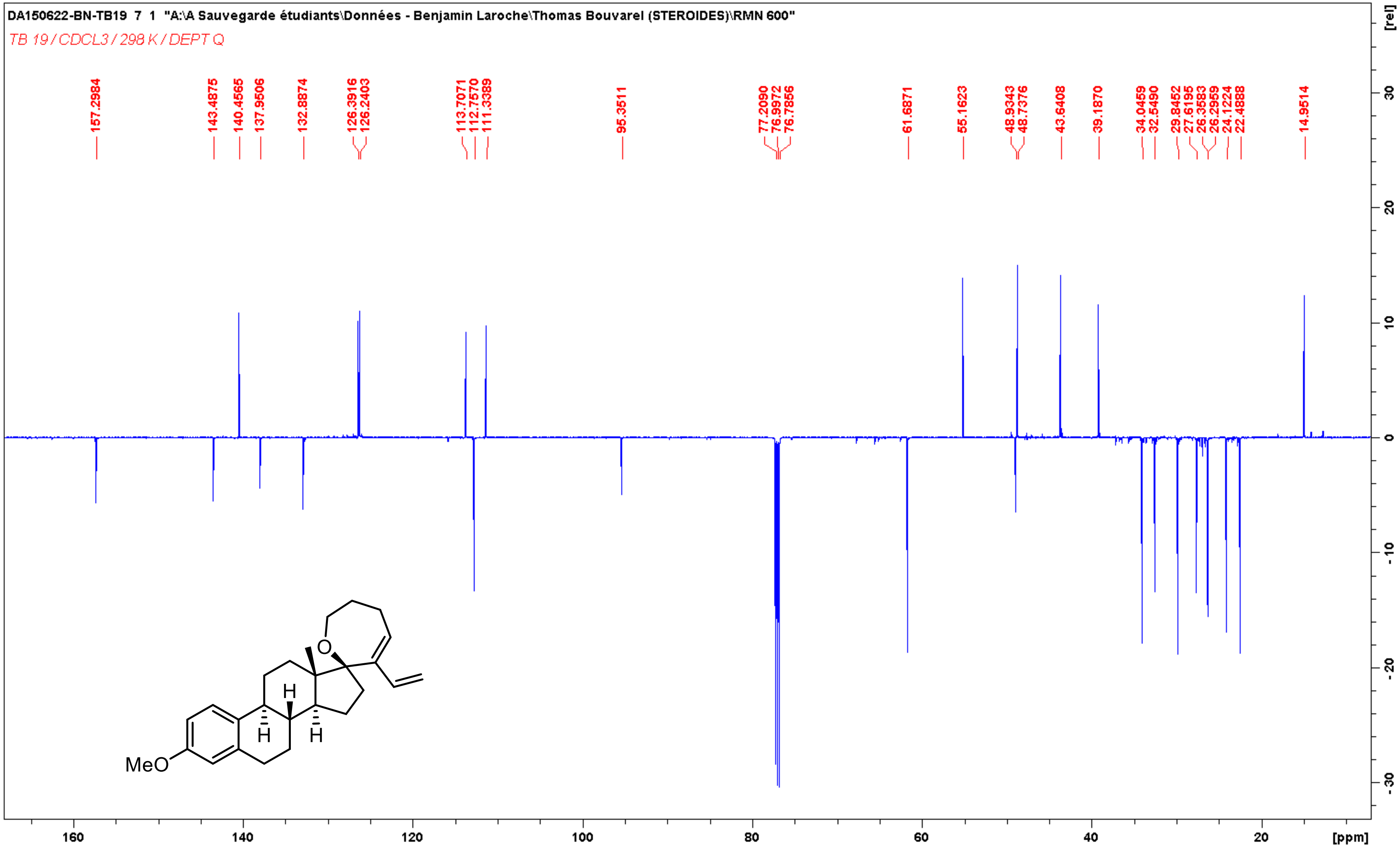

(2'*S*,8*R*,9*S*,13*S*,14*S*)-3-Methoxy-13-methyl-3'-vinyl-6,6',7,7',8,9,11,12,13,14,15,16-dodecahydro-5'*H*-spiro[cyclopenta[*a*]phenanthrene-17,2'-oxepine](8b): COSY NMR (600 MHz, $CDCl_3$)

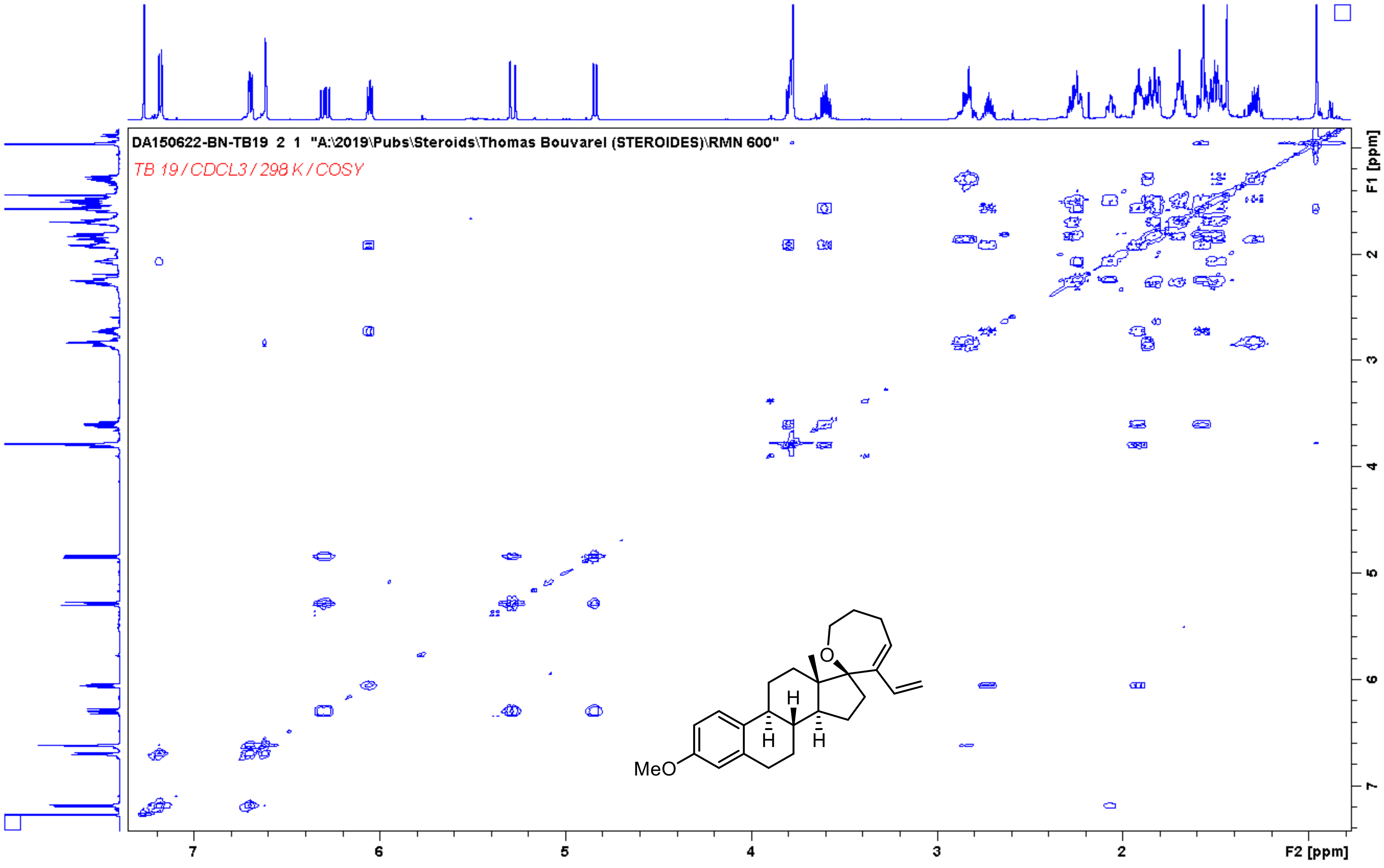

(2'*S*,8*R*,9*S*,13*S*,14*S*)-3-Methoxy-13-methyl-3'-vinyl-6,6',7,7',8,9,11,12,13,14,15,16-dodecahydro-5'*H*-spiro[cyclopenta[*a*]phenanthrene-17,2'-oxepine](8b): HMBC NMR (600 MHz, $CDCl_3$)

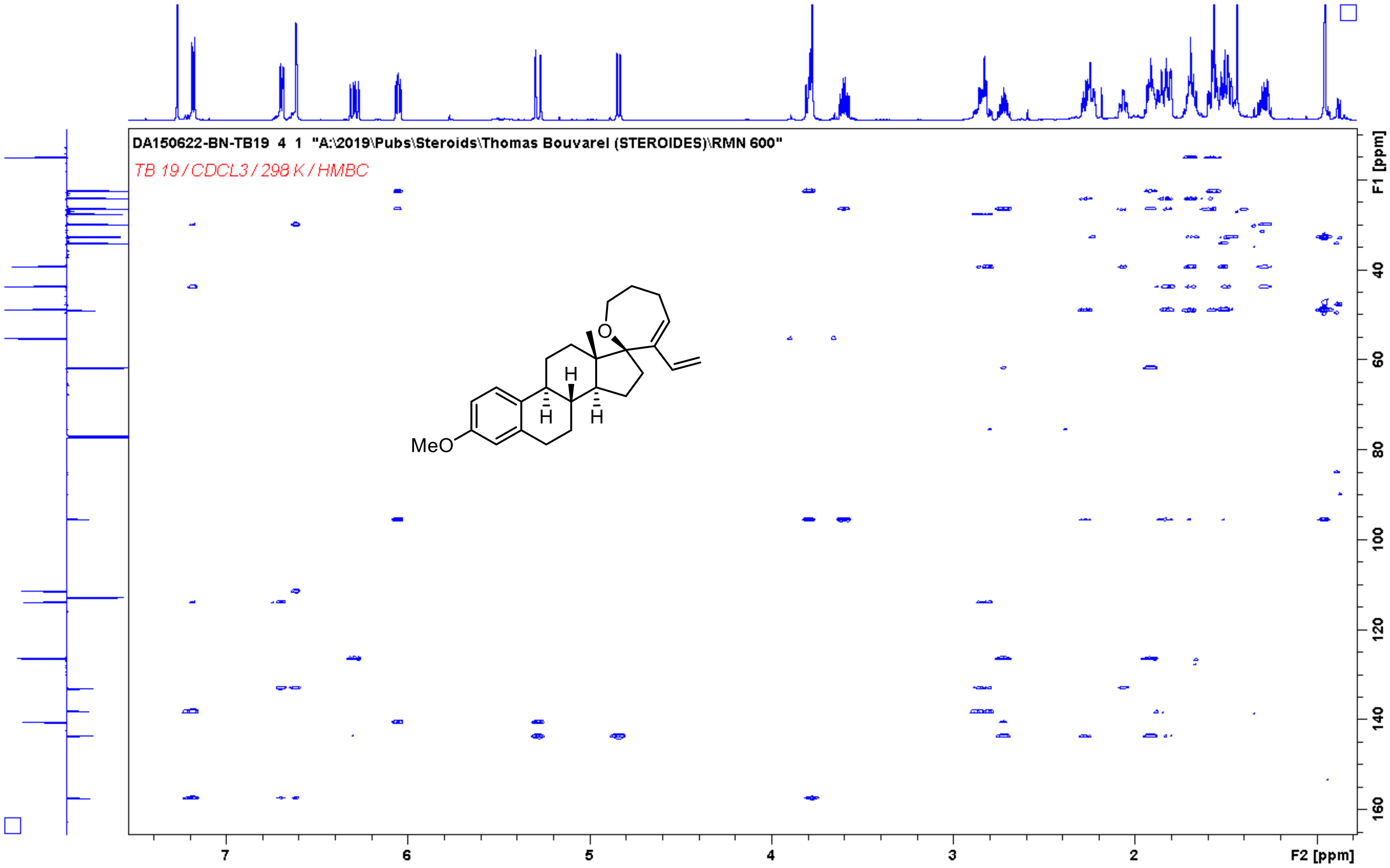

(2'*S*,8*R*,9*S*,13*S*,14*S*)-3-Methoxy-13-methyl-3'-vinyl-6,6',7,7',8,9,11,12,13,14,15,16-dodecahydro-5'*H*-spiro[cyclopenta[*a*]phenanthrene-17,2'-oxepine](8b): HSQC NMR (600 MHz, $CDCl_3$)

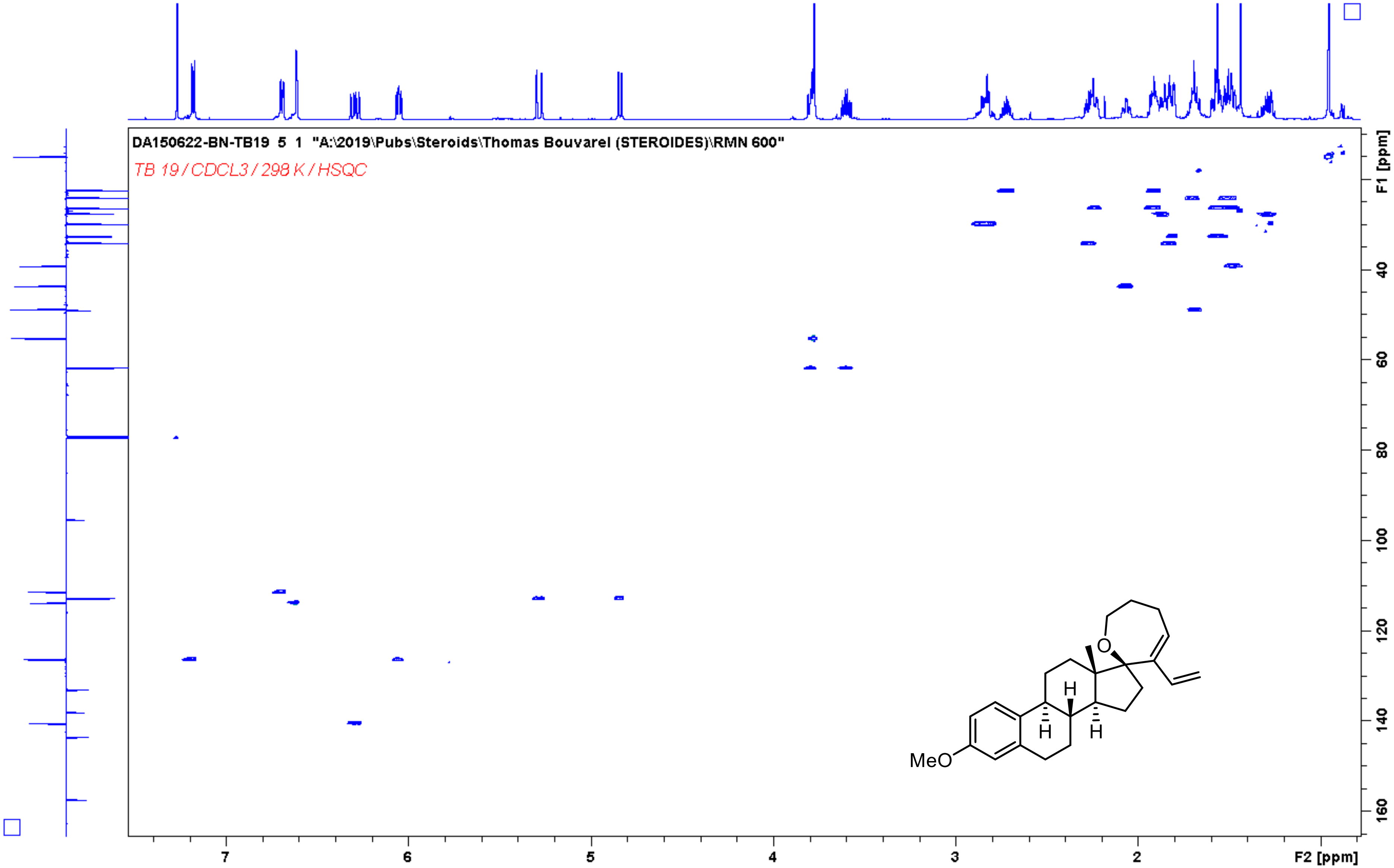

(2'*S*,8*R*,9*S*,13*S*,14*S*)-3-Methoxy-13-methyl-3'-vinyl-6,6',7,7',8,9,11,12,13,14,15,16-dodecahydro-5'*H*-spiro[cyclopenta[*a*]phenanthrene-17,2'-oxepine](8b): NOESY NMR (600 MHz, CDCl$_3$)

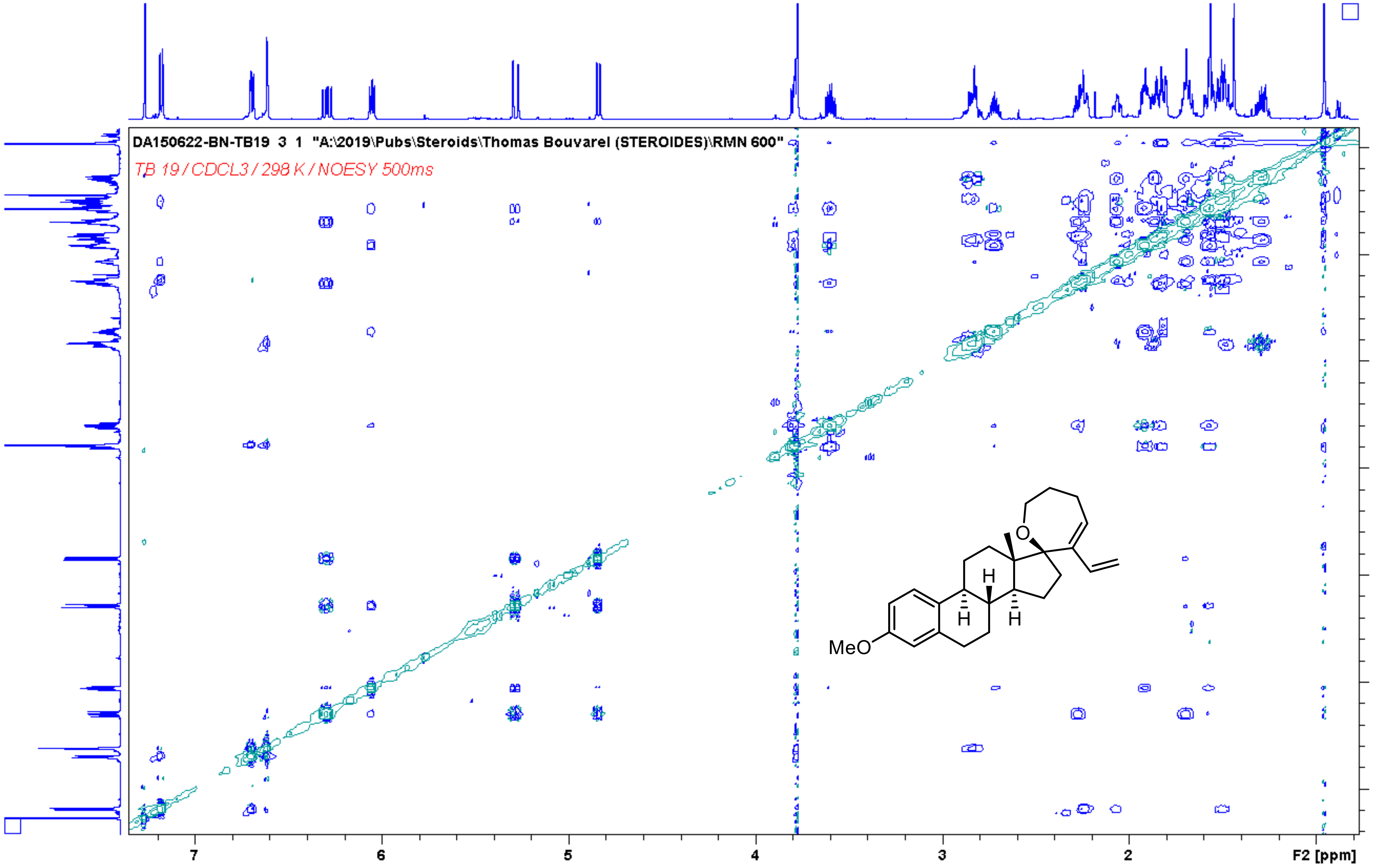

(2'*S*,8*R*,9*S*,10*R*,13*S*,14*S*)-13-Methyl-3'-vinyl-1,2,3,6,7,8,9,10,11,12,13,14,15,16-tetradecahydro-5'*H*-spiro[cyclopenta[*a*]phenanthrene-17,2'-furan] (9a): ^{1}H NMR (600 MHz, $CDCl_3$)

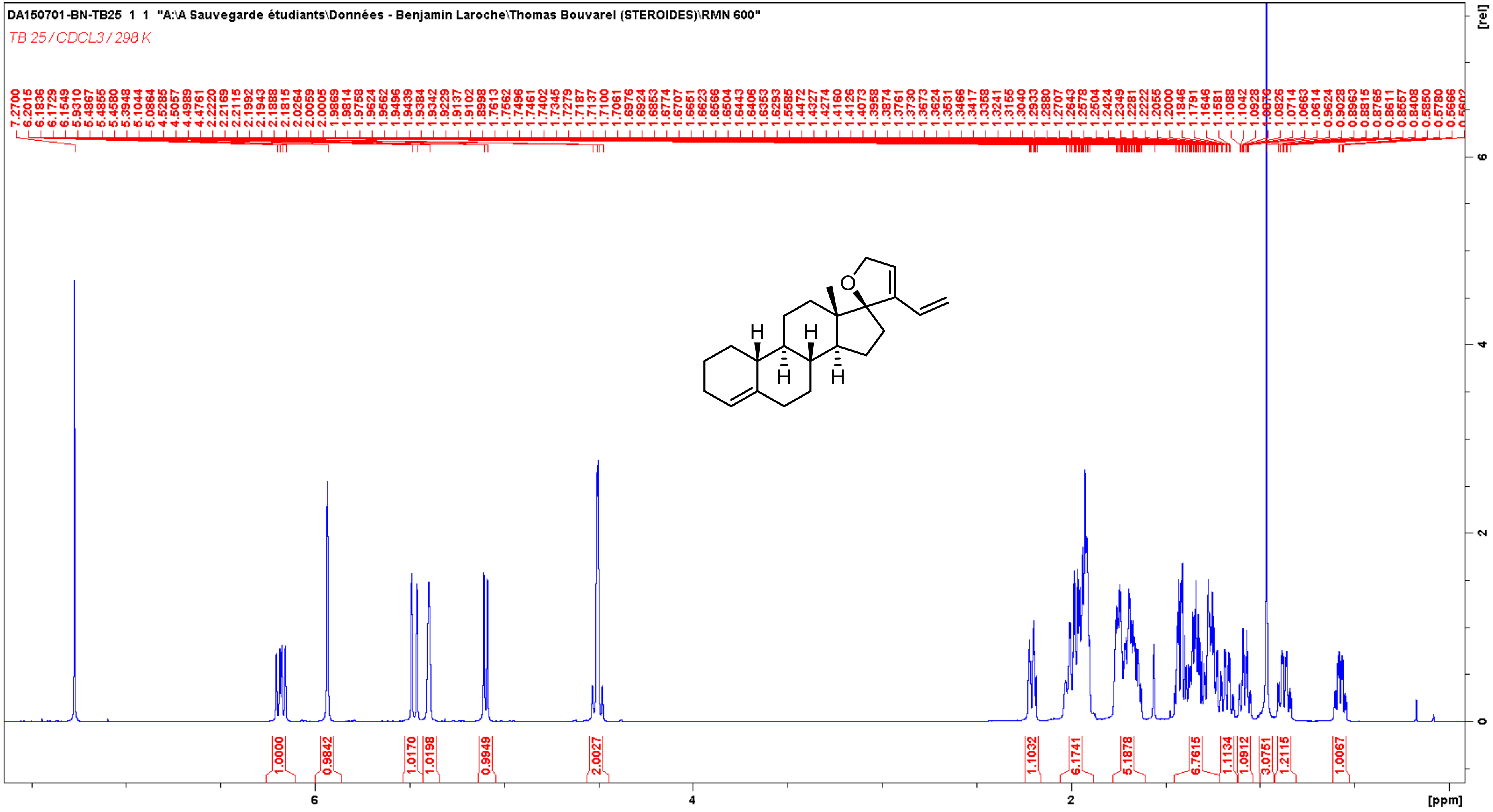

(2'*S*,8*R*,9*S*,10*R*,13*S*,14*S*)-13-Methyl-3'-vinyl-1,2,3,6,7,8,9,10,11,12,13,14,15,16-tetradecahydro-5'*H*-spiro[cyclopenta[*a*]phenanthrene-17,2'-furan] (9a): ^{13}C NMR (150 MHz, CDCl$_3$)

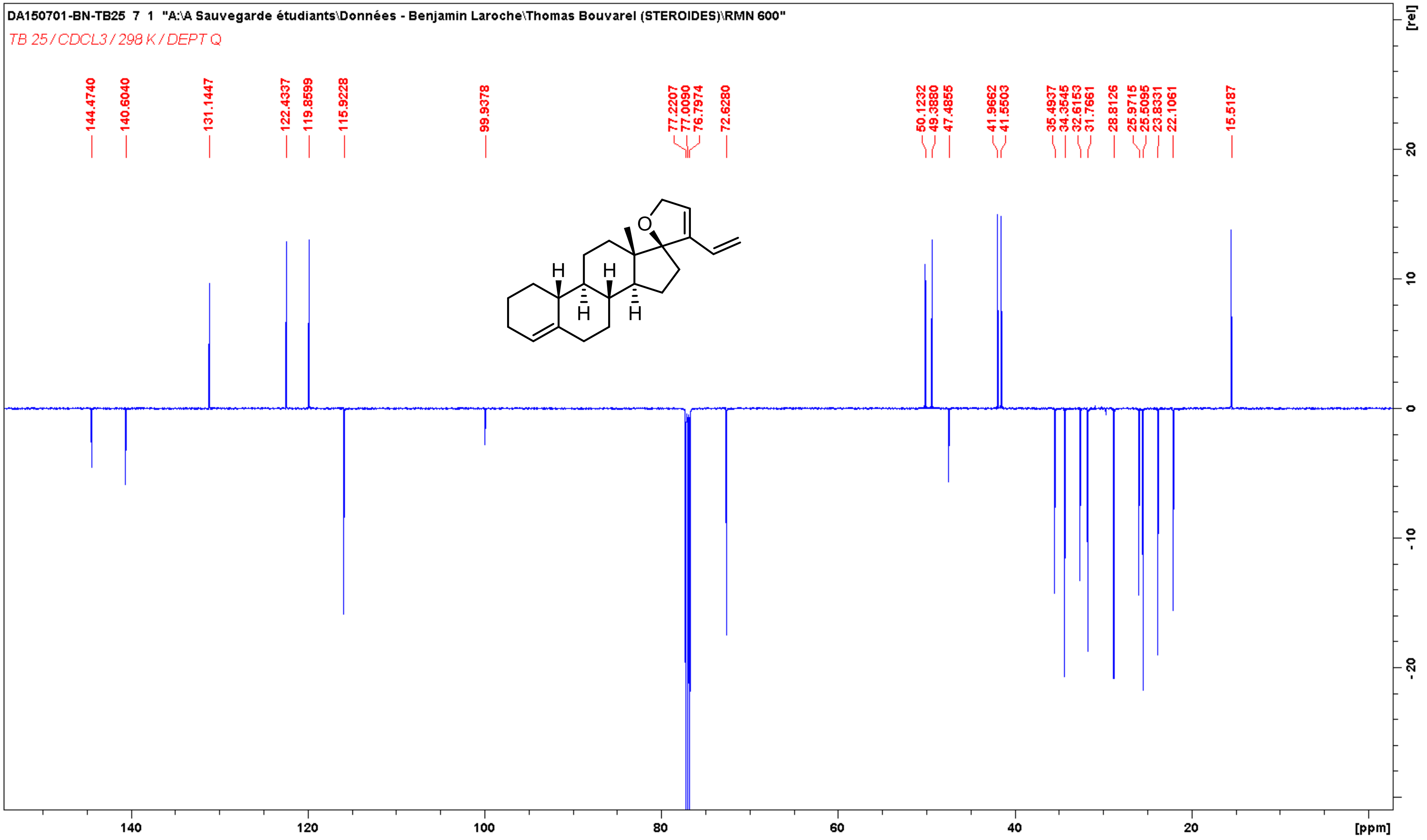

(2'*S*,8*R*,9*S*,10*R*,13*S*,14*S*)-13-Methyl-3'-vinyl-1,2,3,6,7,8,9,10,11,12,13,14,15,16-tetradecahydro-5'*H*-spiro[cyclopenta[*a*]phenanthrene-17,2'-furan] (9a): COSY NMR (600 MHz, $CDCl_3$)

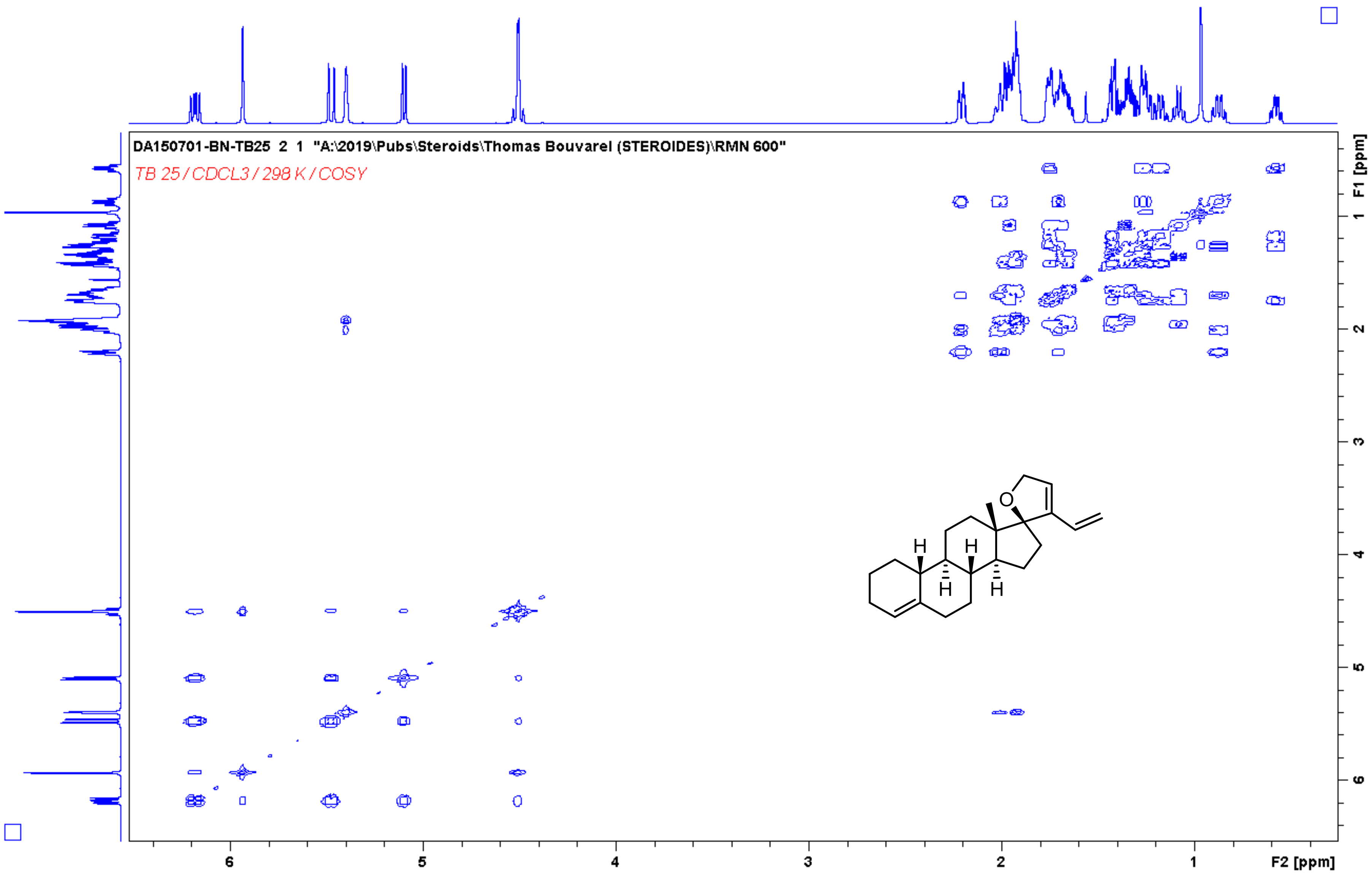

(2'*S*,8*R*,9*S*,10*R*,13*S*,14*S*)-13-Methyl-3'-vinyl-1,2,3,6,7,8,9,10,11,12,13,14,15,16-tetradecahydro-5'*H*-spiro[cyclopenta[*a*]phenanthrene-17,2'-furan] (9a): HMBC NMR (600 MHz, $CDCl_3$)

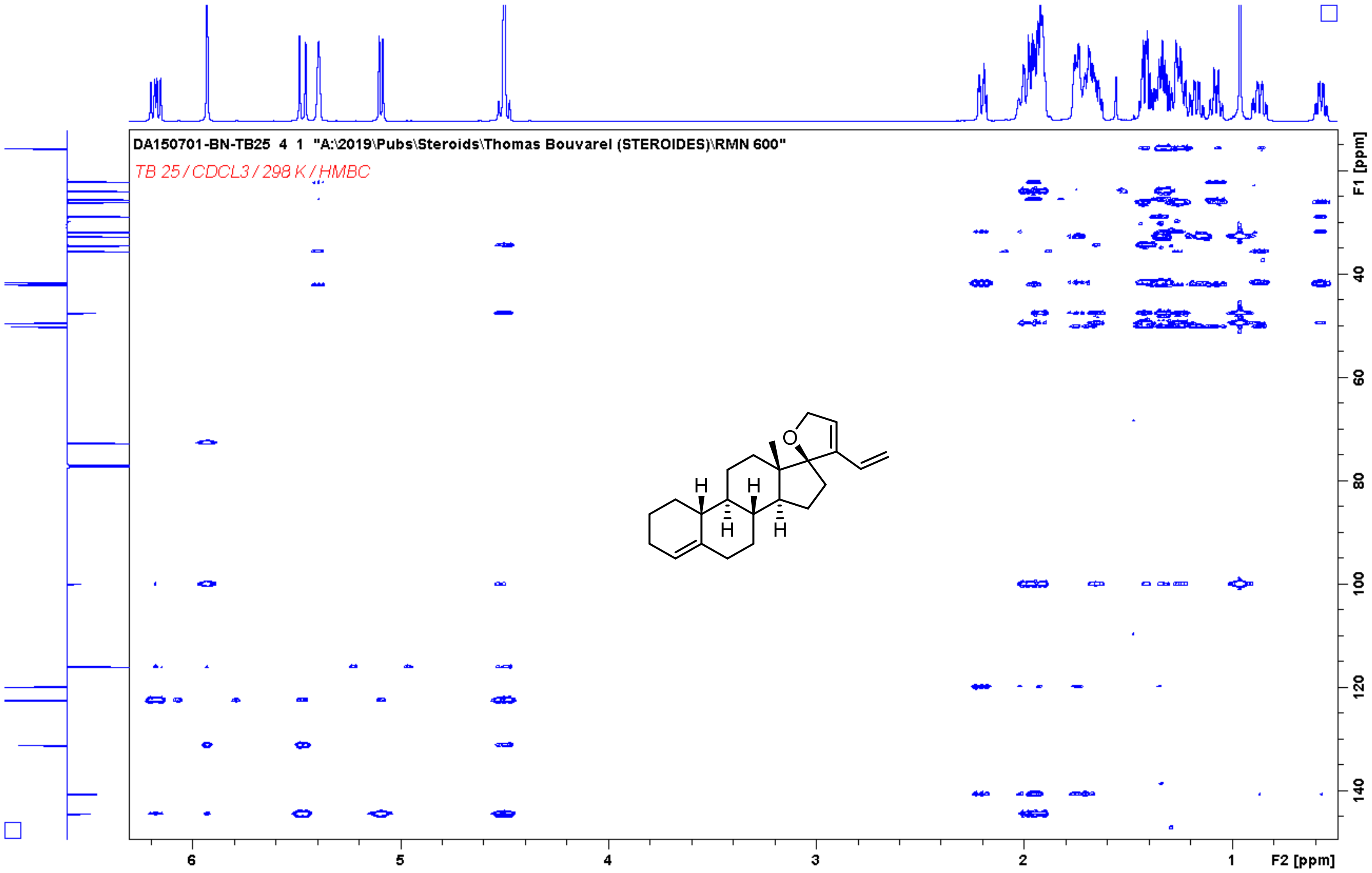

(2'*S*,8*R*,9*S*,10*R*,13*S*,14*S*)-13-Methyl-3'-vinyl-1,2,3,6,7,8,9,10,11,12,13,14,15,16-tetradecahydro-5'*H*-spiro[cyclopenta[*a*]phenanthrene-17,2'-furan] (9a): HSQC NMR (600 MHz, $CDCl_3$)

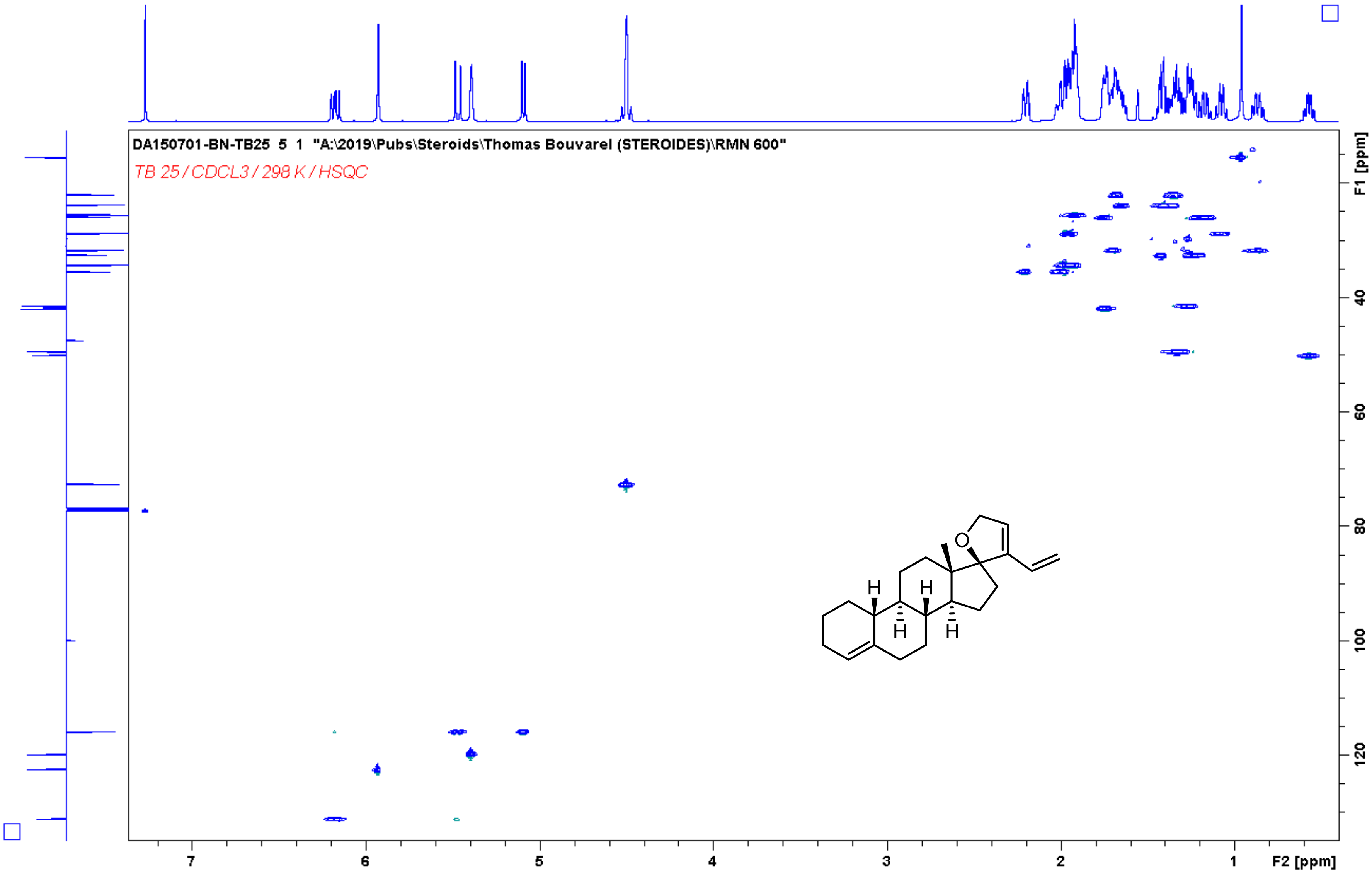

(2'*S*,8*R*,9*S*,10*R*,13*S*,14*S*)-13-Methyl-3'-vinyl-1,2,3,6,7,8,9,10,11,12,13,14,15,16-tetradecahydro-5'*H*-spiro[cyclopenta[*a*]phenanthrene-17,2'-furan] (9a): NOESY NMR (600 MHz, $CDCl_3$)

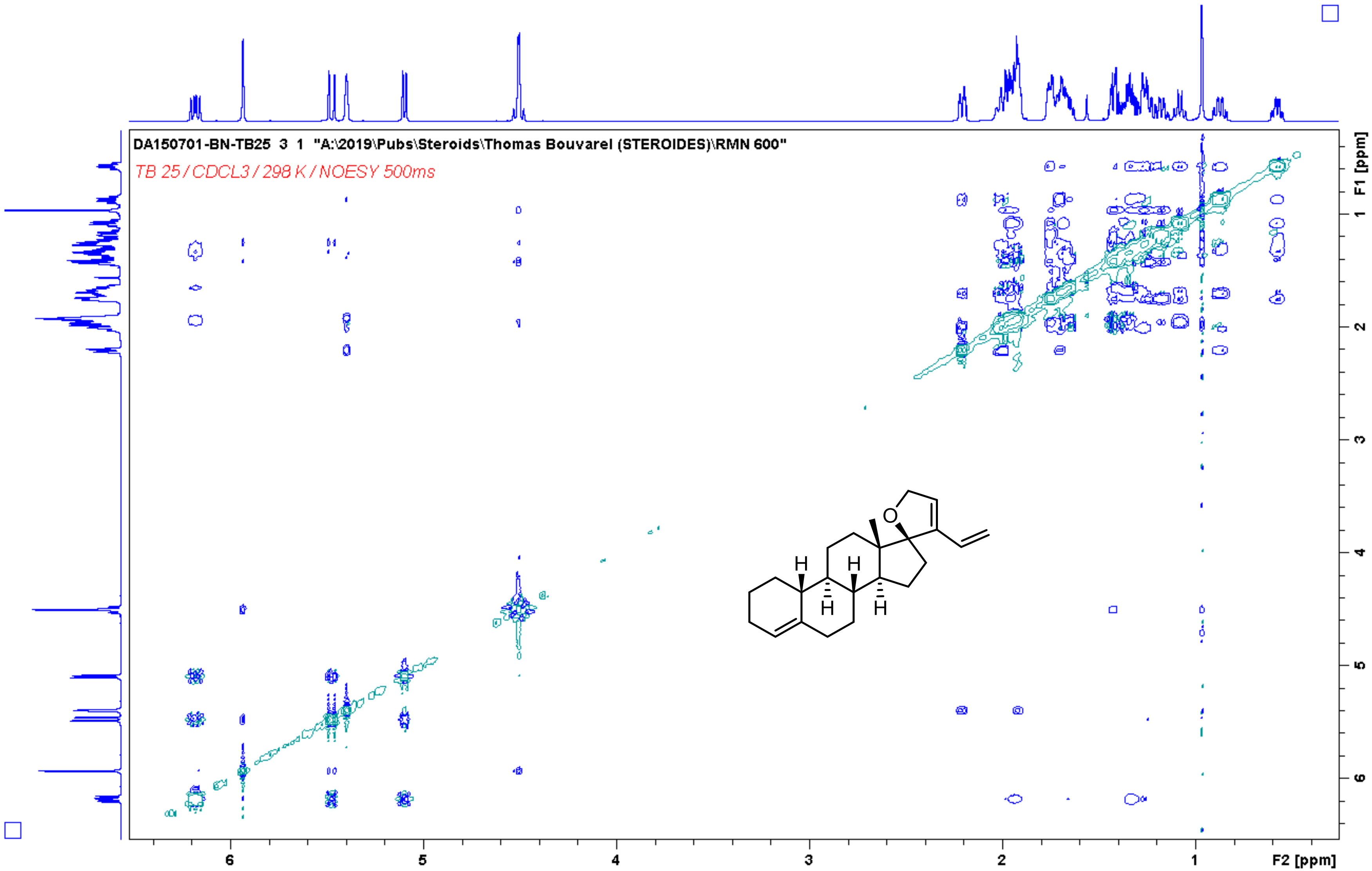

(2'*S*,8*R*,9*S*,10*R*,13*S*,14*S*)-13-Methyl-3'-vinyl-1,2,3,6,6',7,7',8,9,10,11,12,13,14,15,16-hexadecahydro-5'*H*-spiro[cyclopenta[*a*]phenanthrene-17,2'-oxepine] (9b): ^{1}H NMR (600 MHz, $CDCl_3$)

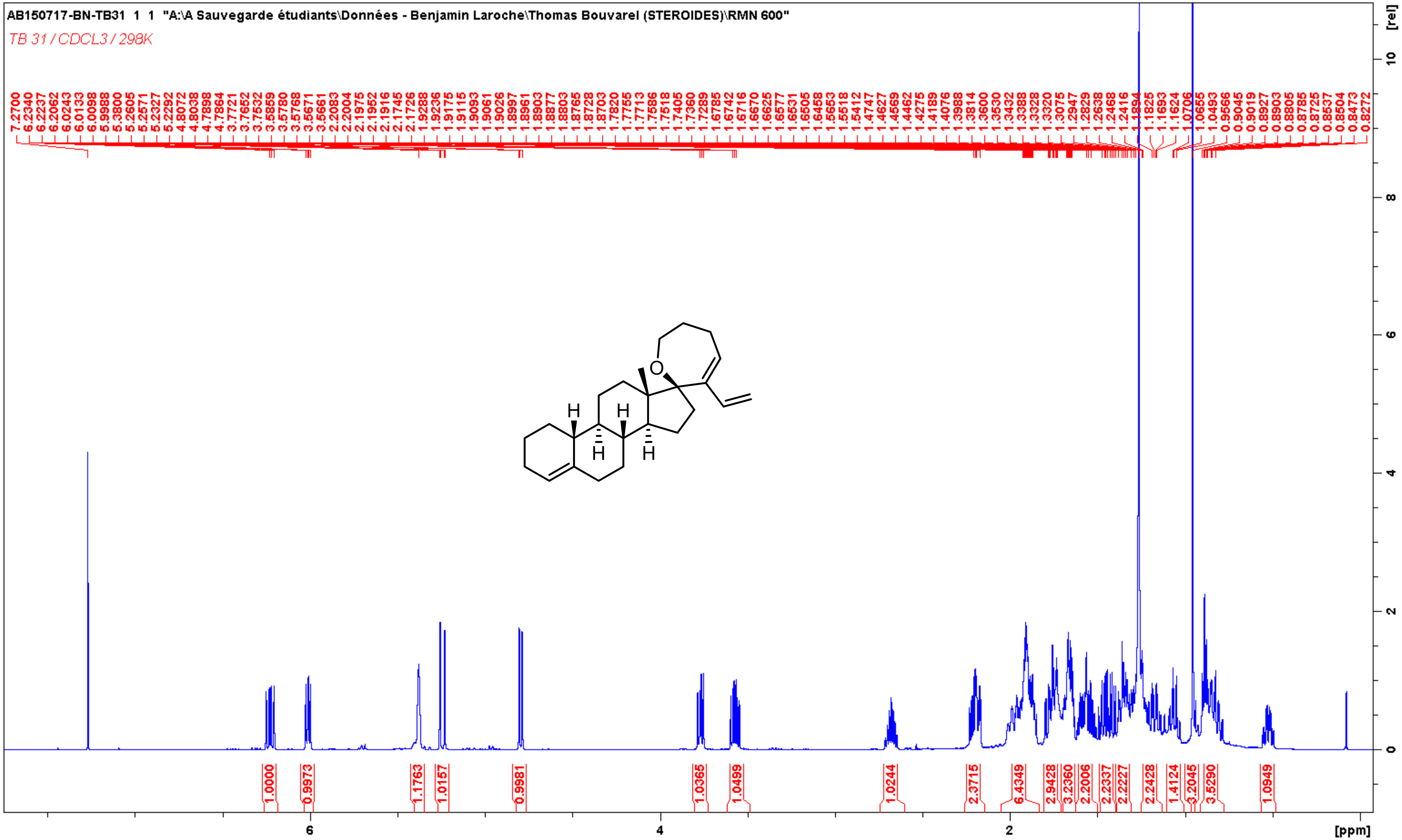

(2'*S*,8*R*,9*S*,10*R*,13*S*,14*S*)-13-Methyl-3'-vinyl-1,2,3,6,6',7,7',8,9,10,11,12,13,14,15,16-hexadecahydro-5'*H*-spiro[cyclopenta[*a*]phenanthrene-17,2'-oxepine] (9b): ^{13}C NMR (150 MHz, $CDCl_3$)

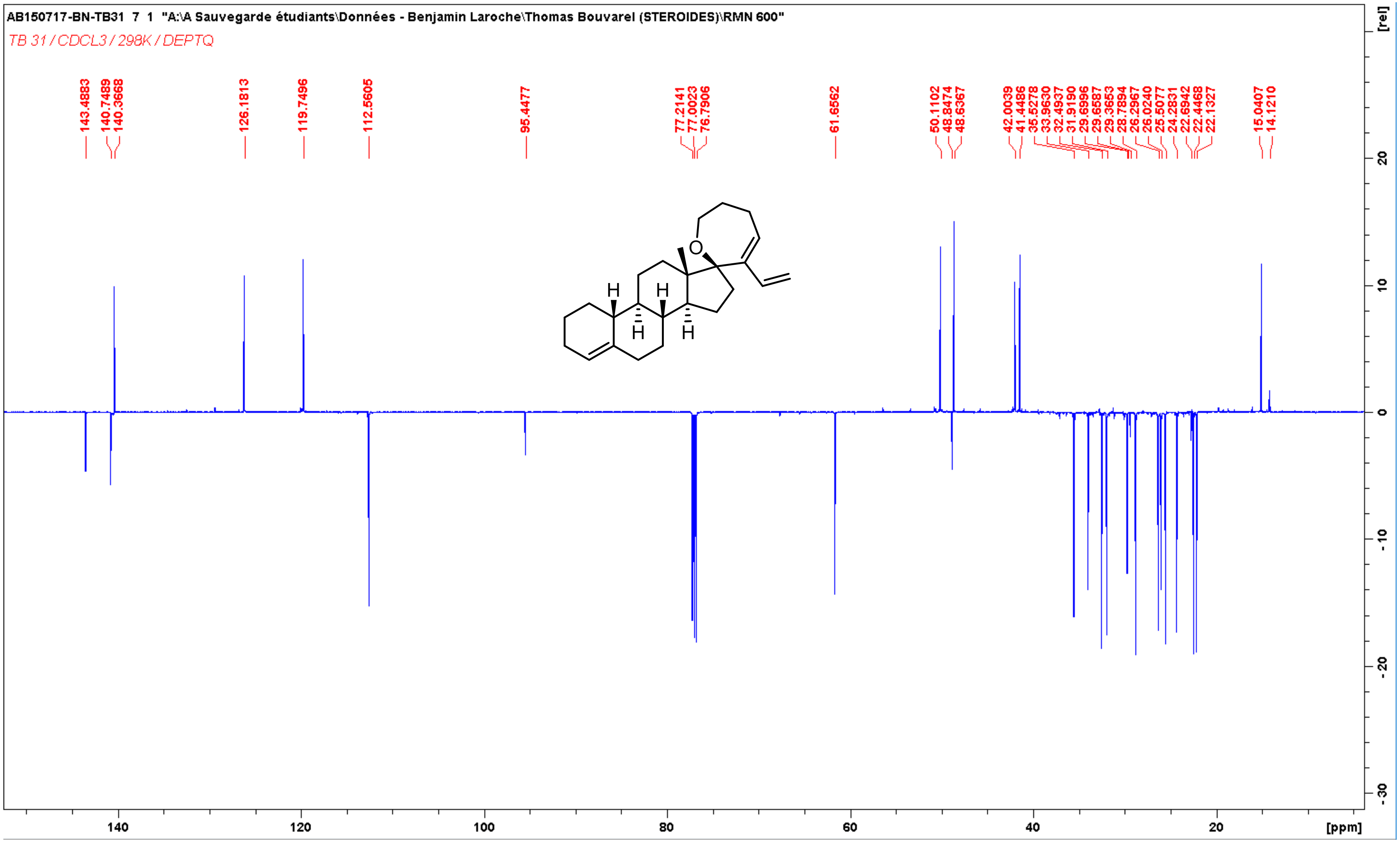

(2'*S*,8*R*,9*S*,10*R*,13*S*,14*S*)-13-Methyl-3'-vinyl-1,2,3,6,6',7,7',8,9,10,11,12,13,14,15,16-hexadecahydro-5'*H*-spiro[cyclopenta[*a*]phenanthrene-17,2'-oxepine] (9b): COSY NMR (600 MHz, $CDCl_3$)

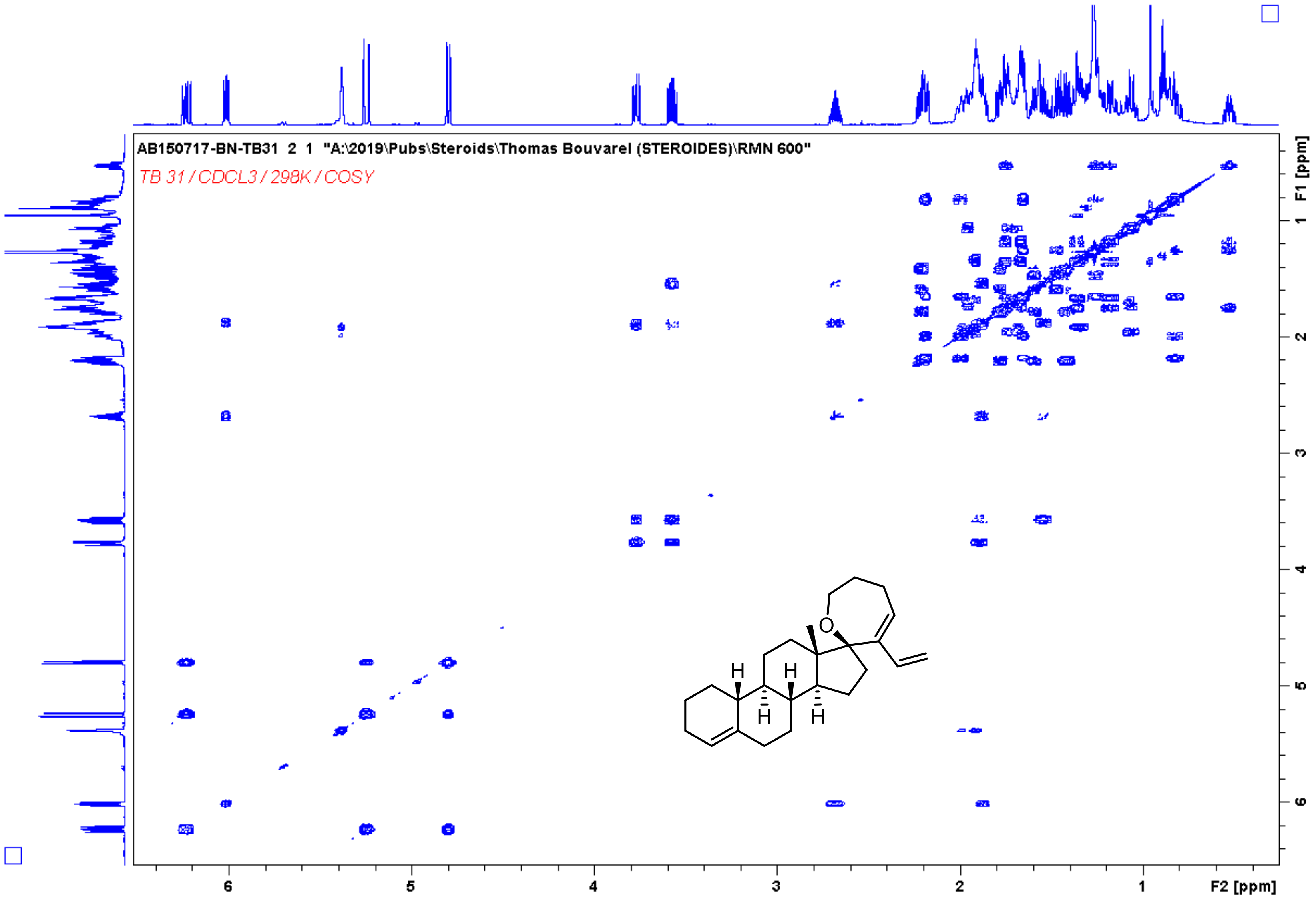

(2'*S*,8*R*,9*S*,10*R*,13*S*,14*S*)-13-Methyl-3'-vinyl-1,2,3,6,6',7,7',8,9,10,11,12,13,14,15,16-hexadecahydro-5'*H*-spiro[cyclopenta[*a*]phenanthrene-17,2'-oxepine] (9b): HMBC NMR (600 MHz, $CDCl_3$)

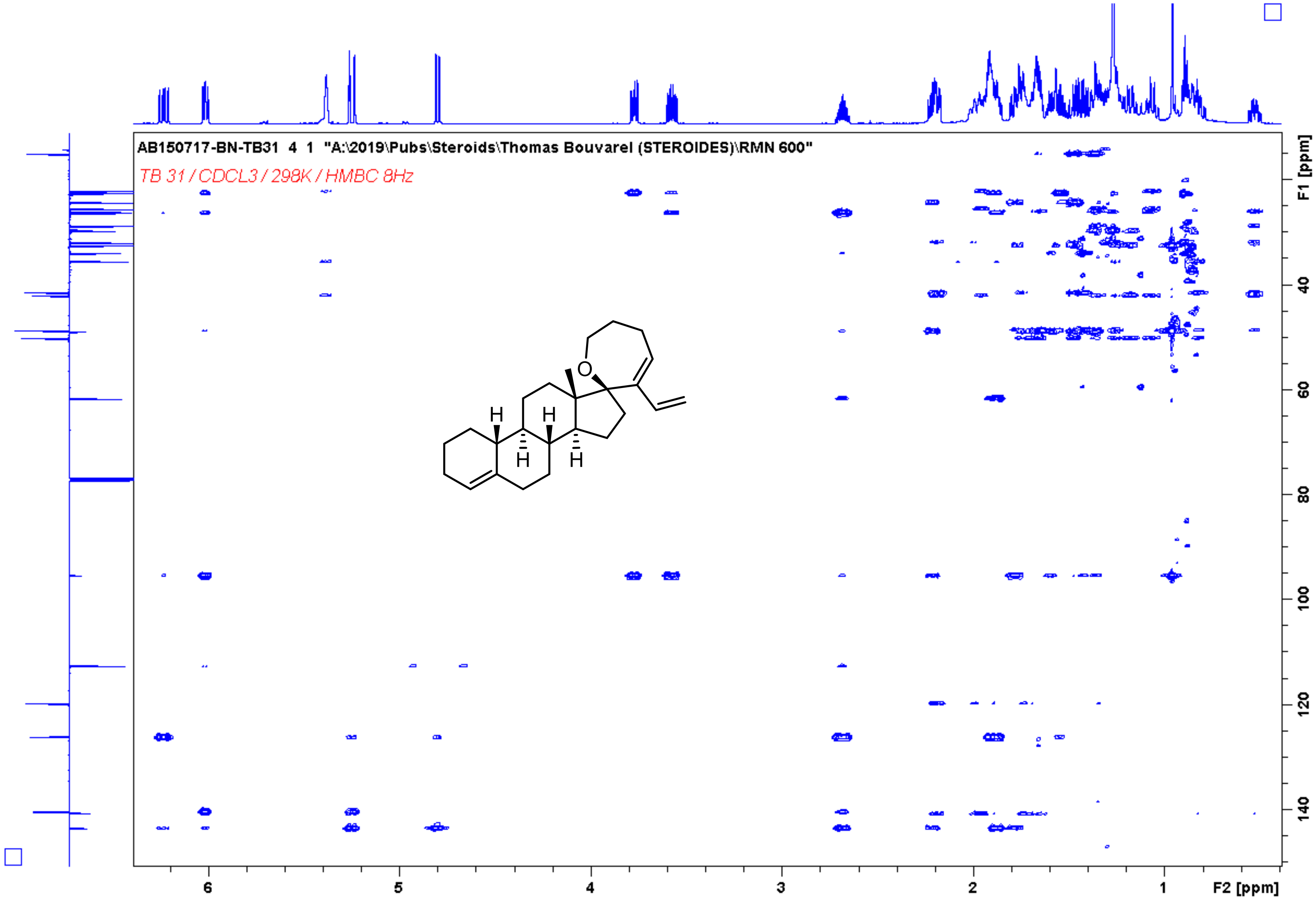

(2'*S*,8*R*,9*S*,10*R*,13*S*,14*S*)-13-Methyl-3'-vinyl-1,2,3,6,6',7,7',8,9,10,11,12,13,14,15,16-hexadecahydro-5'*H*-spiro[cyclopenta[a]phenanthrene-17,2'-oxepine] (9b): HSQC NMR (600 MHz, $CDCl_3$)

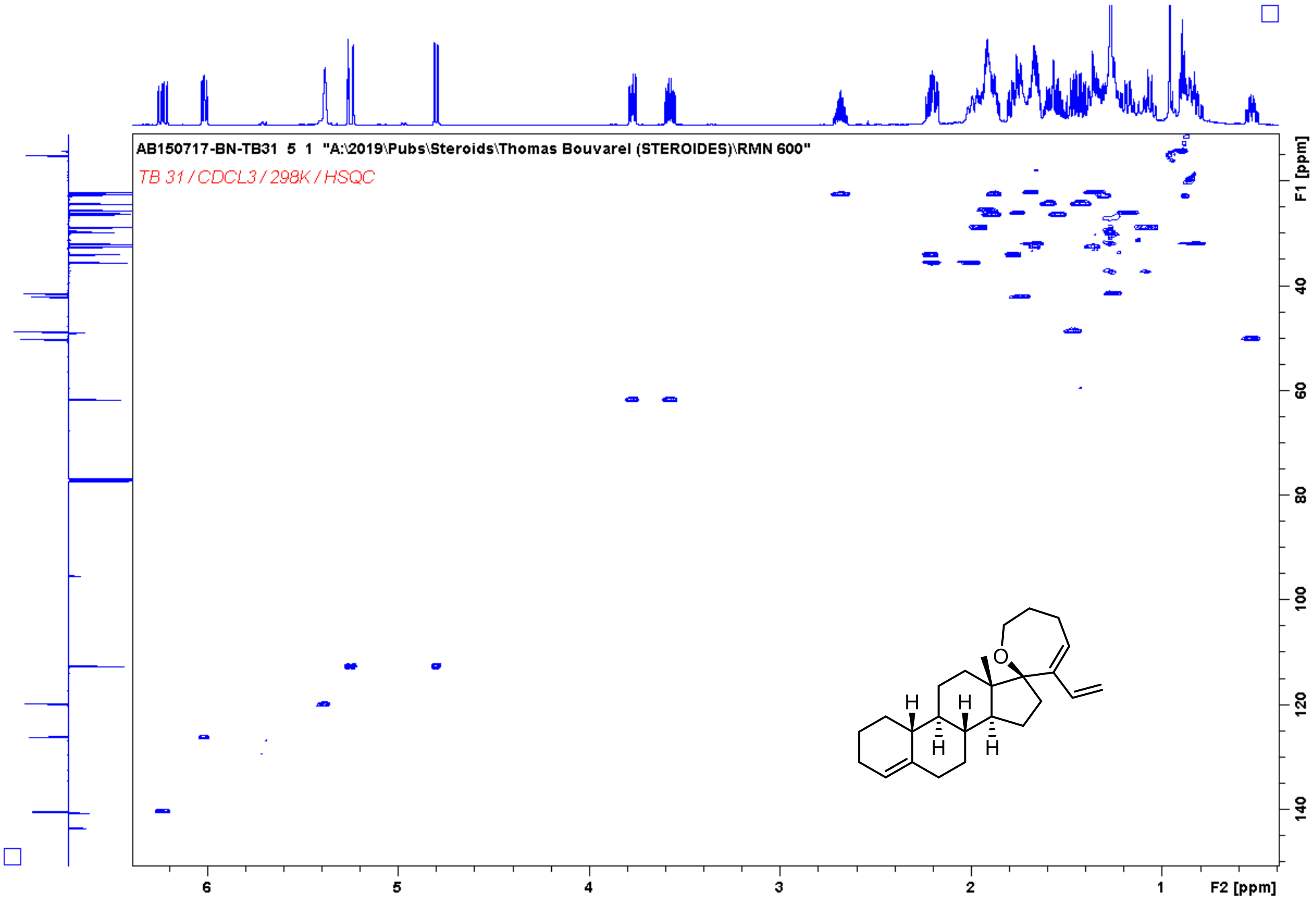

(2'*S*,8*R*,9*S*,10*R*,13*S*,14*S*)-13-Methyl-3'-vinyl-1,2,3,6,6',7,7',8,9,10,11,12,13,14,15,16-hexadecahydro-5'*H*-spiro[cyclopenta[*a*]phenanthrene-17,2'-oxepine] (9b): NOESY NMR (600 MHz, CDCl$_3$)

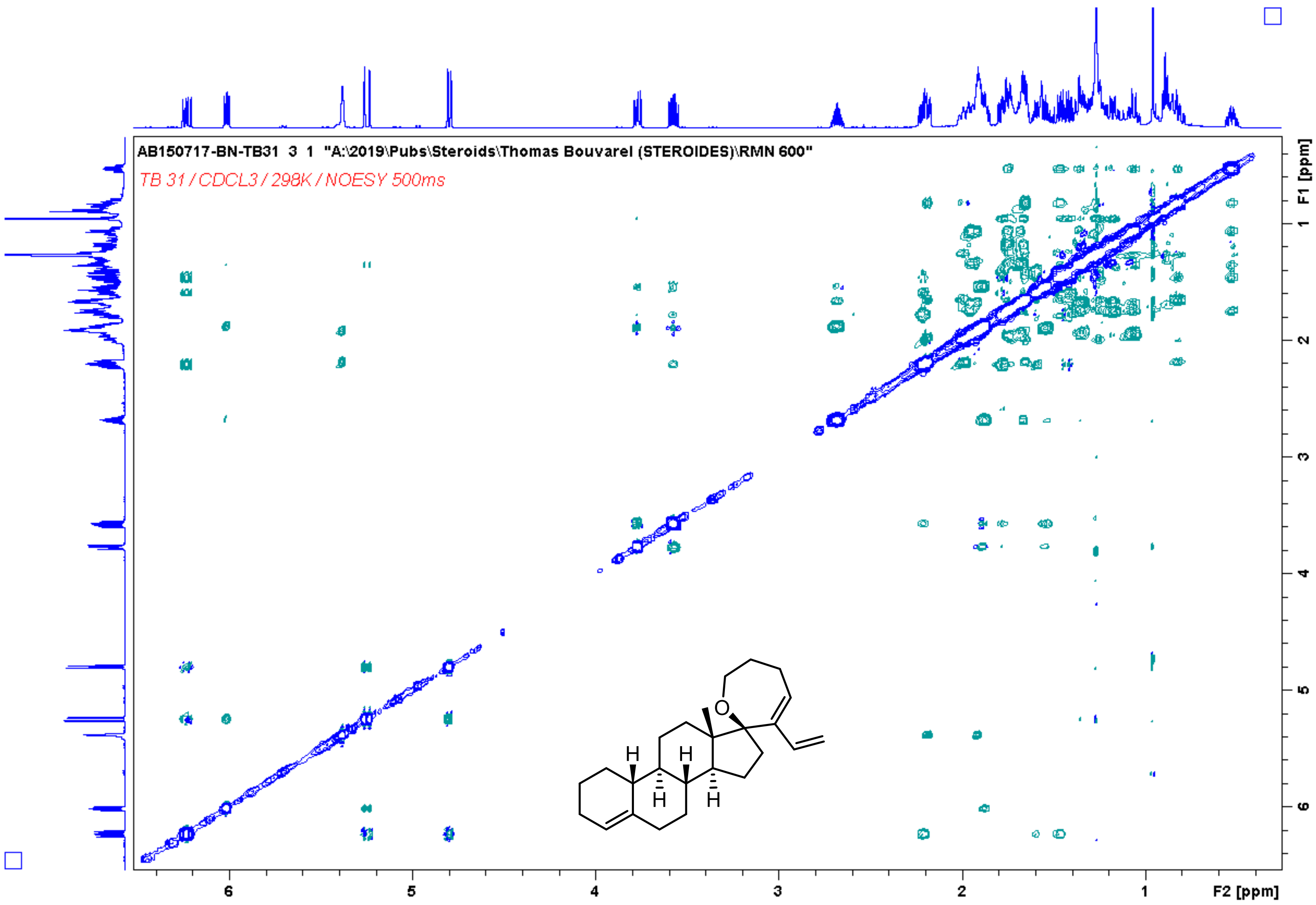

(8*R*,9*S*,13*S*,14*S*,17*R*)-3-Methoxy-13-methyl-3'-vinyl-6,7,8,9,11,12,13,14,15,16-decahydro-5'*H*,6'*H*,7'*H*-spiro[cyclopenta[*a*]phenanthrene-17,2'-oxepin] (10): ^{1}H NMR (600 MHz, $CDCl_3$)

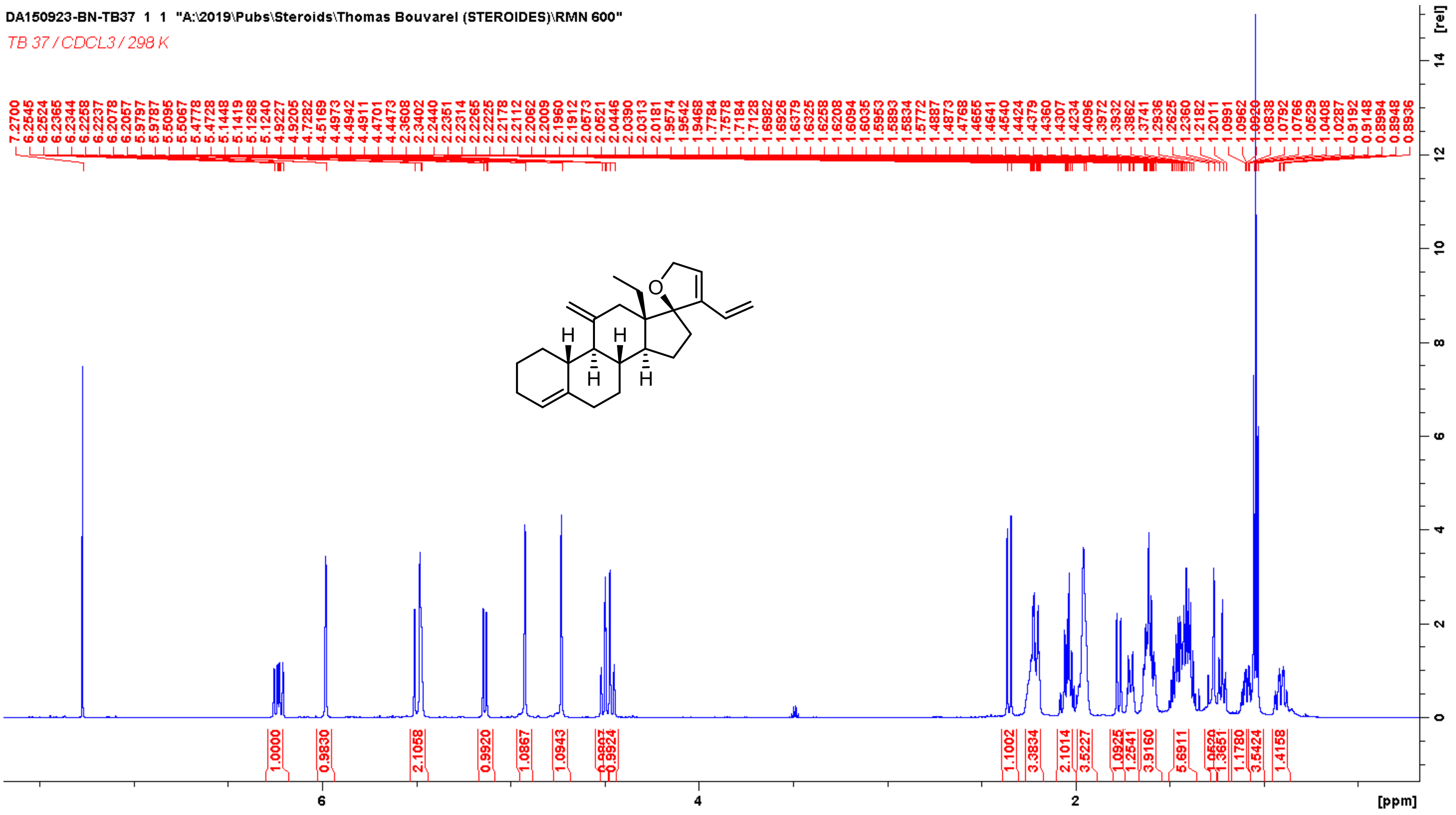

(8*R*,9*S*,13*S*,14*S*,17*R*)-3-Methoxy-13-methyl-3'-vinyl-6,7,8,9,11,12,13,14,15,16-decahydro-5'*H*,6'*H*,7'*H*-spiro[cyclopenta[*a*]phenanthrene-17,2'-oxepin] (10): ^{13}C NMR (150 MHz, $CDCl_3$)

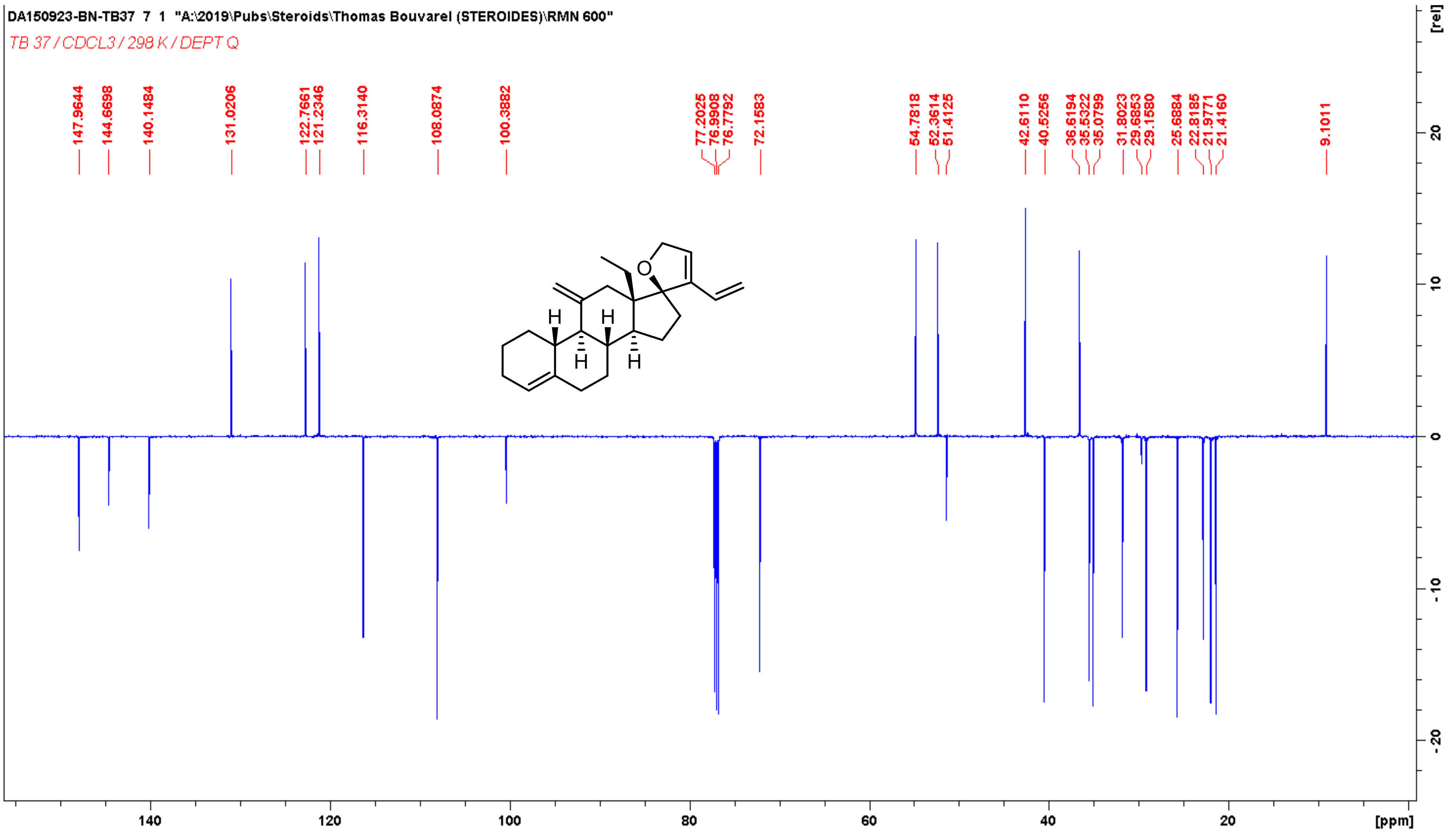

(8*R*,9*S*,13*S*,14*S*,17*R*)-3-Methoxy-13-methyl-3'-vinyl-6,7,8,9,11,12,13,14,15,16-decahydro-5'*H*,6'*H*,7'*H*-spiro[cyclopenta[*a*]phenanthrene-17,2'-oxepin] (10): COSY NMR (600 MHz, $CDCl_3$)

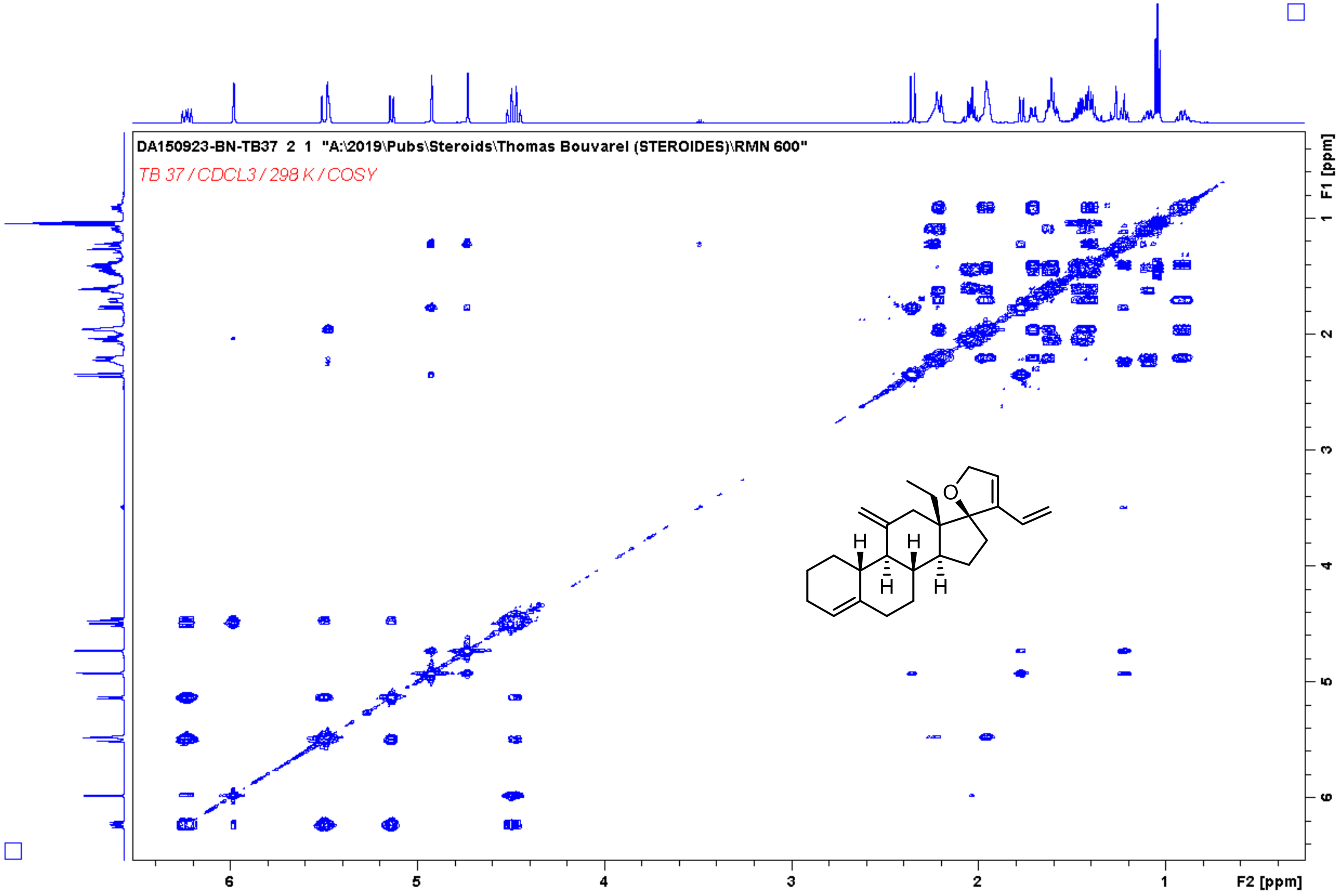

(8*R*,9*S*,13*S*,14*S*,17*R*)-3-Methoxy-13-methyl-3'-vinyl-6,7,8,9,11,12,13,14,15,16-decahydro-5'*H*,6'*H*,7'*H*-spiro[cyclopenta[*a*]phenanthrene-17,2'-oxepin] (10): HMBC NMR (600 MHz, CDCl$_3$)

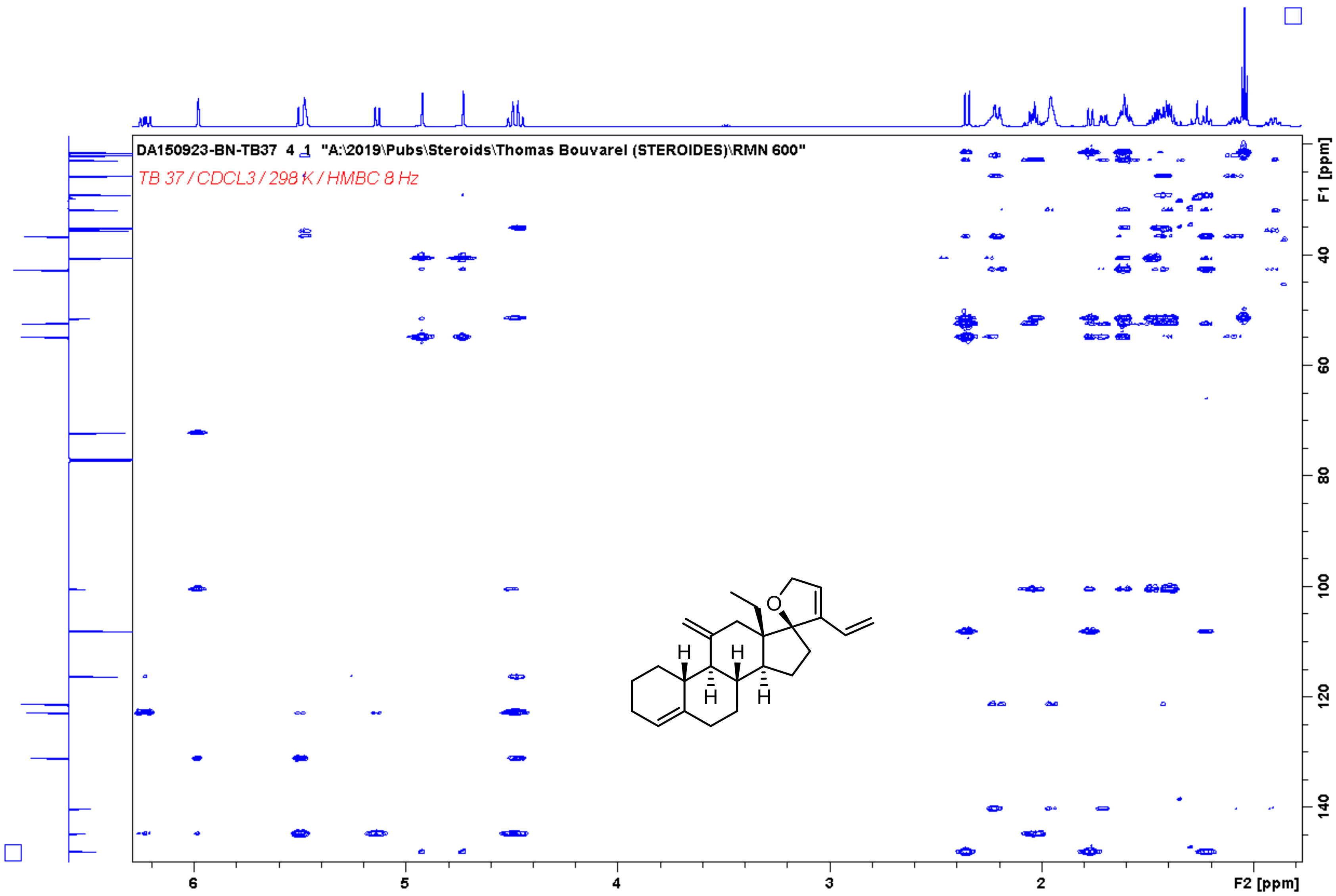

(8*R*,9*S*,13*S*,14*S*,17*R*)-3-Methoxy-13-methyl-3'-vinyl-6,7,8,9,11,12,13,14,15,16-decahydro-5'*H*,6'*H*,7'*H*-spiro[cyclopenta[*a*]phenanthrene-17,2'-oxepin] (10): HSQC NMR (600 MHz, $CDCl_3$)

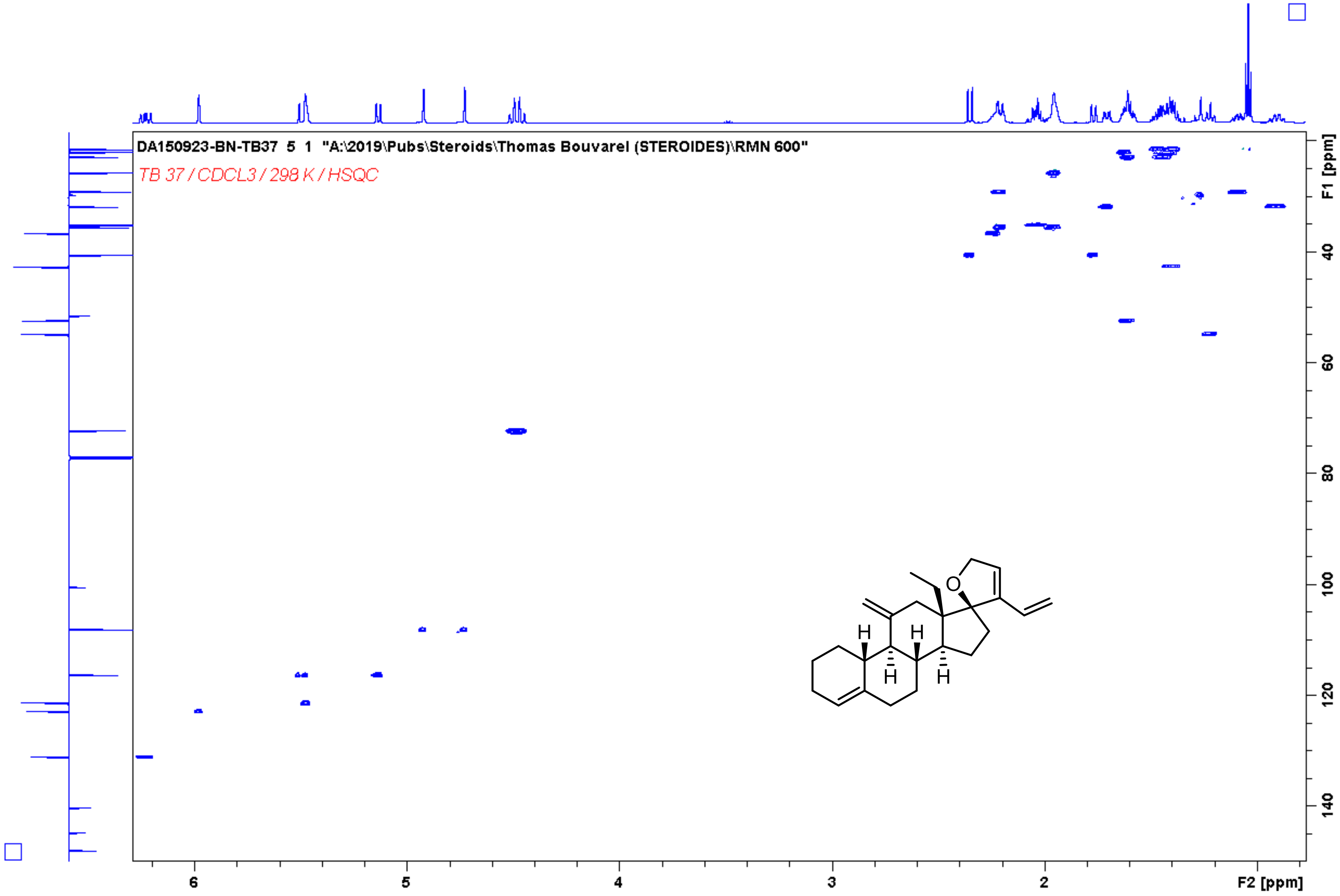

(8*R*,9*S*,13*S*,14*S*,17*R*)-3-Methoxy-13-methyl-3'-vinyl-6,7,8,9,11,12,13,14,15,16-decahydro-5'*H*,6'*H*,7'*H*-spiro[cyclopenta[*a*]phenanthrene-17,2'-oxepin] (10): NOESY NMR (600 MHz, $CDCl_3$)

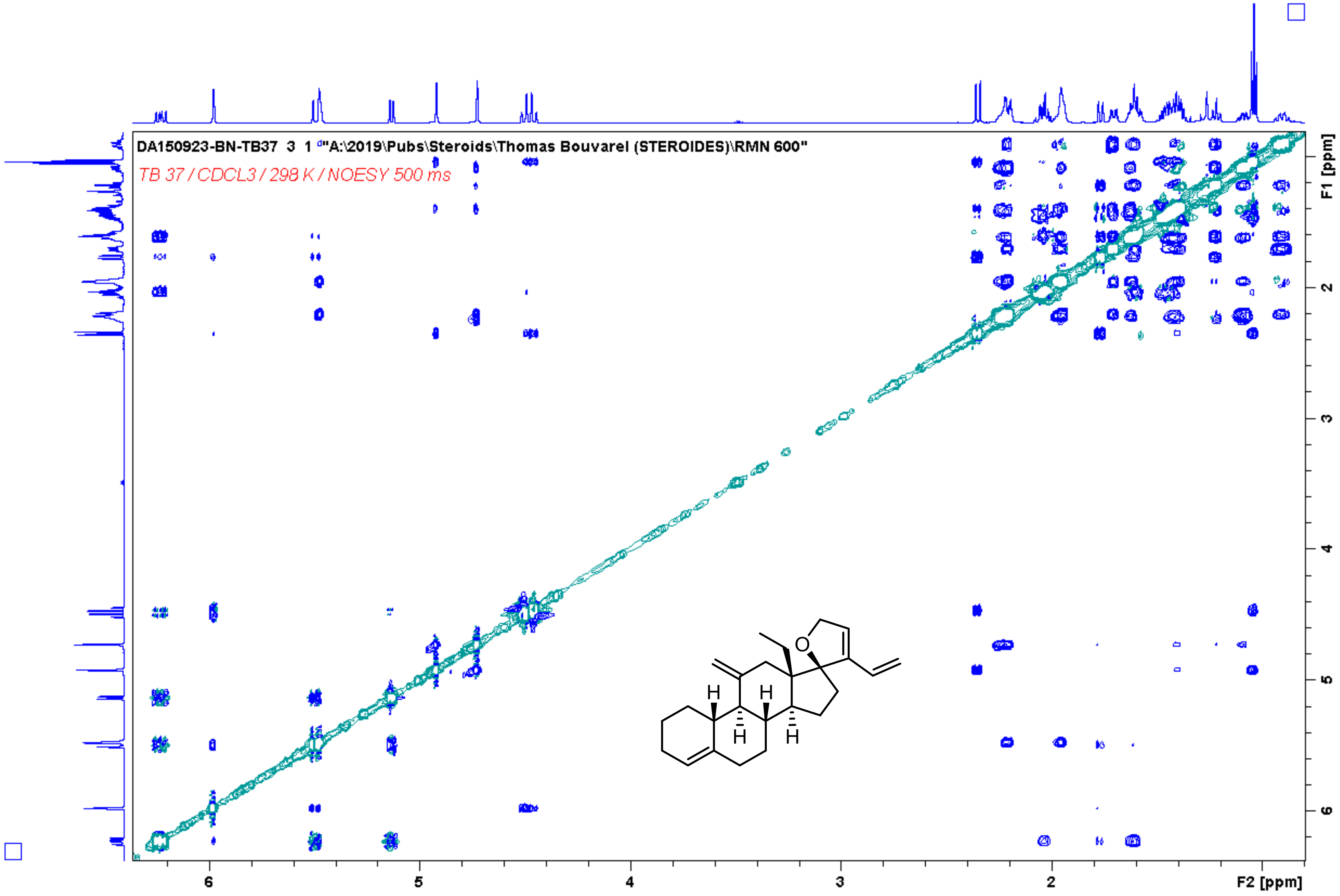

(3'S,5a'S,8R,8a'R,8b'S,9S,13S,14S)-3-Methoxy-13-methyl-7'-phenyl-1',5',5a',6,7,8,8b',9,11,12,13,14,15,16-tetradecahydrospiro[cyclopenta[*a*]phenanthrene-17,3'-furo[3,4-*e*]isoindole]-6',8'(7'*H*,8a'*H*)-dione (16a): ^{1}H NMR (600 MHz, $CDCl_3$)

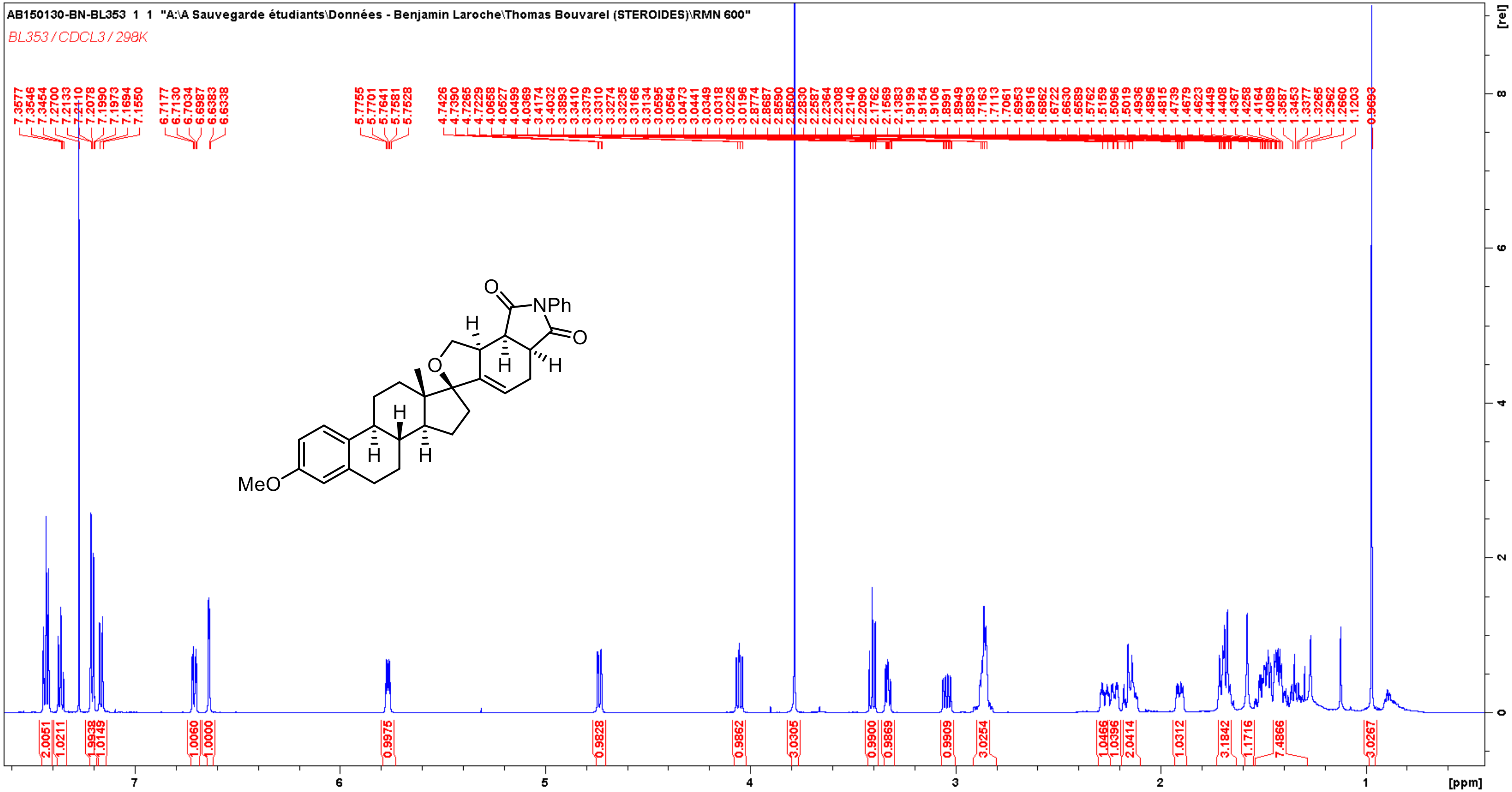

(3'*S*,5a'*S*,8*R*,8a'*R*,8b'*S*,9*S*,13*S*,14*S*)-3-Methoxy-13-methyl-7'-phenyl-1',5',5a',6,7,8,8b',9,11,12,13,14,15,16-tetradecahydrospiro[cyclopenta[*a*]phenanthrene-17,3'-furo[3,4-*e*]isoindole]-6',8'(7'*H*,8a'*H*)-dione (16a): ^{13}C NMR (150 MHz, $CDCl_3$)

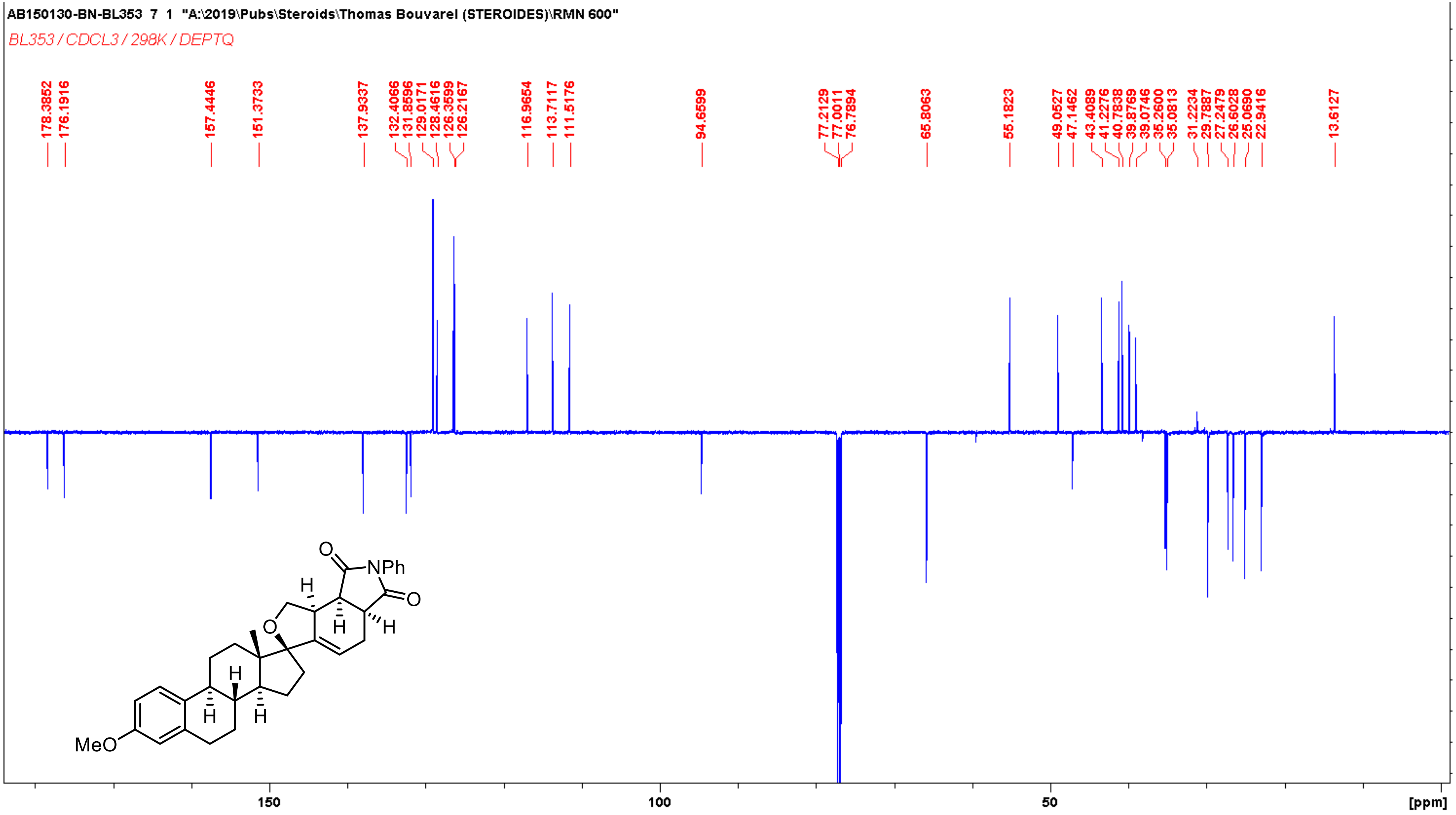

(3'*S*,5a'*S*,8*R*,8a'*R*,8b'*S*,9*S*,13*S*,14*S*)-3-Methoxy-13-methyl-7'-phenyl-1',5',5a',6,7,8,8b',9,11,12,13,14,15,16-tetradecahydrospiro[cyclopenta[*a*]phenanthrene-17,3'-furo[3,4-*e*]isoindole]-6',8'(7'H,8a'*H*)-dione (16a): COSY NMR (600 MHz,

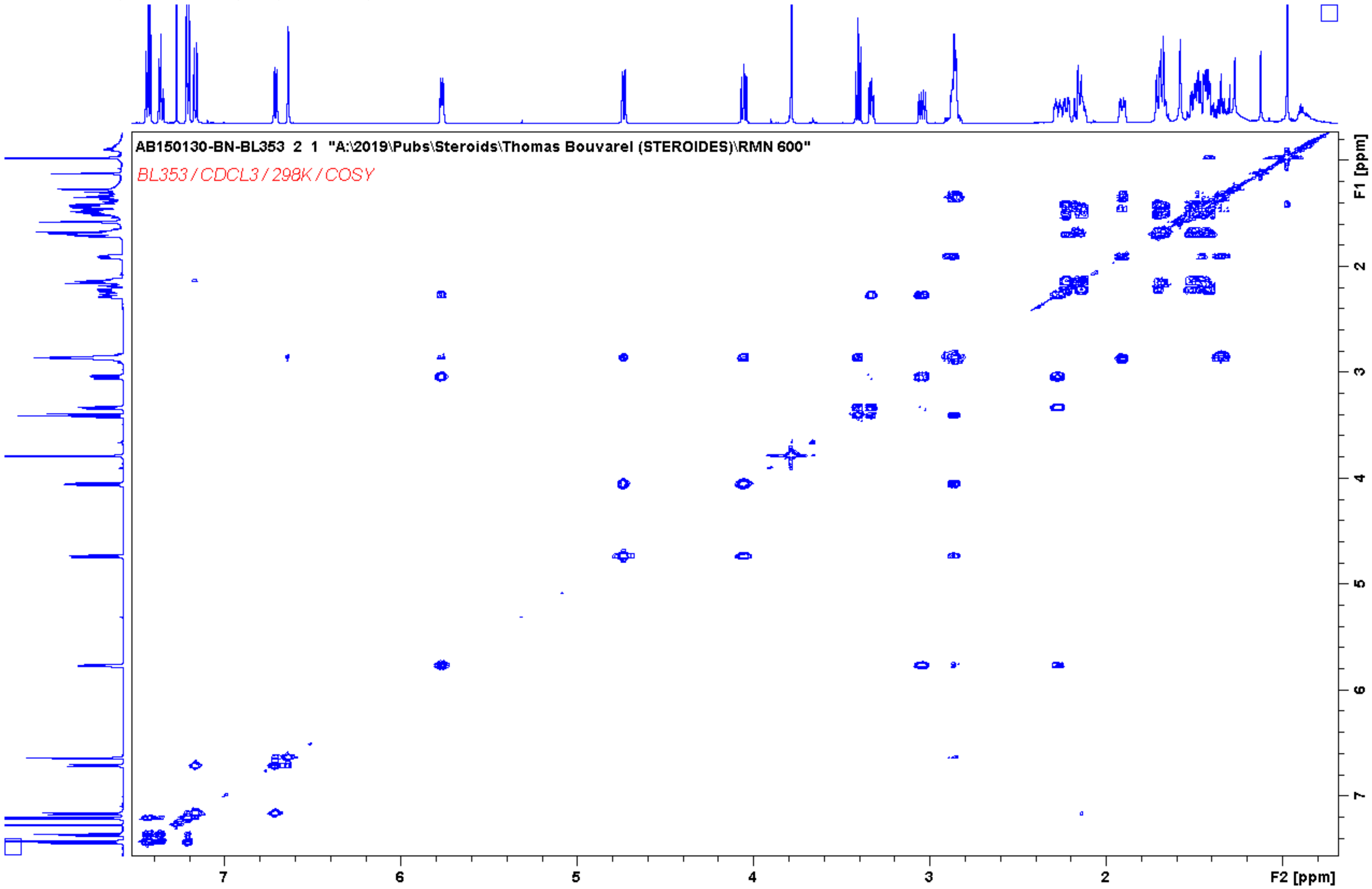

(3'*S*,5a'*S*,8*R*,8a'*R*,8b'*S*,9*S*,13*S*,14*S*)-3-Methoxy-13-methyl-7'-phenyl-1',5',5a',6,7,8,8b',9,11,12,13,14,15,16-tetradecahydrospiro[cyclopenta[*a*]phenanthrene-17,3'-furo[3,4-*e*]isoindole]-6',8'(7'*H*,8a'*H*)-dione (16a): HMBC NMR (600 MHz,

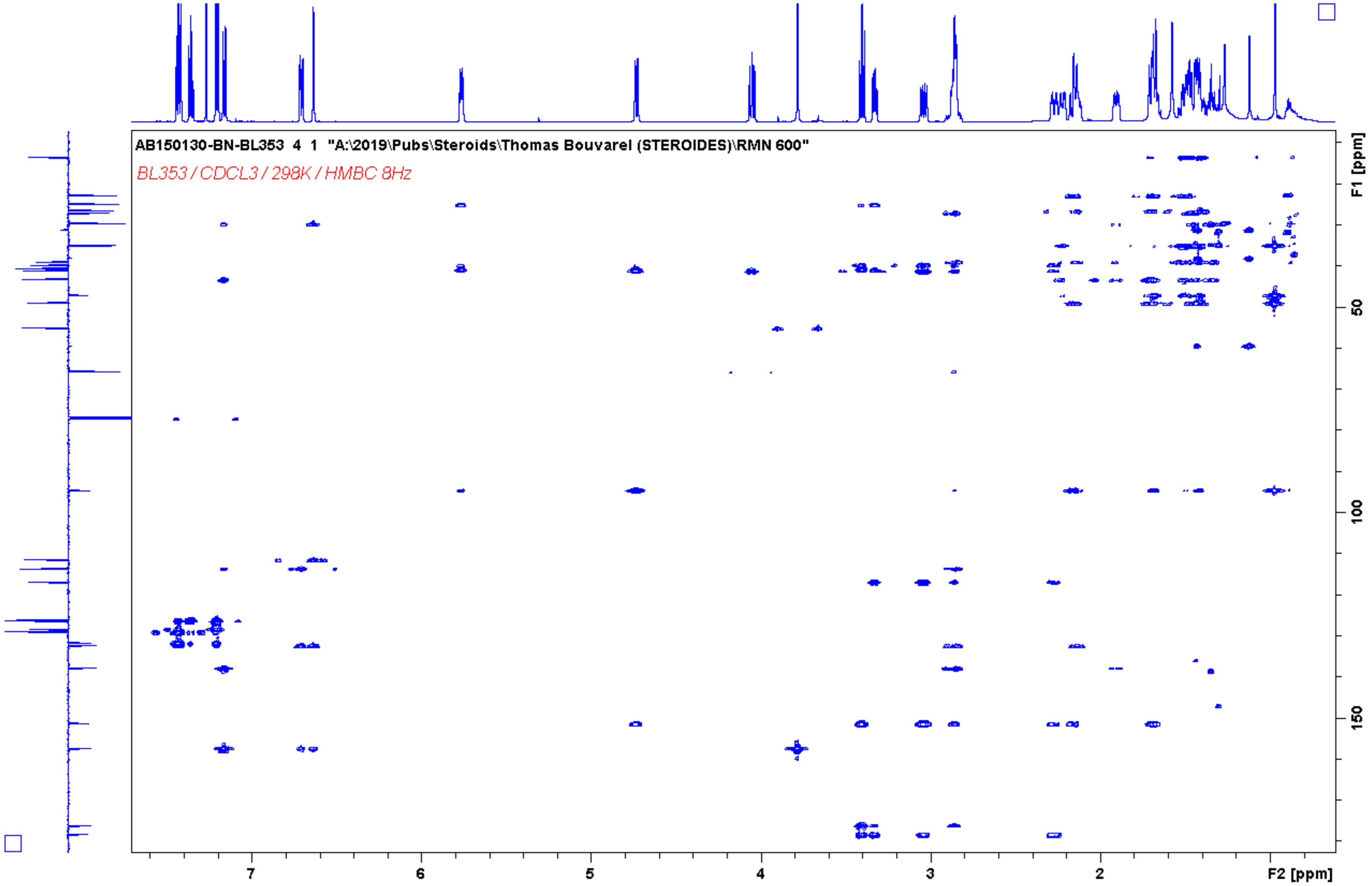

(3'*S*,5a'*S*,8*R*,8a'*R*,8b'*S*,9*S*,13*S*,14*S*)-3-Methoxy-13-methyl-7'-phenyl-1',5',5a',6,7,8,8b',9,11,12,13,14,15,16-tetradecahydrospiro[cyclopenta[*a*]phenanthrene-17,3'-furo[3,4-*e*]isoindole]-6',8'(7'*H*,8a'*H*)-dione (16a): HSQC NMR (600 MHz,

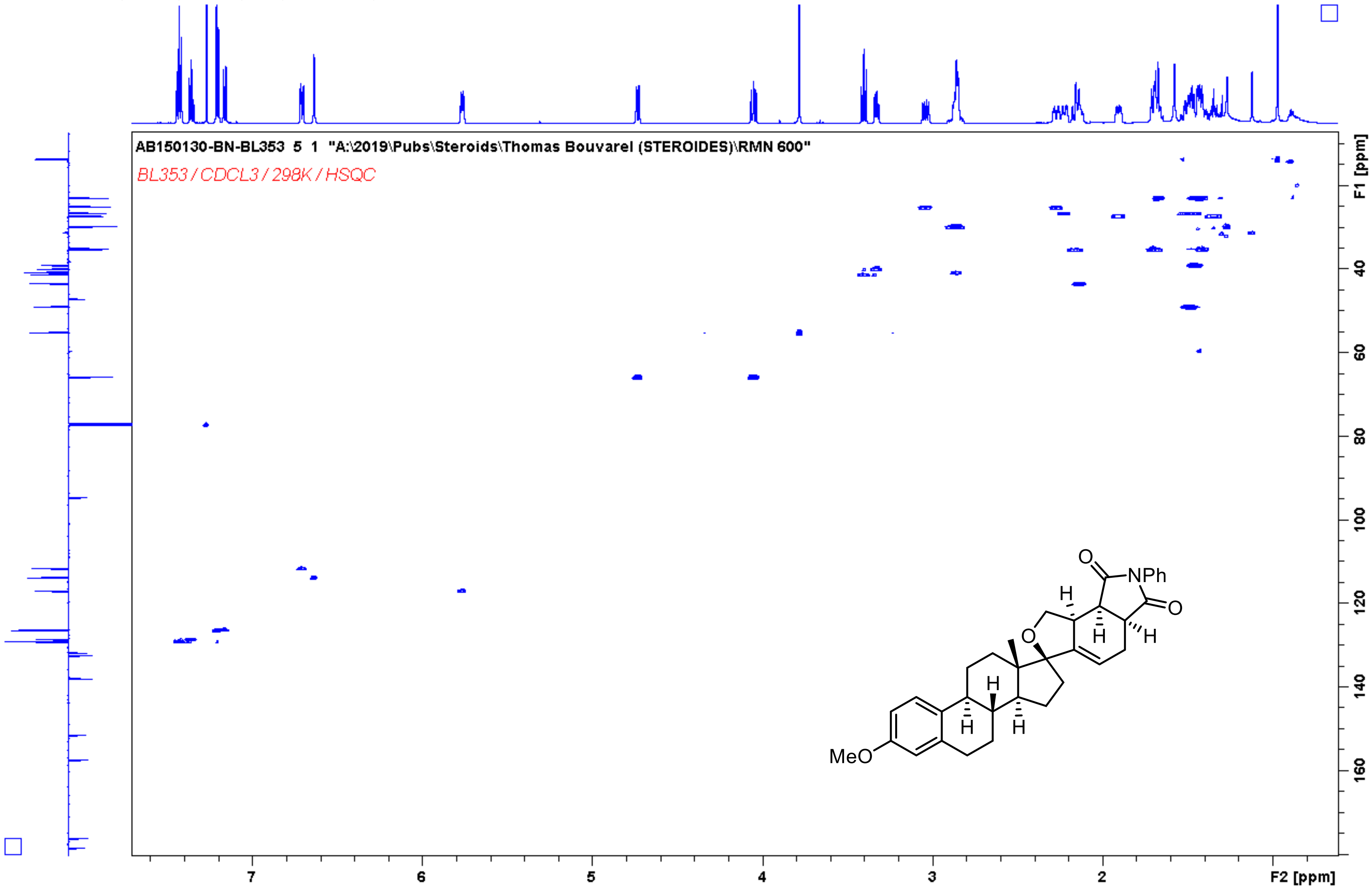

(3'*S*,5a'*S*,8*R*,8a'*R*,8b'*S*,9*S*,13*S*,14*S*)-3-Methoxy-13-methyl-7'-phenyl-1',5',5a',6,7,8,8b',9,11,12,13,14,15,16-tetradecahydrospiro[cyclopenta[*a*]phenanthrene-17,3'-furo[3,4-*e*]isoindole]-6',8'(7'*H*,8a'*H*)-dione (16a): NOESY NMR (600 MHz,

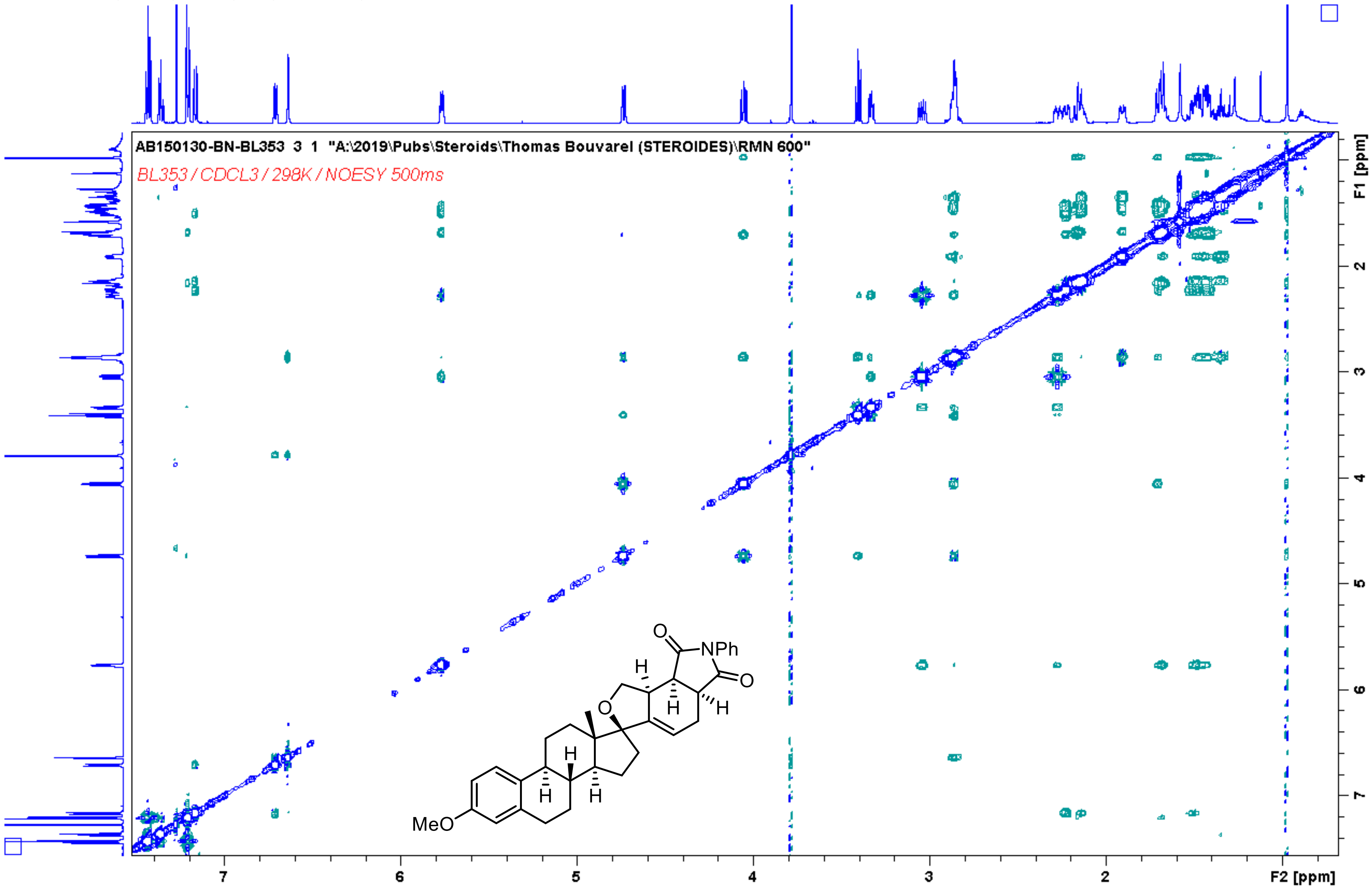

(3*S*,5a*S*,8a*R*,8b*S*,8'*R*,9'*S*,13'*S*,14'*S*)-3'-Methoxy-13'-methyl-5,5a,6',7',8',9',11',12',13',14',15',16'-dodecahydro-1*H*-spiro[benzo[1,2-*c*:3,4-*c*']difuran-3,17'-cyclopenta[*a*]phenanthrene]-6,8(8a*H*,8b*H*)-dione (16b): ^{1}H NMR (600 MHz, $CDCl_3$)

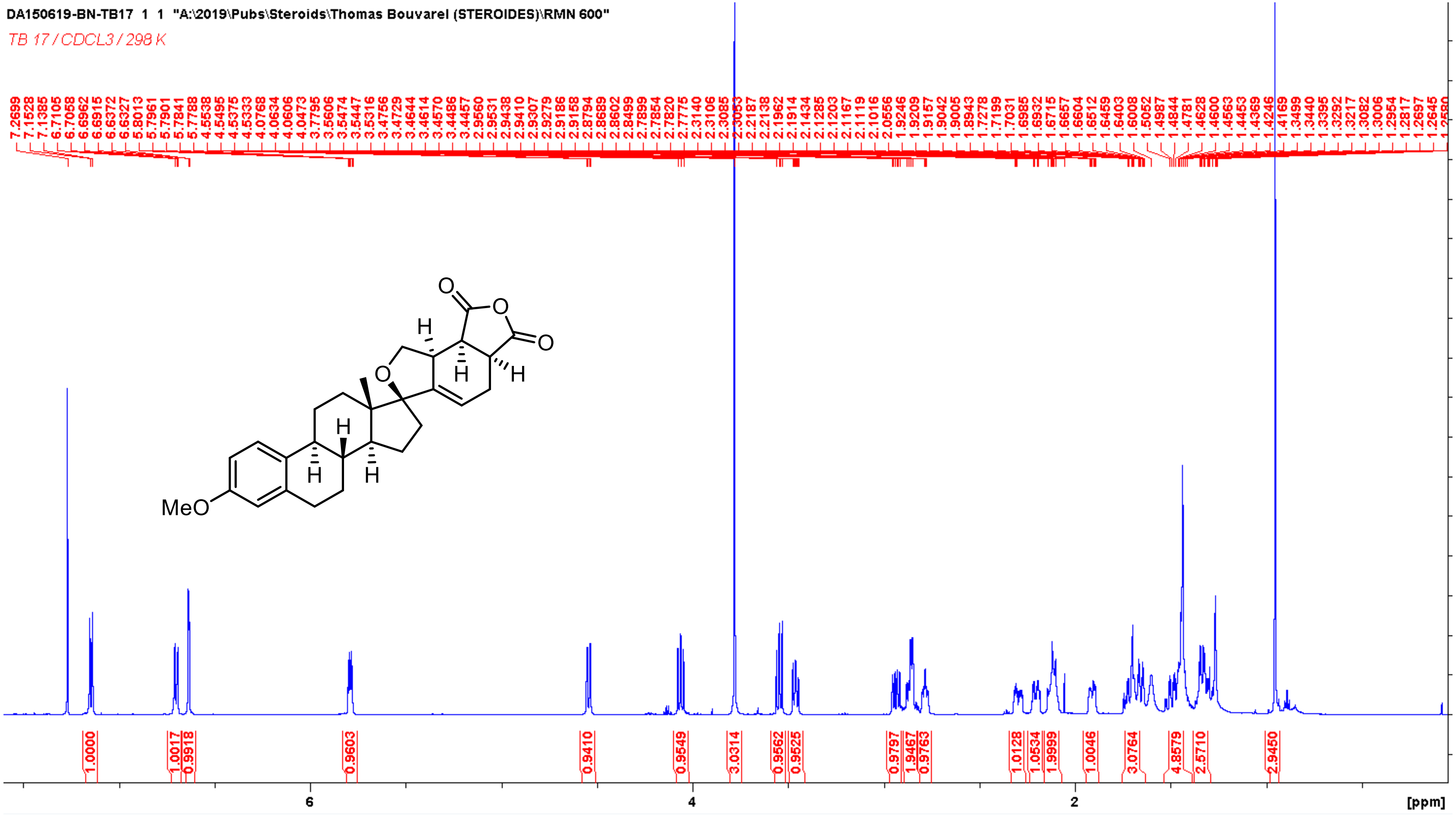

(3*S*,5a*S*,8a*R*,8b*S*,8'*R*,9'*S*,13'*S*,14'*S*)-3'-Methoxy-13'-methyl-5,5a,6',7',8',9',11',12',13',14',15',16'-dodecahydro-1*H*-spiro[benzo[1,2-*c*:3,4-*c*']difuran-3,17'-cyclopenta[*a*]phenanthrene]-6,8(8a*H*,8b*H*)-dione (16b): ^{13}C NMR (150 MHz, CDCl$_3$)

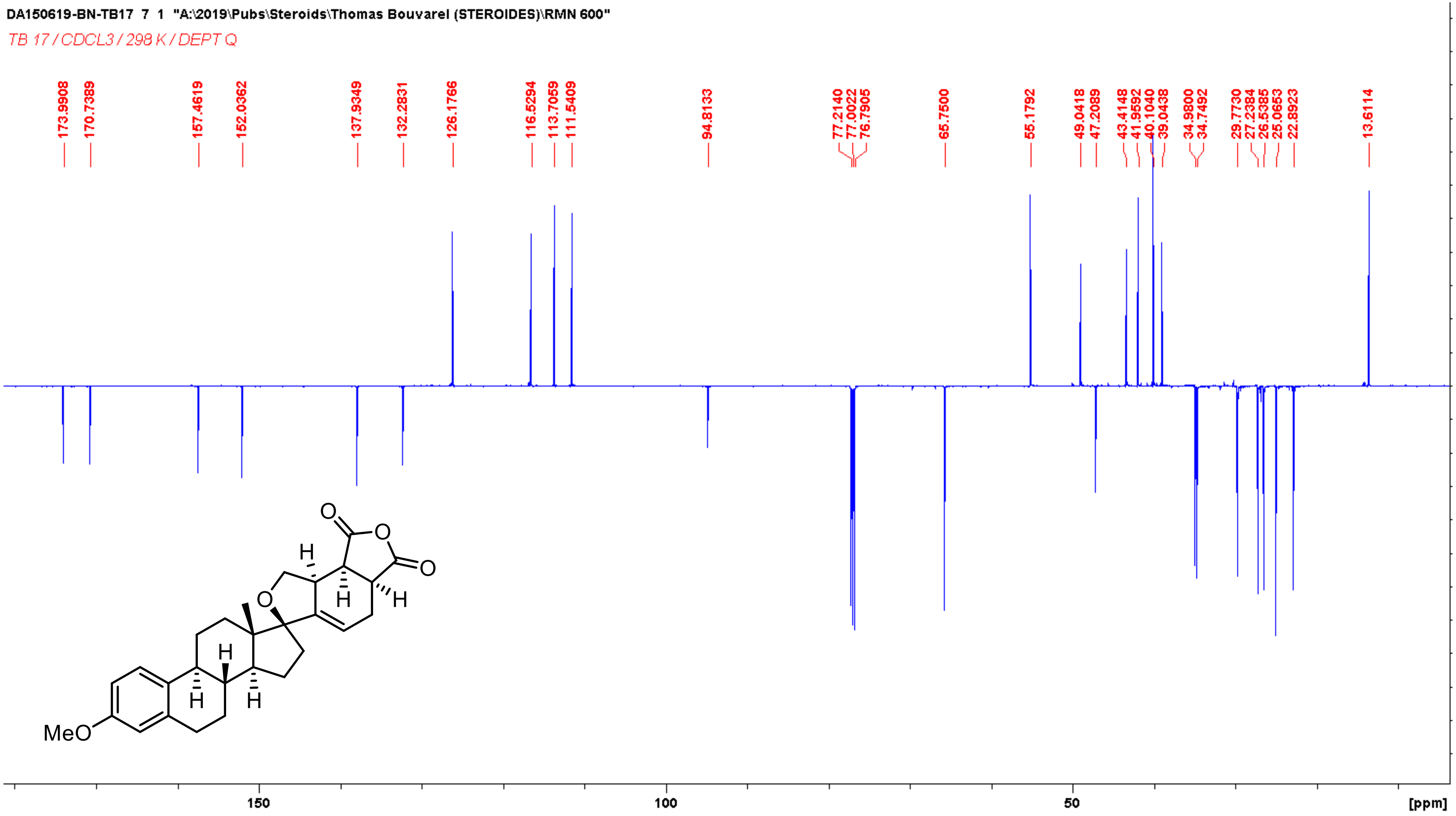

(3*S*,5a*S*,8a*R*,8b*S*,8'*R*,9'*S*,13'*S*,14'*S*)-3'-Methoxy-13'-methyl-5,5a,6',7',8',9',11',12',13',14',15',16'-dodecahydro-1*H*-spiro[benzo[1,2-*c*:3,4-*c*']difuran-3,17'-cyclopenta[*a*]phenanthrene]-6,8(8a*H*,8b*H*)-dione (16b): COSY NMR (600 MHz, $CDCl_3$)

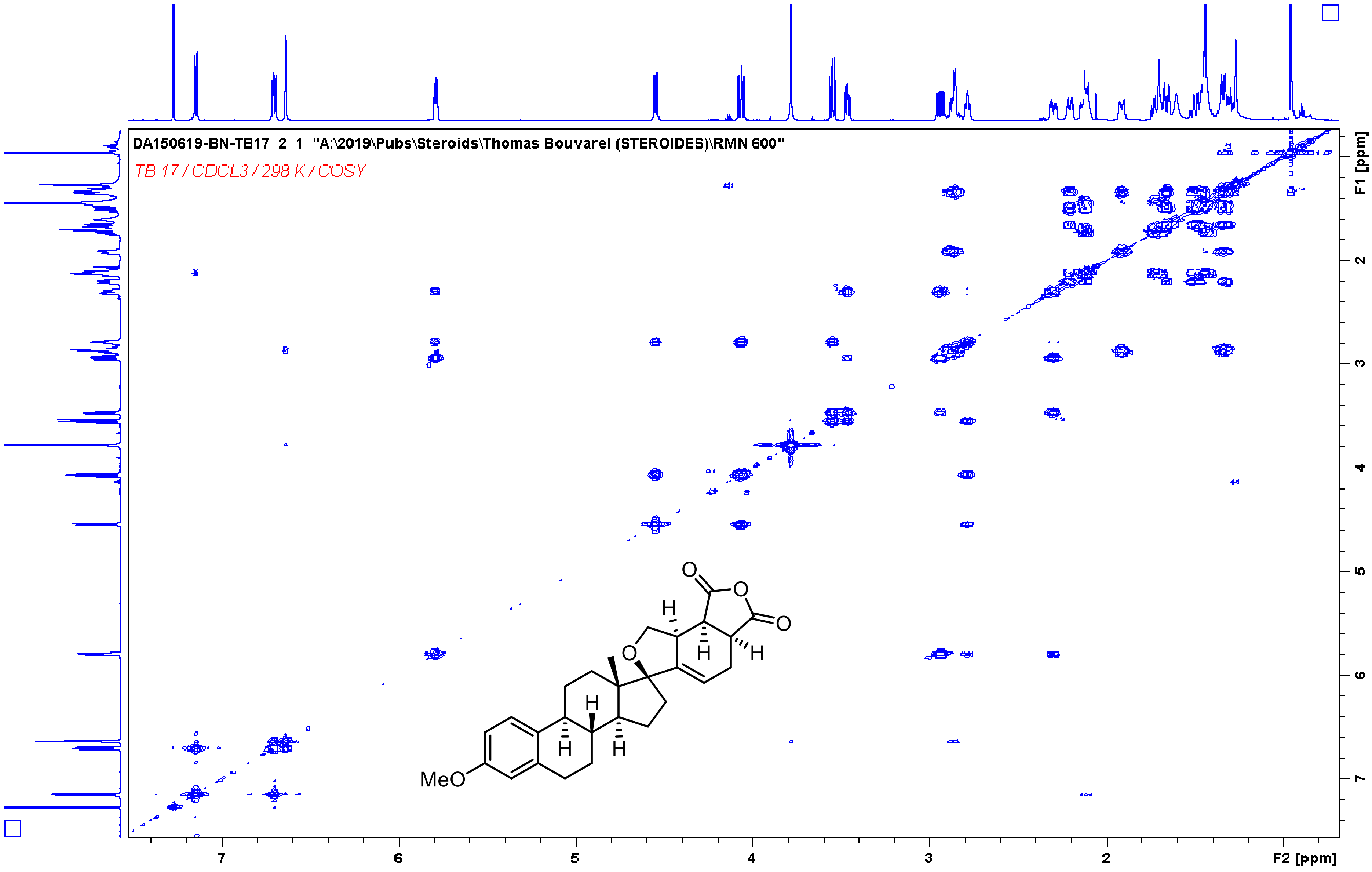

(3*S*,5a*S*,8a*R*,8b*S*,8'*R*,9'*S*,13'*S*,14'*S*)-3'-Methoxy-13'-methyl-5,5a,6',7',8',9',11',12',13',14',15',16'-dodecahydro-1*H*-spiro[benzo[1,2-*c*:3,4-*c*']difuran-3,17'-cyclopenta[*a*]phenanthrene]-6,8(8a*H*,8b*H*)dione (16b): HMBC NMR (600 MHz, $CDCl_3$)

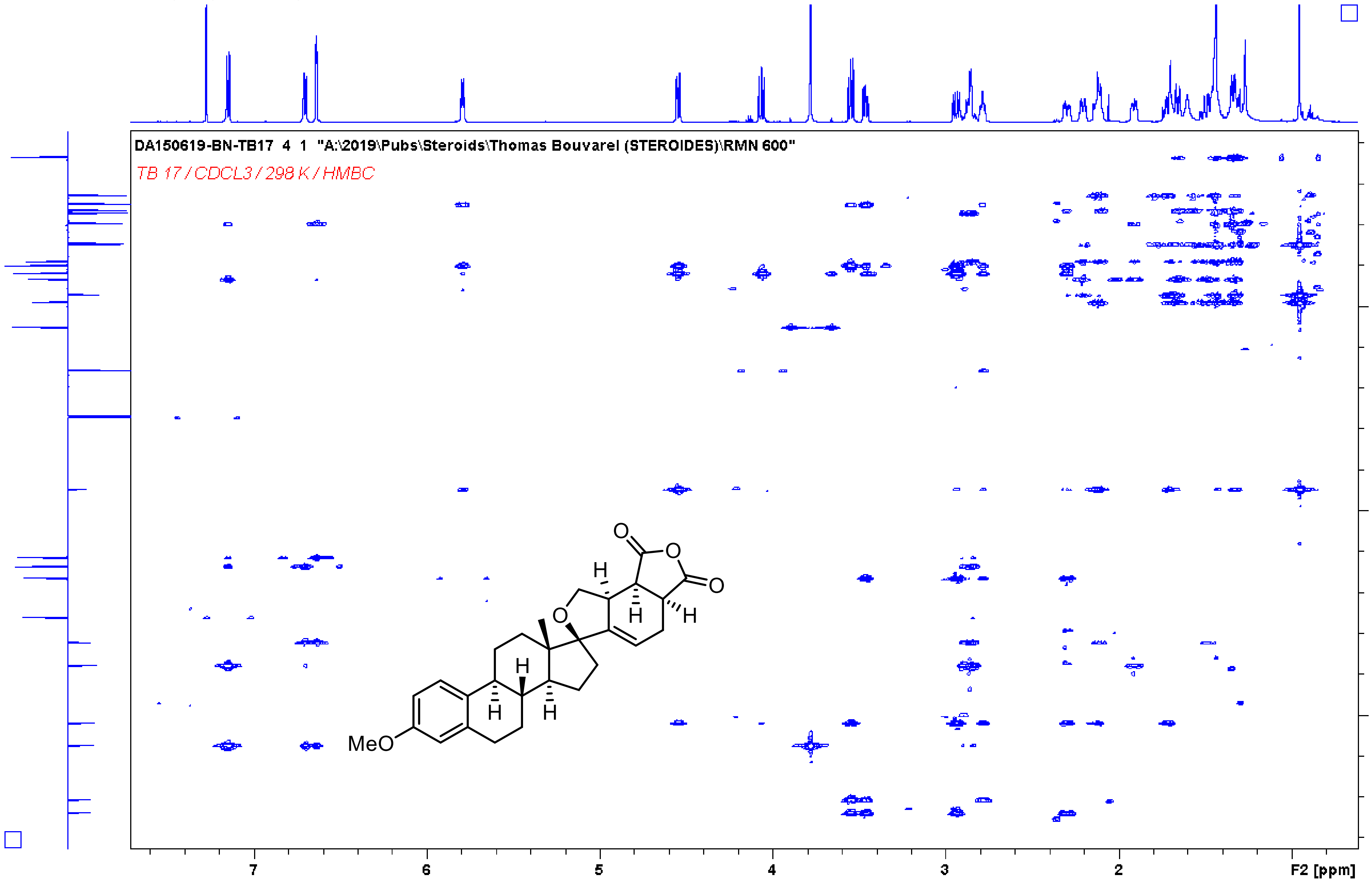

(3*S*,5a*S*,8a*R*,8b*S*,8'*R*,9'*S*,13'*S*,14'*S*)-3'-Methoxy-13'-methyl-5,5a,6',7',8',9',11',12',13',14',15',16'-dodecahydro-1*H*-spiro[benzo[1,2-*c*:3,4-*c*']difuran-3,17'-cyclopenta[*a*]phenanthrene]-6,8(8a*H*,8b*H*)-dione (16b): HSQC NMR (600 MHz, $CDCl_3$)

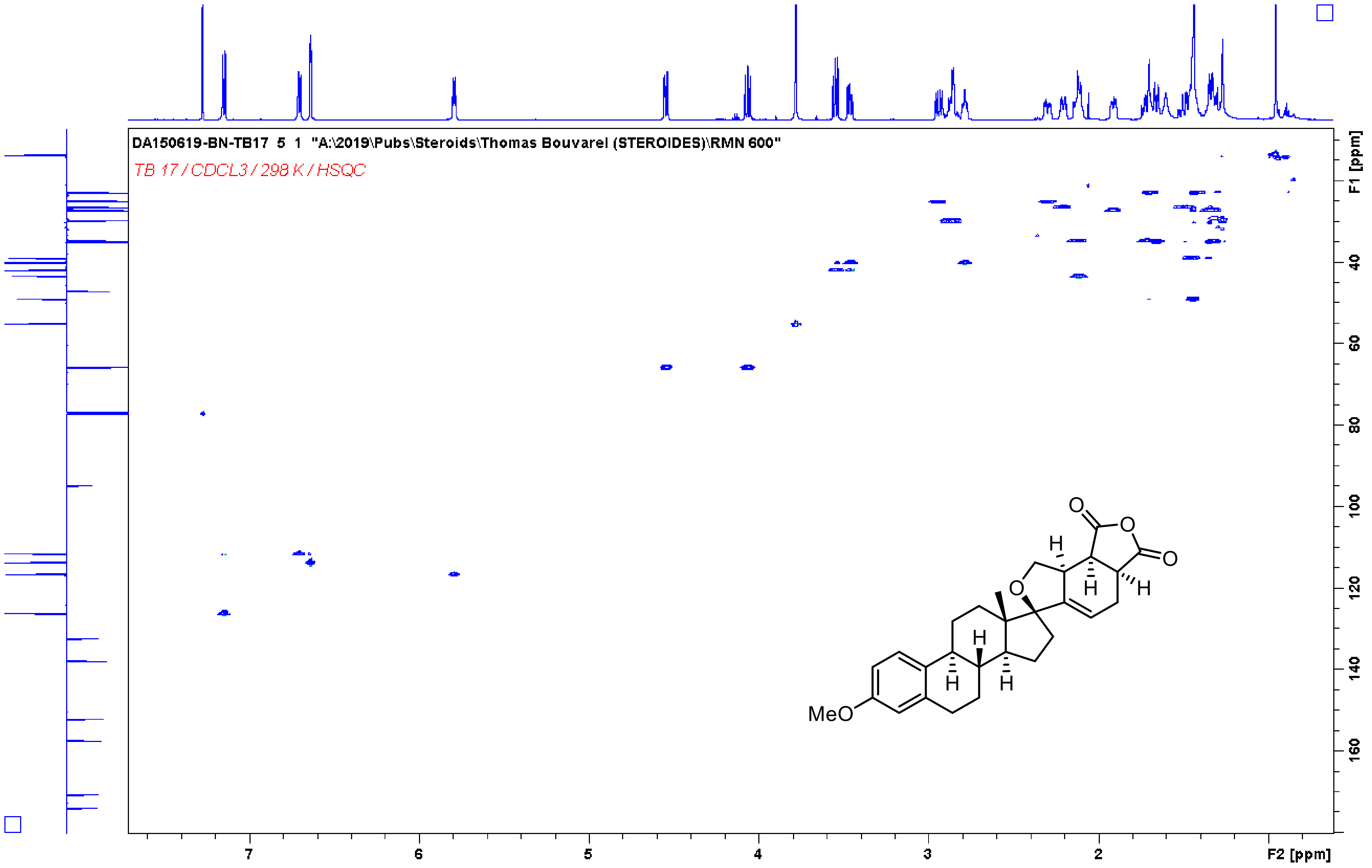

(3*S*,5a*S*,8a*R*,8b*S*,8'*R*,9'*S*,13'*S*,14'*S*)-3'-Methoxy-13'-methyl-5,5a,6',7',8',9',11',12',13',14',15',16'-dodecahydro-1*H*-spiro[benzo[1,2-*c*:3,4-*c*']difuran-3,17'-cyclopenta[*a*]phenanthrene]-6,8(8a*H*,8b*H*)dione (16b): NOESY NMR (600 MHz, $CDCl_3$)

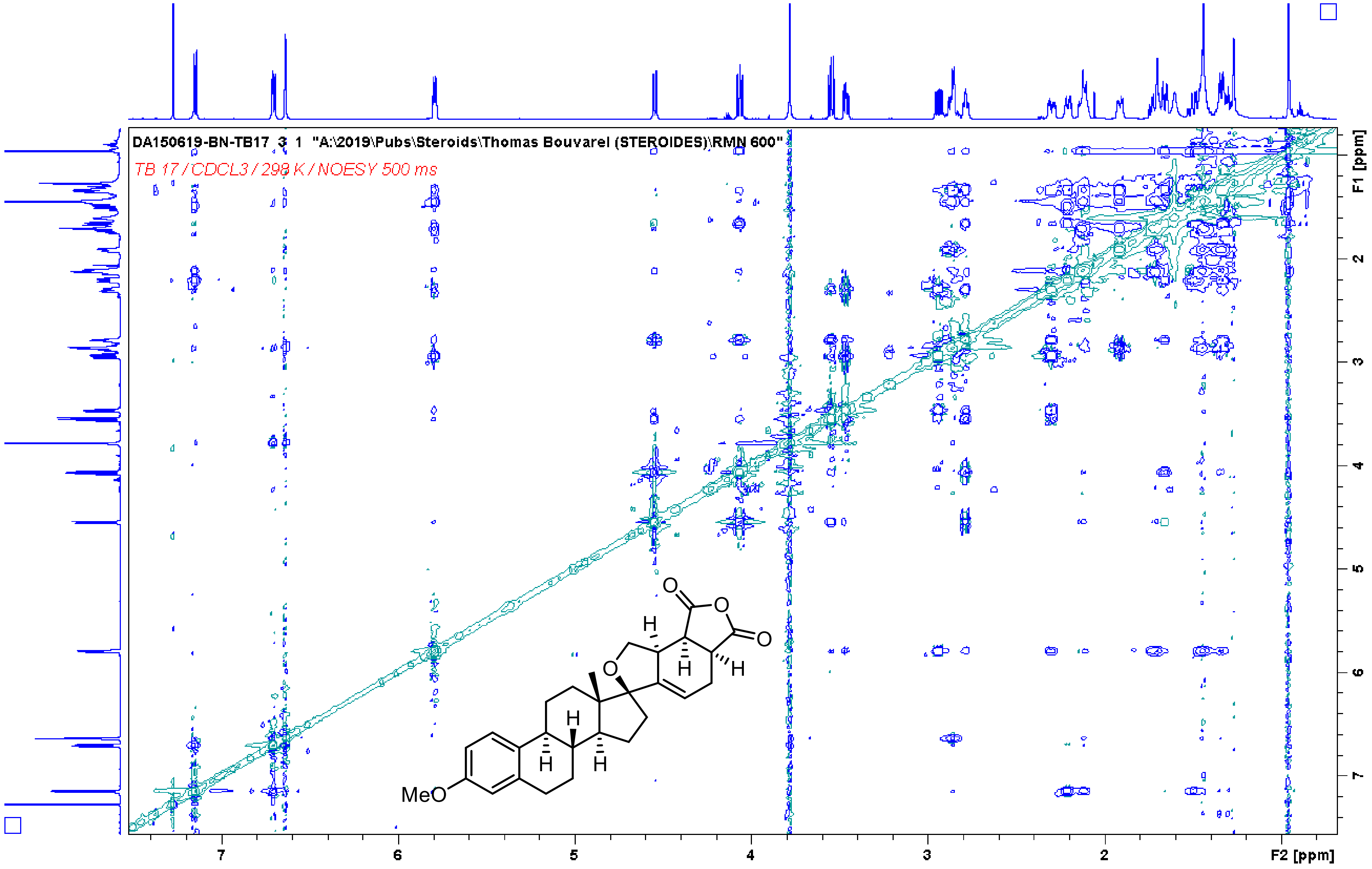

(1'*S*,3a'*R*,8*R*,9*S*,13*S*,14*S*)-dimethyl 3-methoxy-13-methyl-3a',6,6',7,8,9,11,12,13,14,15,16-dodecahydro-3'*H*-spiro[cyclopenta[*a*]phenanthrene-17,1'-isobenzofuran]-4',5'-dicarboxylate (16c): ^{1}H NMR (600 MHz, $CDCl_3$)

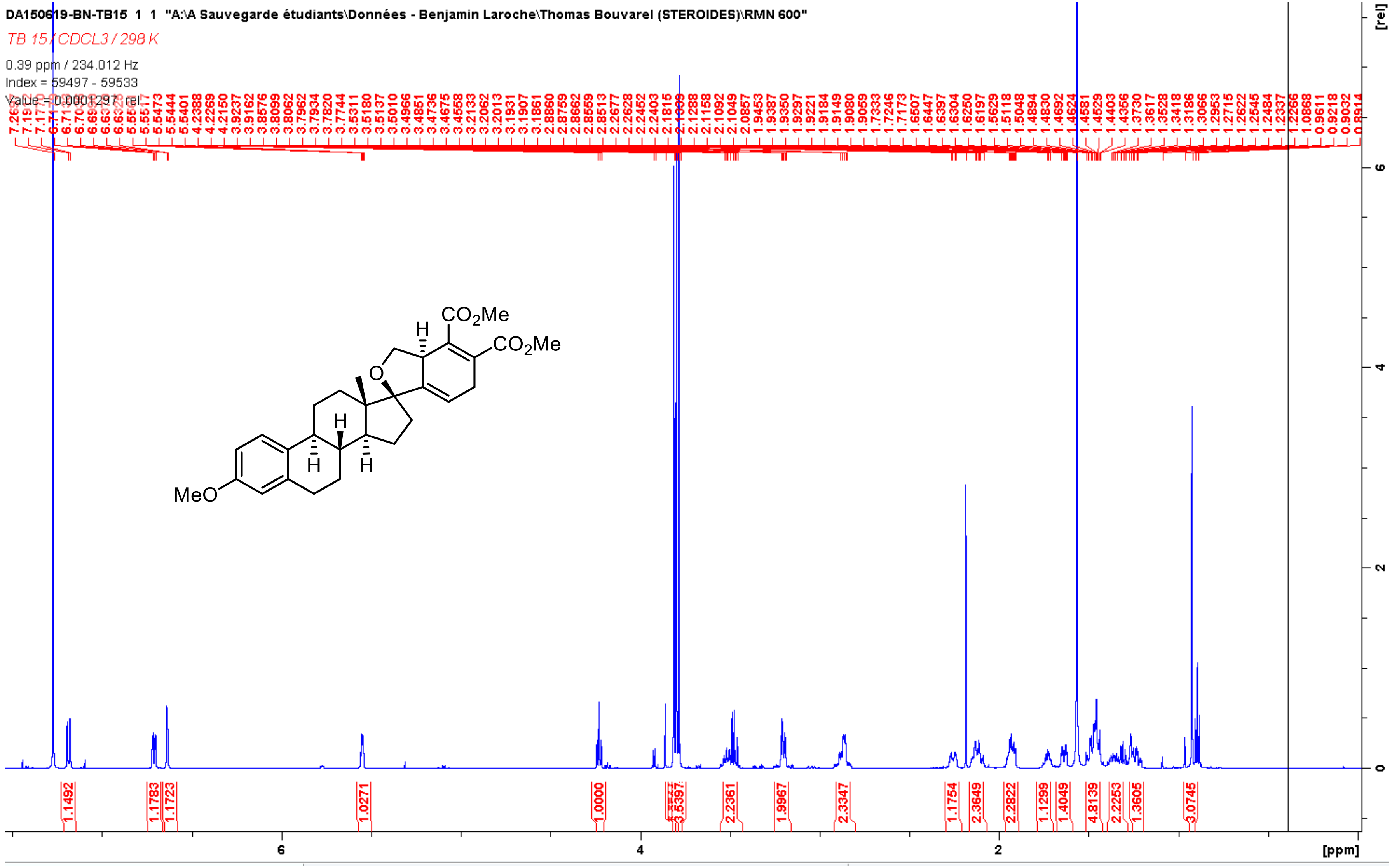

(1'*S*,3a'*R*,8*R*,9*S*,13*S*,14*S*)-Dimethyl 3-methoxy-13-methyl-3a',6,6',7,8,9,11,12,13,14,15,16-dodecahydro-3'*H*-spiro[cyclopenta[*a*]phenanthrene-17,1'-isobenzofuran]-4',5'-dicarboxylate (16c): ^{13}C NMR (150 MHz, $CDCl_3$)

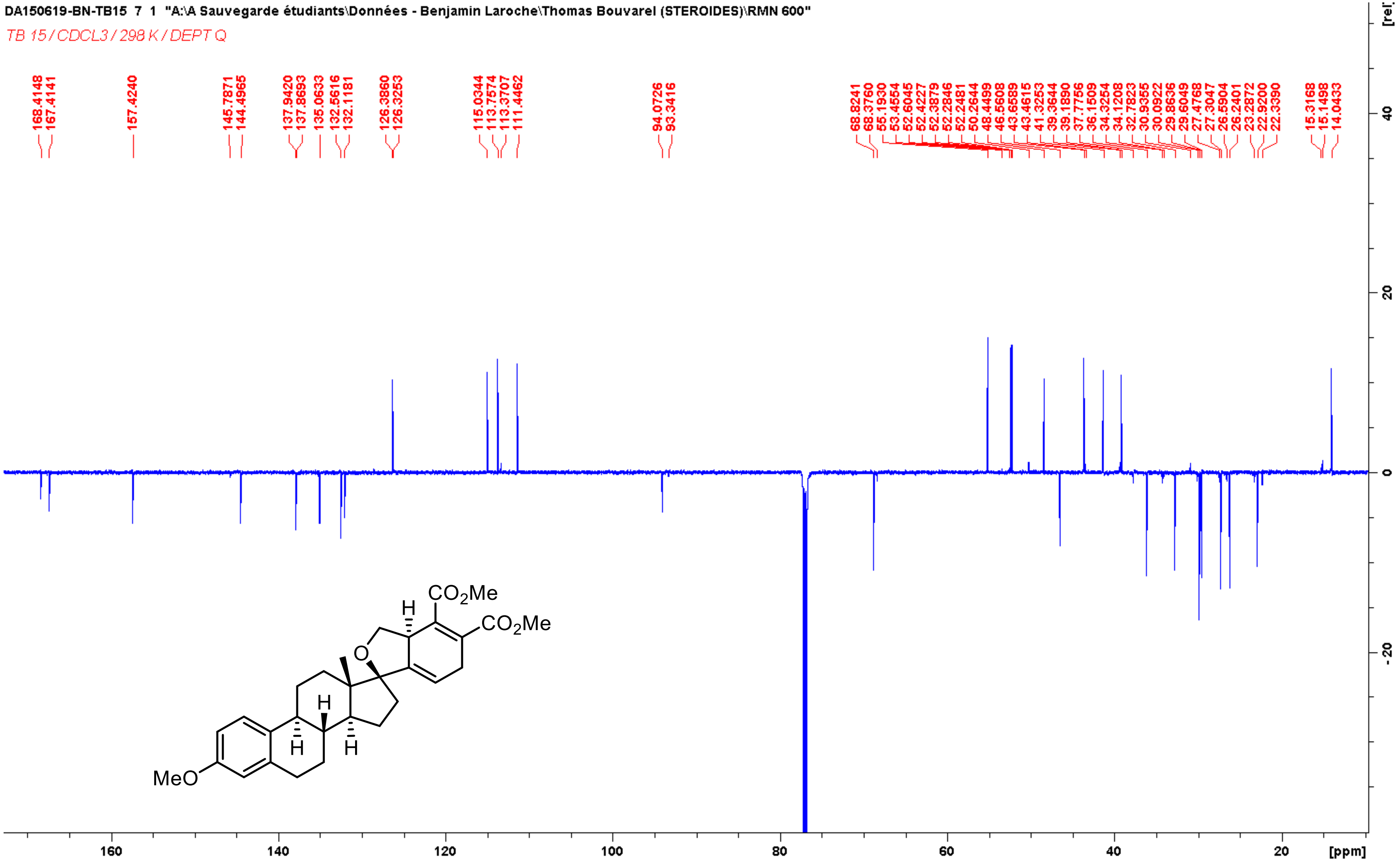

(1'*S*,3a'*R*,8*R*,9*S*,13*S*,14*S*)-Dimethyl 3-methoxy-13-methyl-3a',6,6',7,8,9,11,12,13,14,15,16-dodecahydro-3'*H*-spiro[cyclopenta[*a*]phenanthrene-17,1'-isobenzofuran]-4',5'-dicarboxylate (16c): COSY NMR (600 MHz, $CDCl_3$)

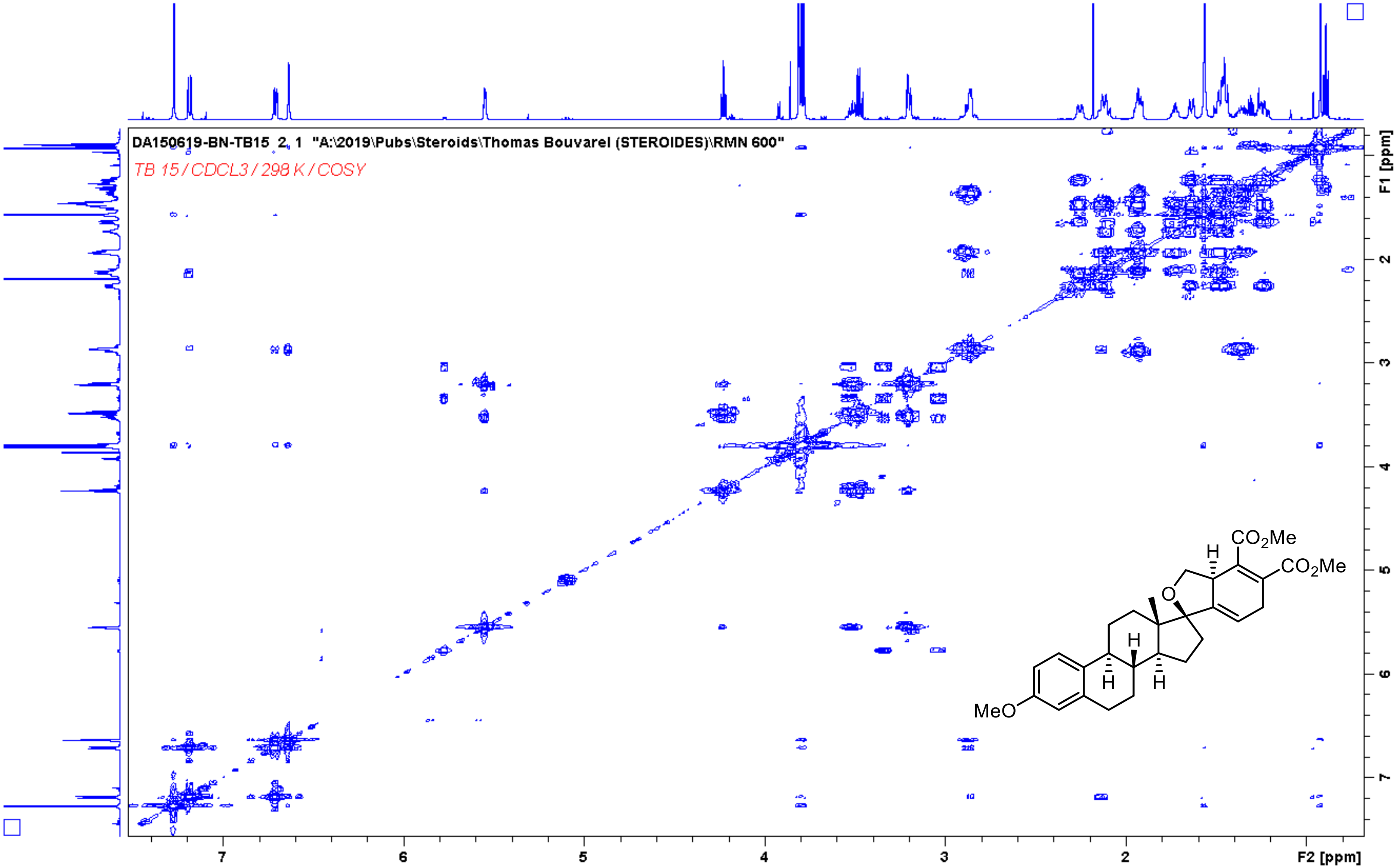

(1'*S*,3a'*R*,8*R*,9*S*,13*S*,14*S*)-Dimethyl 3-methoxy-13-methyl-3a',6,6',7,8,9,11,12,13,14,15,16-dodecahydro-3'*H*-spiro[cyclopenta[*a*]phenanthrene-17,1'-isobenzofuran]-4',5'-dicarboxylate (16c): HMBC NMR (600 MHz, $CDCl_3$)

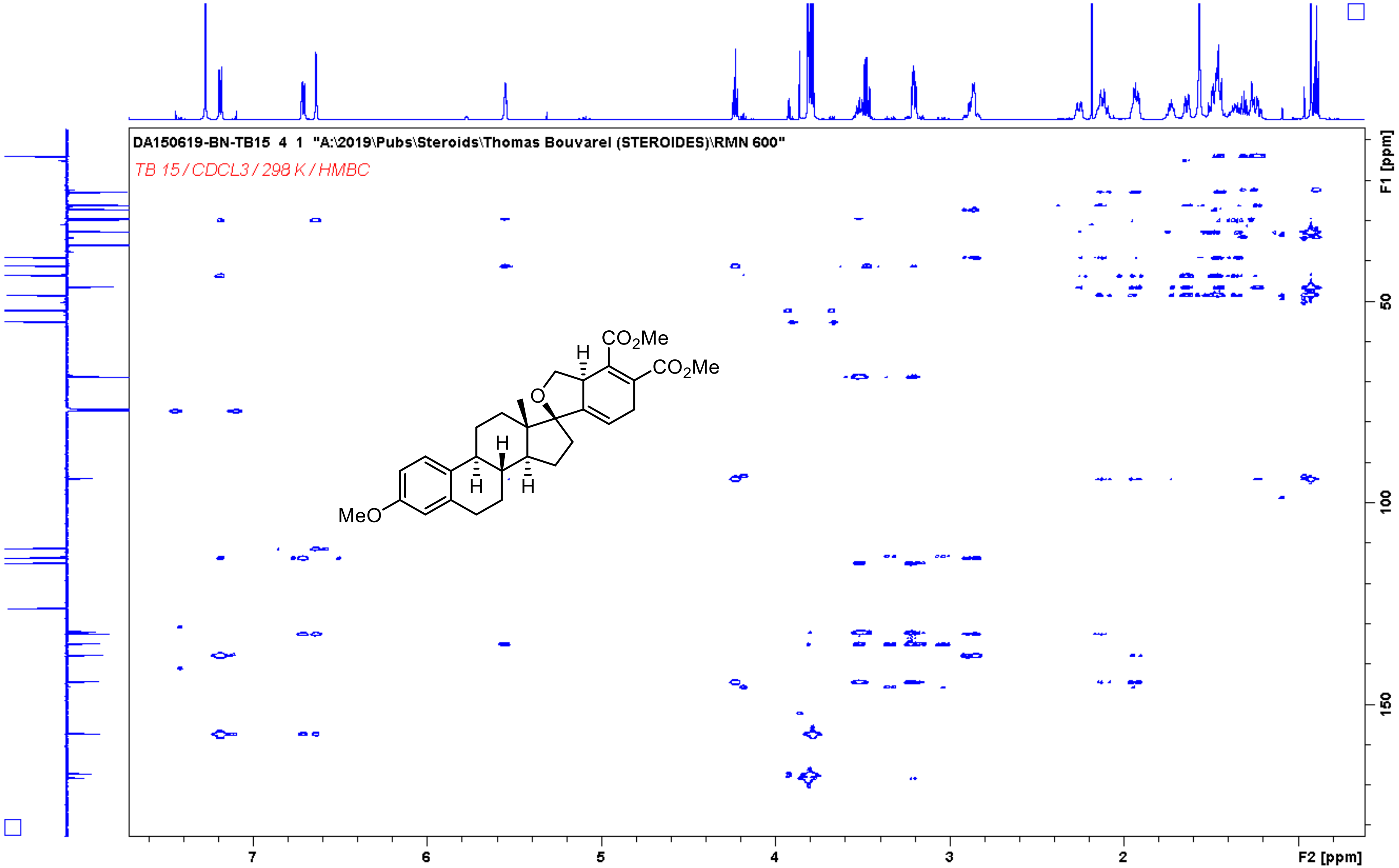

(1'*S*,3a'*R*,8*R*,9*S*,13*S*,14*S*)-Dimethyl 3-methoxy-13-methyl-3a',6,6',7,8,9,11,12,13,14,15,16-dodecahydro-3'*H*-spiro[cyclopenta[*a*]phenanthrene-17,1'-isobenzofuran]-4',5'-dicarboxylate (16c): HSQC NMR (600 MHz, $CDCl_3$)

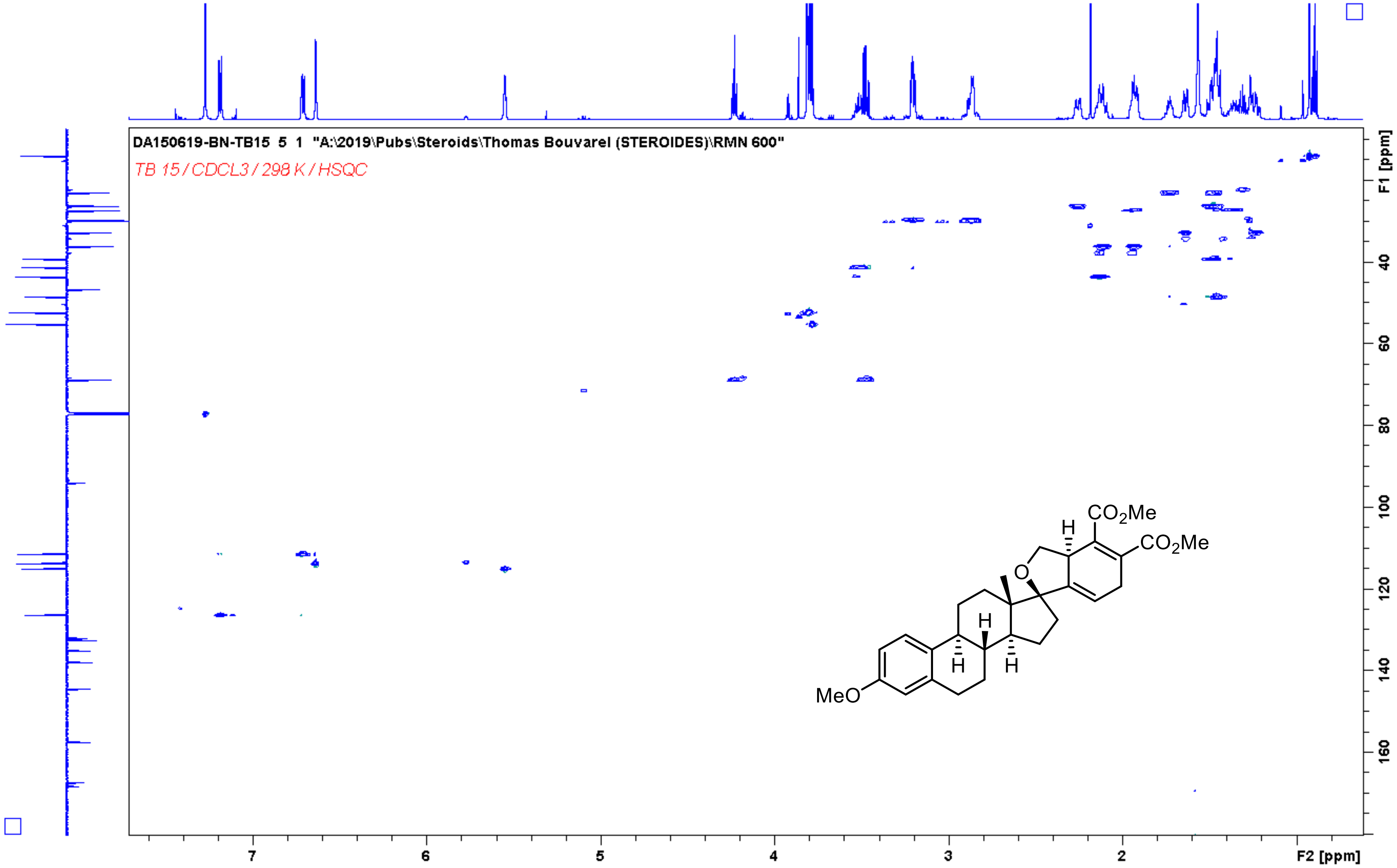

(1'*S*,3a'*R*,8*R*,9*S*,13*S*,14*S*)-Dimethyl 3-methoxy-13-methyl-3a',6,6',7,8,9,11,12,13,14,15,16-dodecahydro-3'*H*-spiro[cyclopenta[*a*]phenanthrene-17,1'-isobenzofuran]-4',5'-dicarboxylate (16c): NOESY NMR (600 MHz, $CDCl_3$)

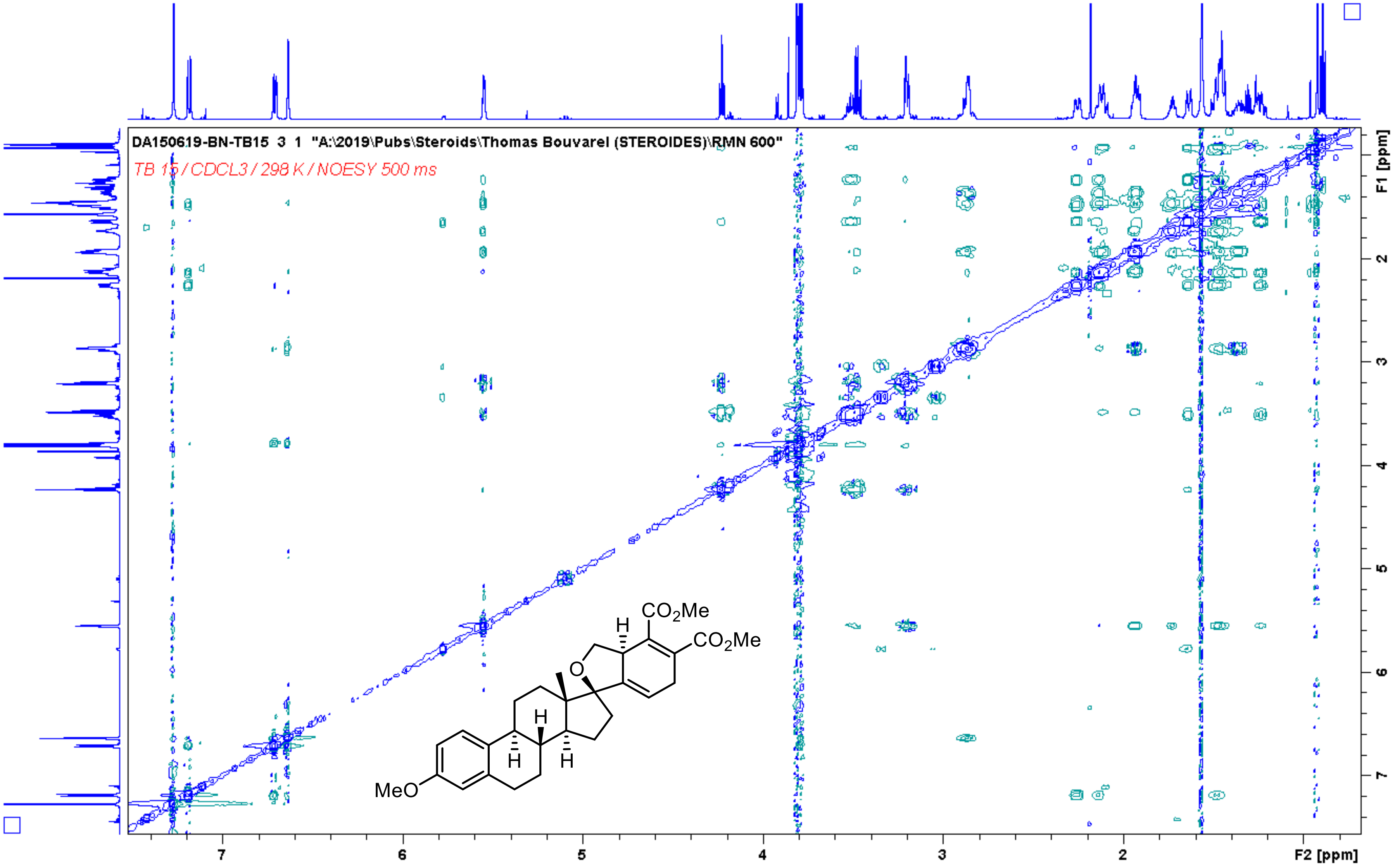

(3'*S*,8*R*,9*S*,9b'*R*,13*S*,14*S*)-3-Methoxy-13-methyl-5',6,7,8,9,9b',11,12,13,14,15,16-dodecahydro-1'*H*-spiro[cyclopenta[*a*]phenanthrene-17,3'-naphtho[1,2-*c*]furan]-6',9'-diol (16d): ^{1}H NMR (400 MHz, $CDCl_3$)

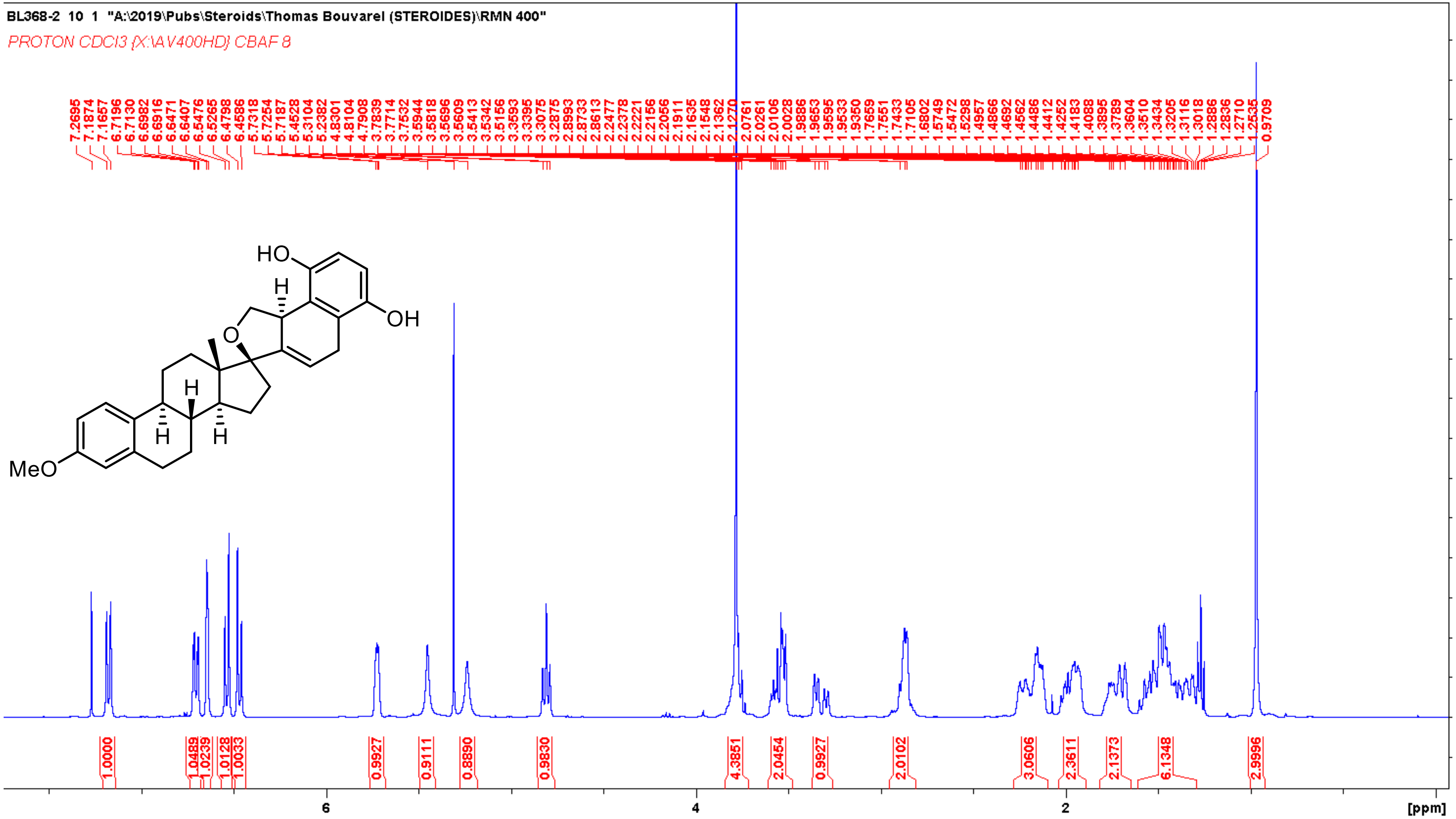

(3'*S*,8*R*,9*S*,9b'*R*,13*S*,14*S*)-3-Methoxy-13-methyl-5',6,7,8,9,9b',11,12,13,14,15,16-dodecahydro-1'*H*-spiro[cyclopenta[*a*]phenanthrene-17,3'-naphtho[1,2-*c*]furan]-6',9'-diol (16d): ^{13}C NMR (150 MHz, $CDCl_3$)

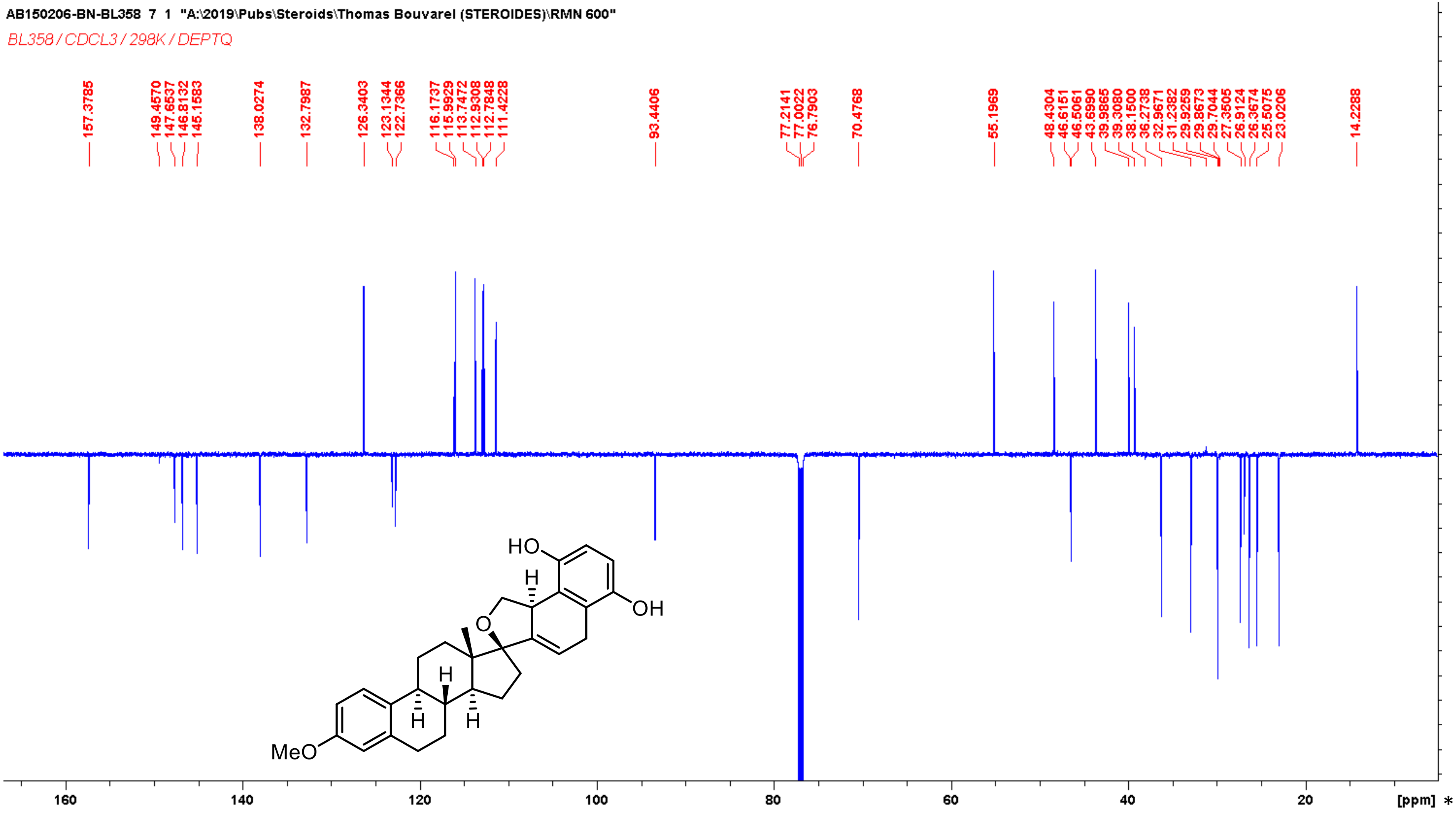

(3'*S*,8*R*,9*S*,9b'*R*,13*S*,14*S*)-3-Methoxy-13-methyl-5',6,7,8,9,9b',11,12,13,14,15,16-dodecahydro-1'*H*-spiro[cyclopenta[*a*]phenanthrene-17,3'-naphtho[1,2-*c*]furan]-6',9'-diol (16d): COSY NMR (600 MHz, $CDCl_3$)

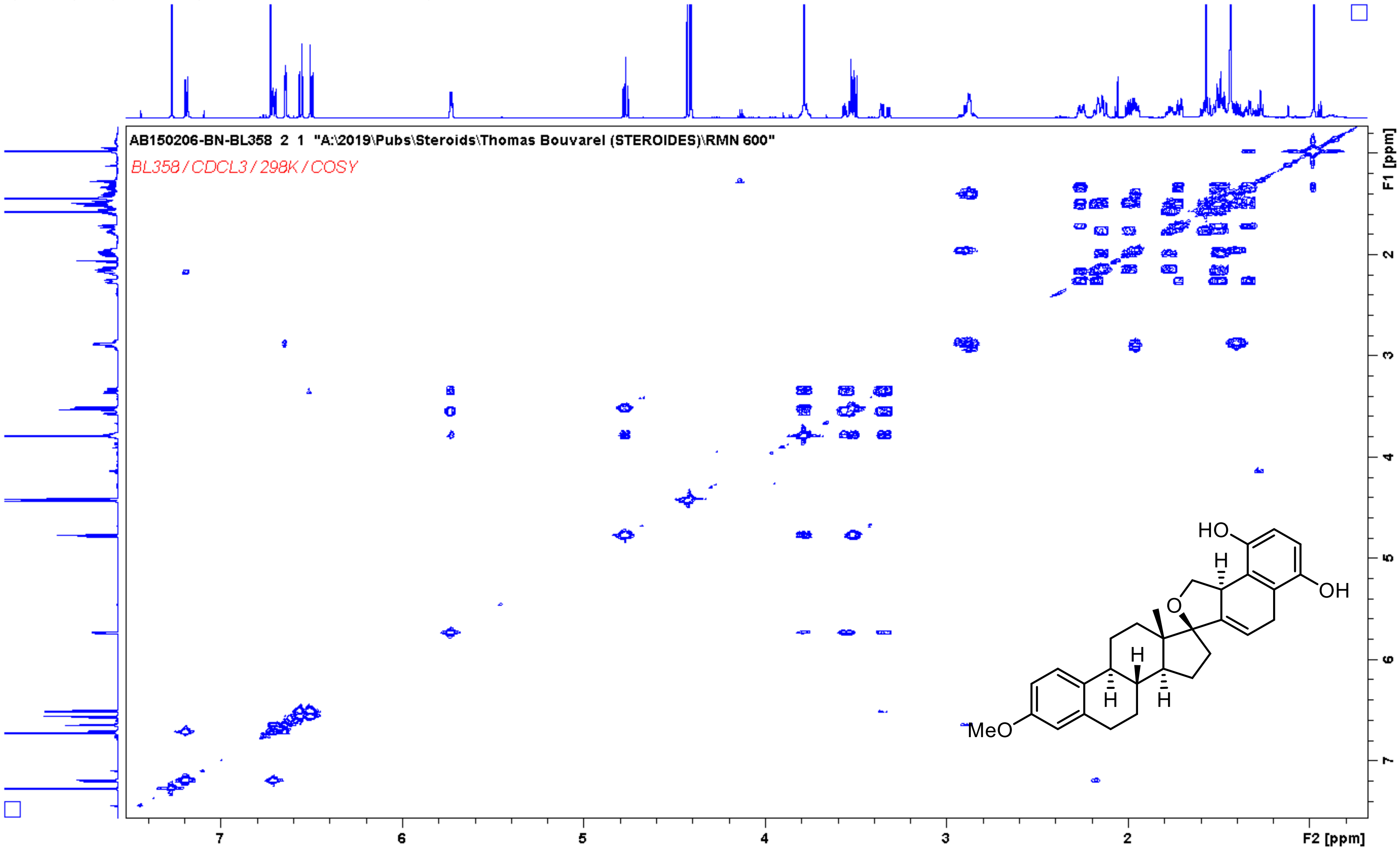

(3'*S*,8*R*,9*S*,9b'*R*,13*S*,14*S*)-3-Methoxy-13-methyl-5',6,7,8,9,9b',11,12,13,14,15,16-dodecahydro-1'*H*-spiro[cyclopenta[*a*]phenanthrene-17,3'-naphtho[1,2-*c*]furan]-6',9'-diol (16d): HMBC NMR (600 MHz, $CDCl_3$)

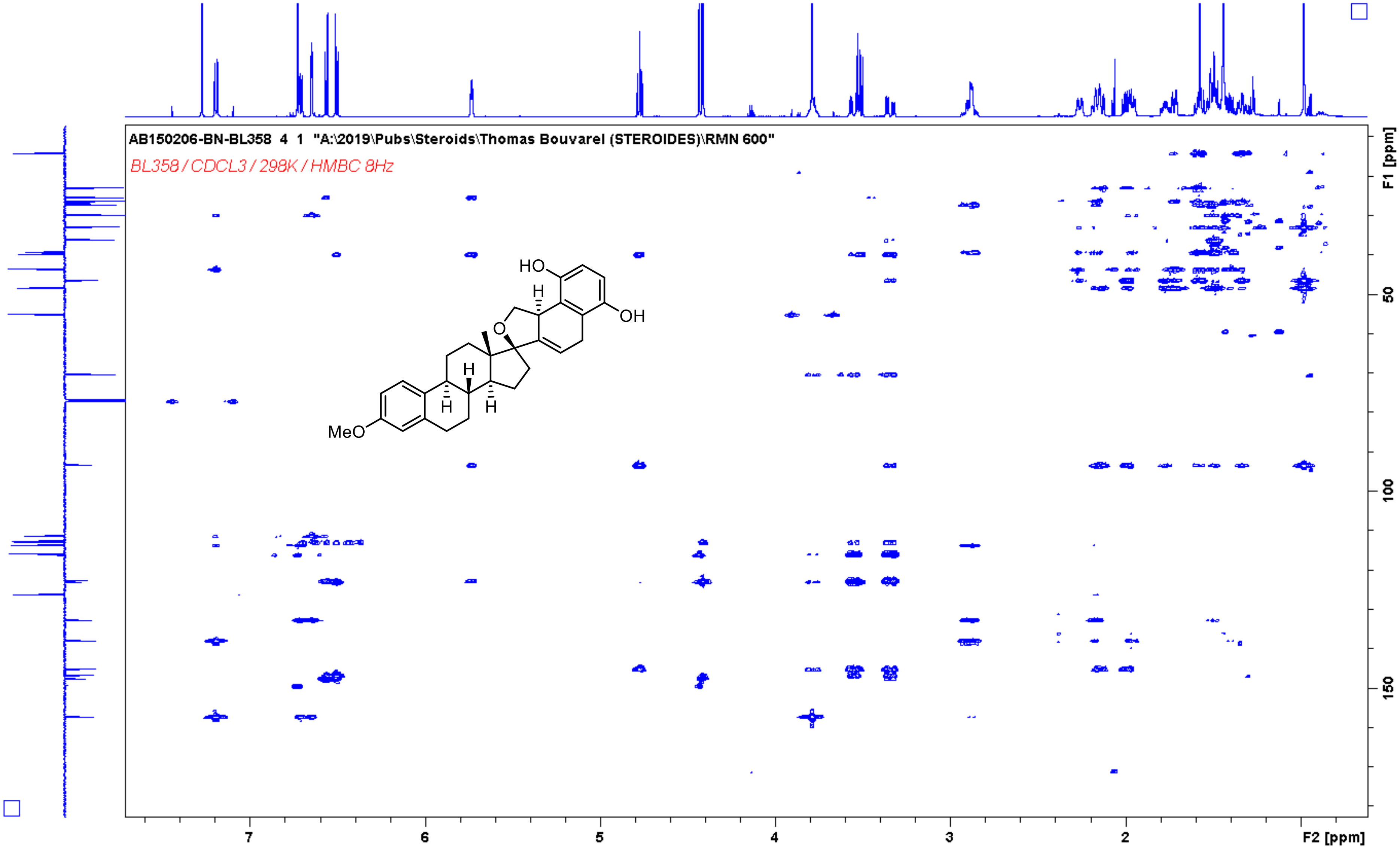

(3'*S*,8*R*,9*S*,9b'*R*,13*S*,14*S*)-3-Methoxy-13-methyl-5',6,7,8,9,9b',11,12,13,14,15,16-dodecahydro-1'*H*-spiro[cyclopenta[*a*]phenanthrene-17,3'-naphtho[1,2-*c*]furan]-6',9'-diol (16d): HSQC NMR (600 MHz, $CDCl_3$)

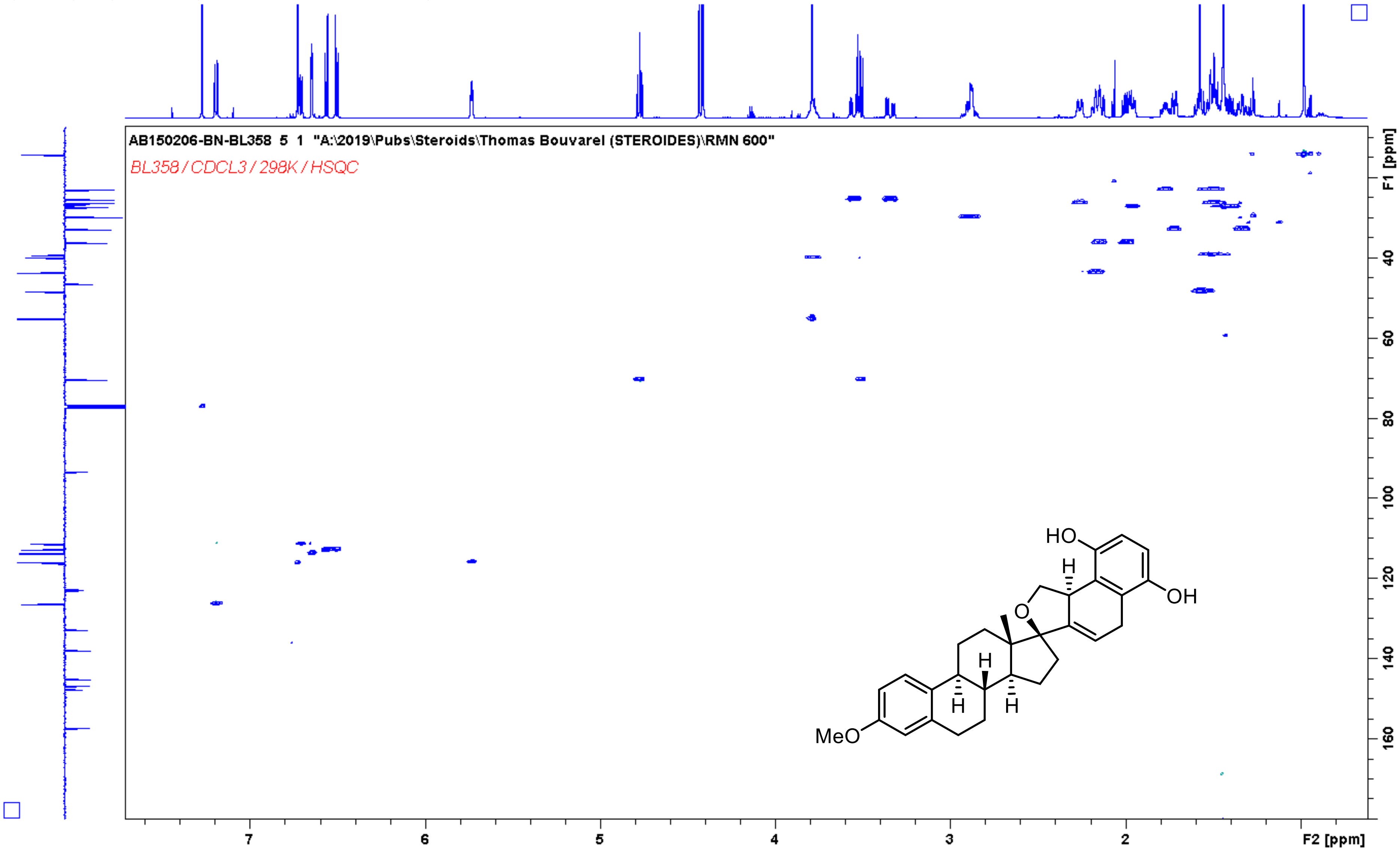

(3'*S*,8*R*,9*S*,9b'*R*,13*S*,14*S*)-3-Methoxy-13-methyl-5',6,7,8,9,9b',11,12,13,14,15,16-dodecahydro-1'*H*-spiro[cyclopenta[*a*]phenanthrene-17,3'-naphtho[1,2-*c*]furan]-6',9'-diol (16d): NOESY NMR (600 MHz, $CDCl_3$)

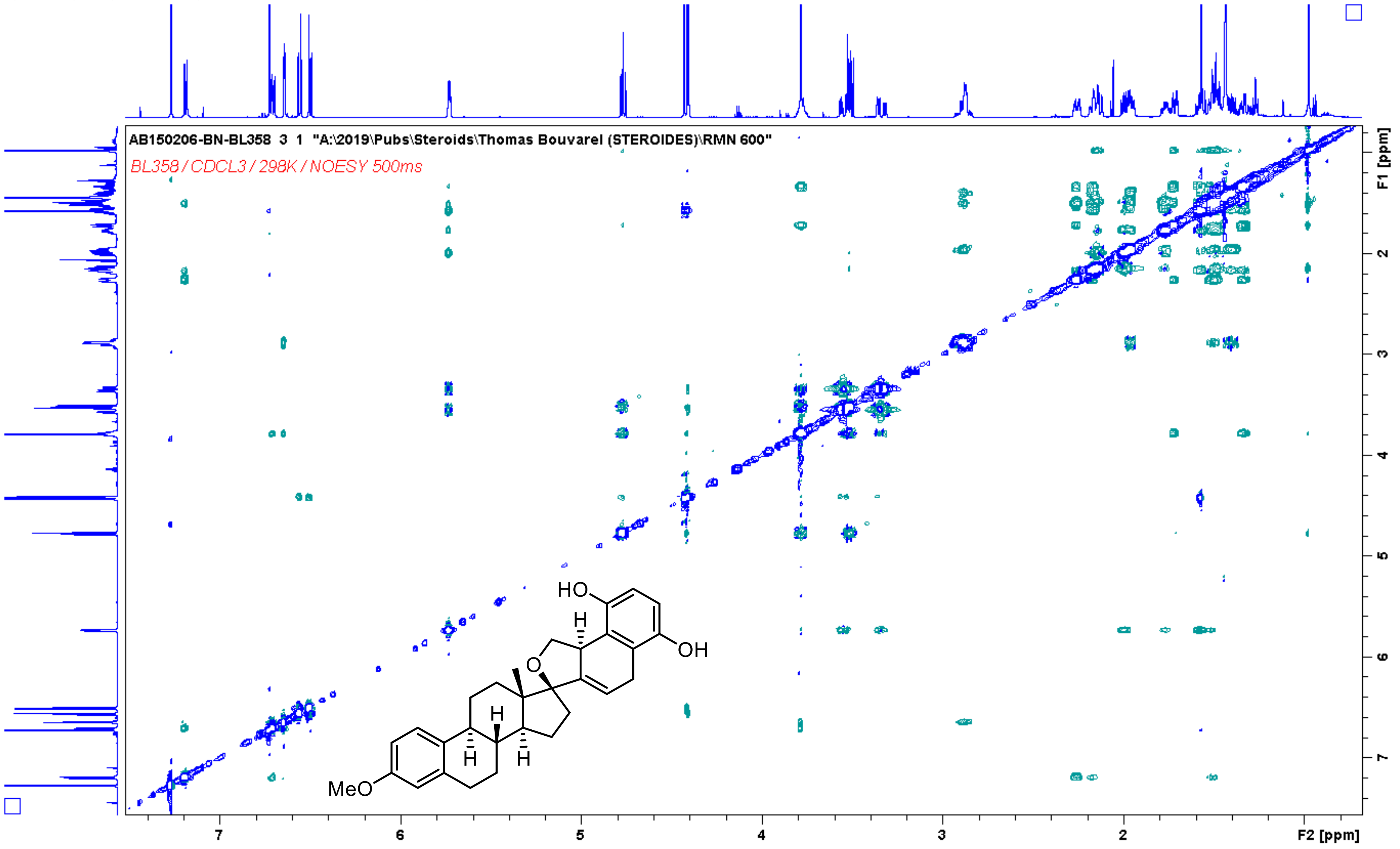

(3*S*,5a*S*,8'*R*,9'*S*,11a*R*,11b*S*,13'*S*,14'*S*)-3'-Methoxy-13'-methyl-5,5a,6',7',8',9',11',12',13',14',15',16'-dodecahydro-1*H*-spiro[anthra[1,2-*c*]furan-3,17'-cyclopenta[*a*]phenanthrene]-6,11-(11a*H*,11b*H*)dione (16e): ^{1}H NMR (600 MHz, $CDCl_3$)

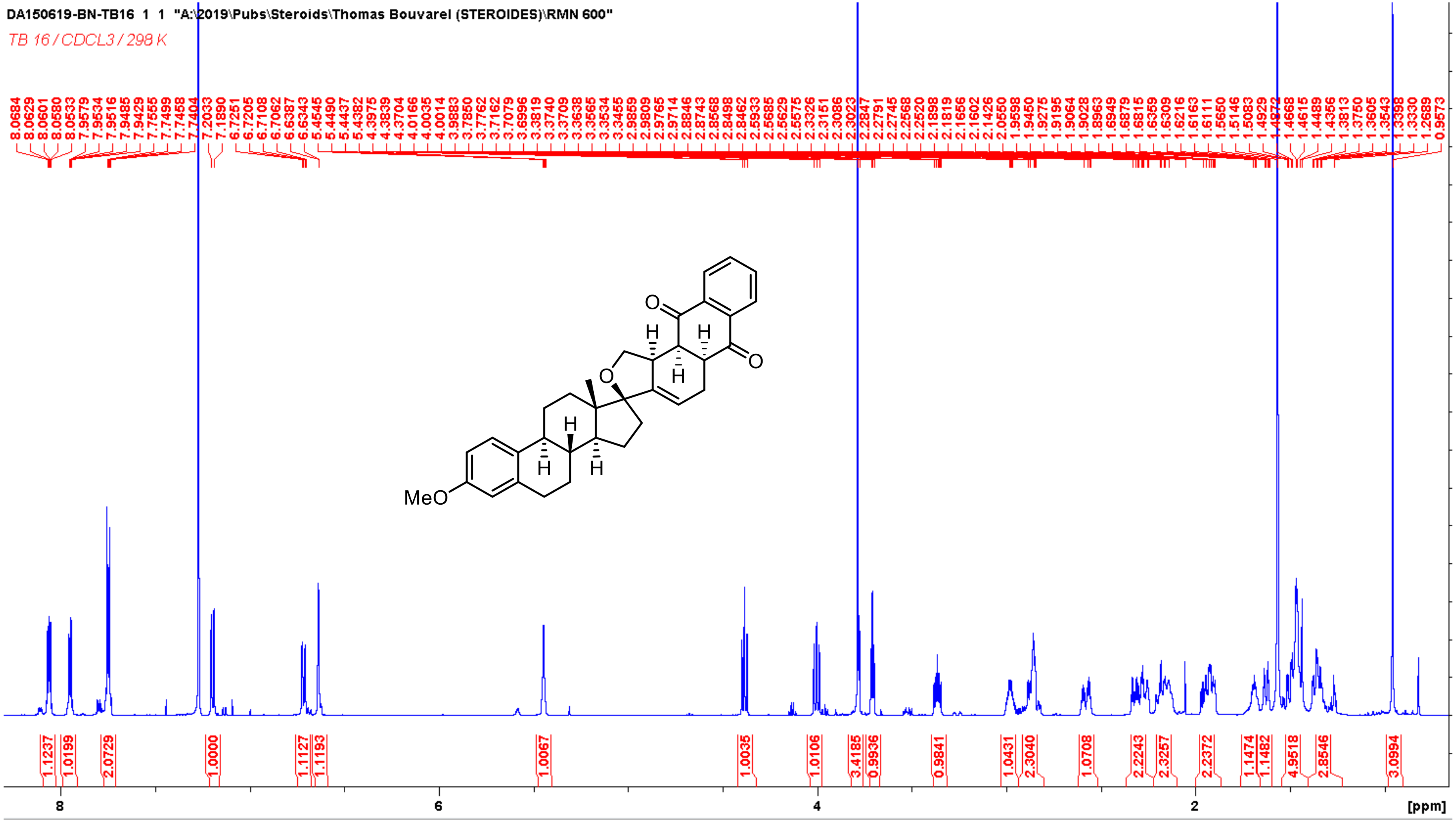

(3*S*,5a*S*,8'*R*,9'*S*,11a*R*,11b*S*,13'*S*,14'*S*)-3'-Methoxy-13'-methyl-5,5a,6',7',8',9',11',12',13',14',15',16'-dodecahydro-1*H*-spiro[anthra[1,2-*c*]furan-3,17'-cyclopenta[*a*]phenanthrene]-6,11-(11a*H*,11b*H*)dione (16e): ^{13}C NMR (150 MHz, $CDCl_3$)

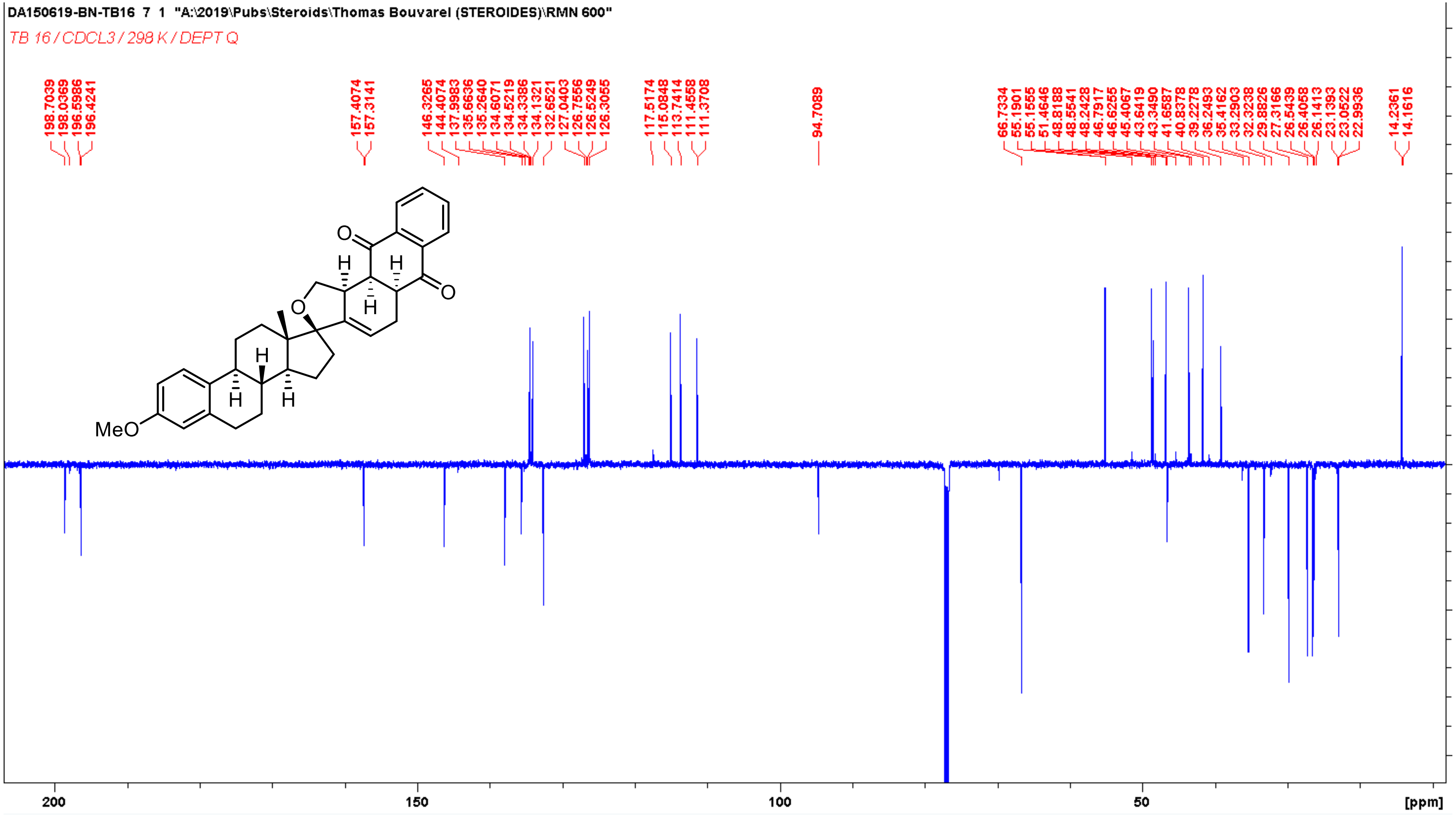

(3*S*,5a*S*,8'*R*,9'S,11a*R*,11b*S*,13'*S*,14'*S*)-3'-Methoxy-13'-methyl-5,5a,6',7',8',9',11',12',13',14',15',16'-dodecahydro-1*H*-spiro[anthra[1,2-*c*]furan-3,17'-cyclopenta[*a*]phenanthrene]-6,11-(11a*H*,11b*H*)dione (16e): COSY NMR (600 MHz, $CDCl_3$)

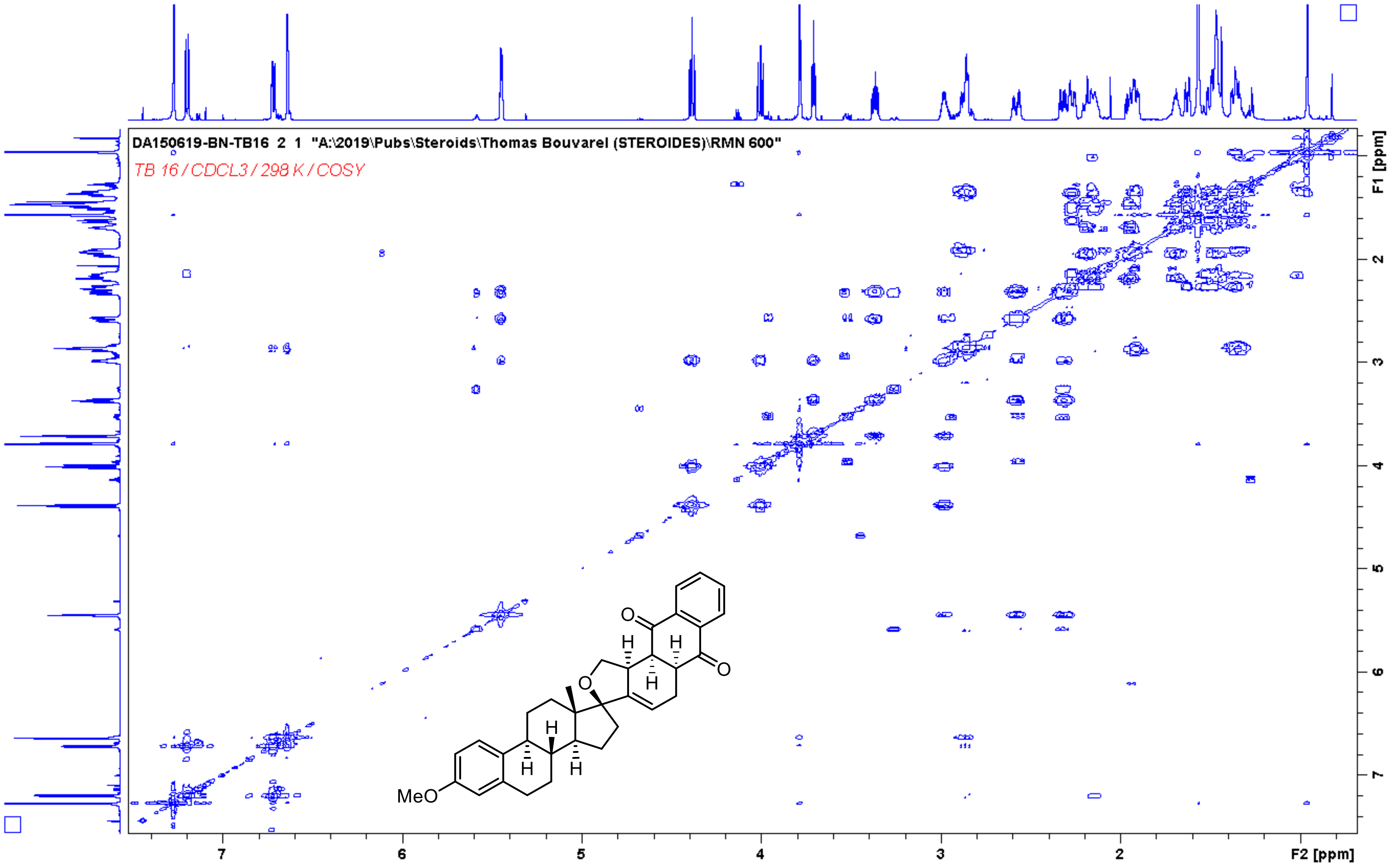

(3*S*,5a*S*,8'*R*,9'*S*,11a*R*,11b*S*,13'*S*,14'*S*)-3'-Methoxy-13'-methyl-5,5a,6',7',8',9',11',12',13',14',15',16'-dodecahydro-1*H*-spiro[anthra[1,2-*c*]furan-3,17'-cyclopenta[*a*]phenanthrene]-6,11-(11aH,11b*H*)dione (16e): HMBC NMR (600 MHz, $CDCl_3$)

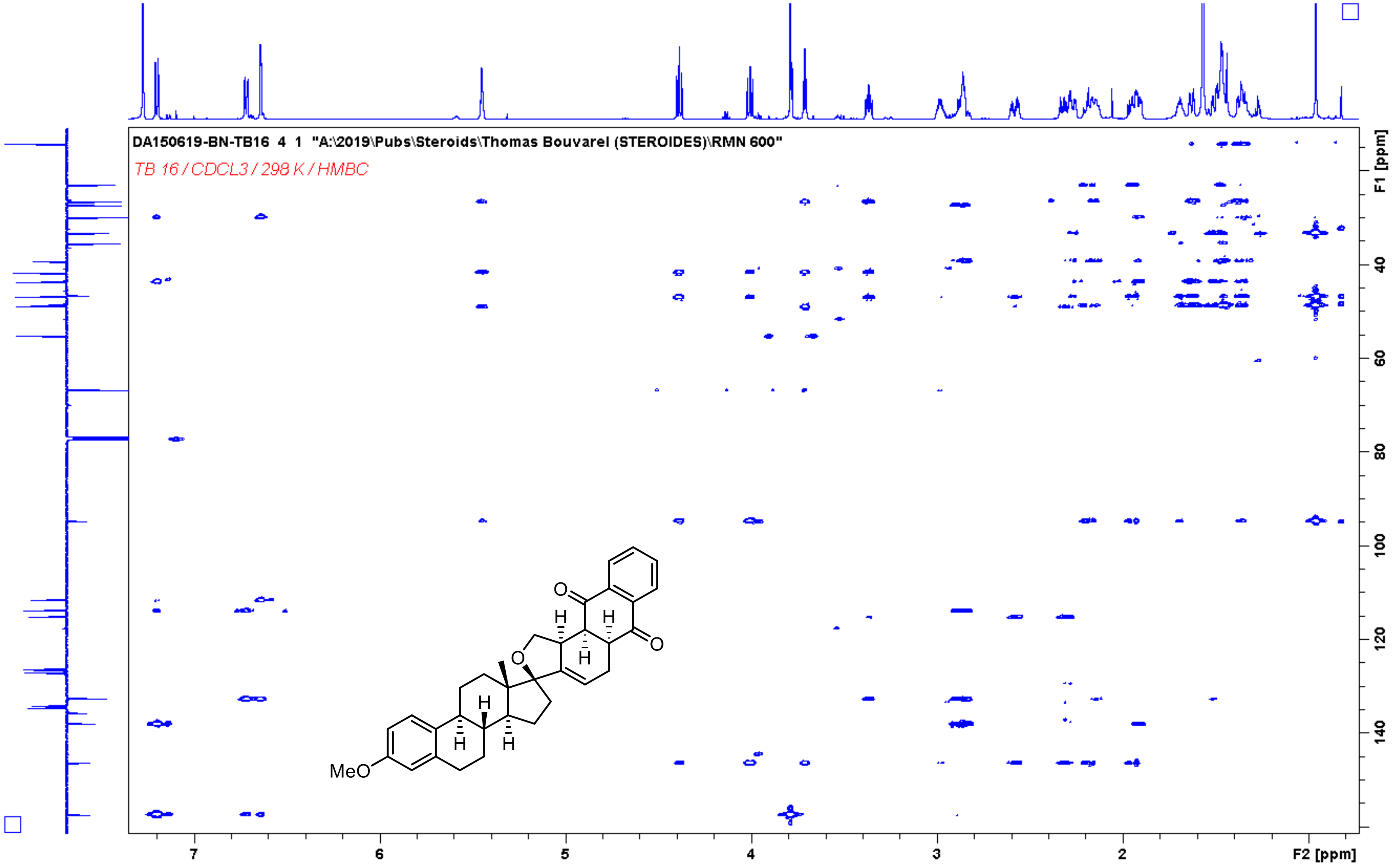

(3*S*,5a*S*,8'i,9'*S*,11a*R*,11b*S*,13'*S*,14'i)-3'-Methoxy-13'-methyl-5,5a,6',7',8',9',11',12',13',14',15',16'-dodecahydro-1*H*-spiro[anthra[1,2-*c*]furan-3,17'-cyclopenta[*a*]phenanthrene]-6,11-(11a*H*,11b*H*)dione (16e): HSQC NMR (600 MHz, $CDCl_3$)

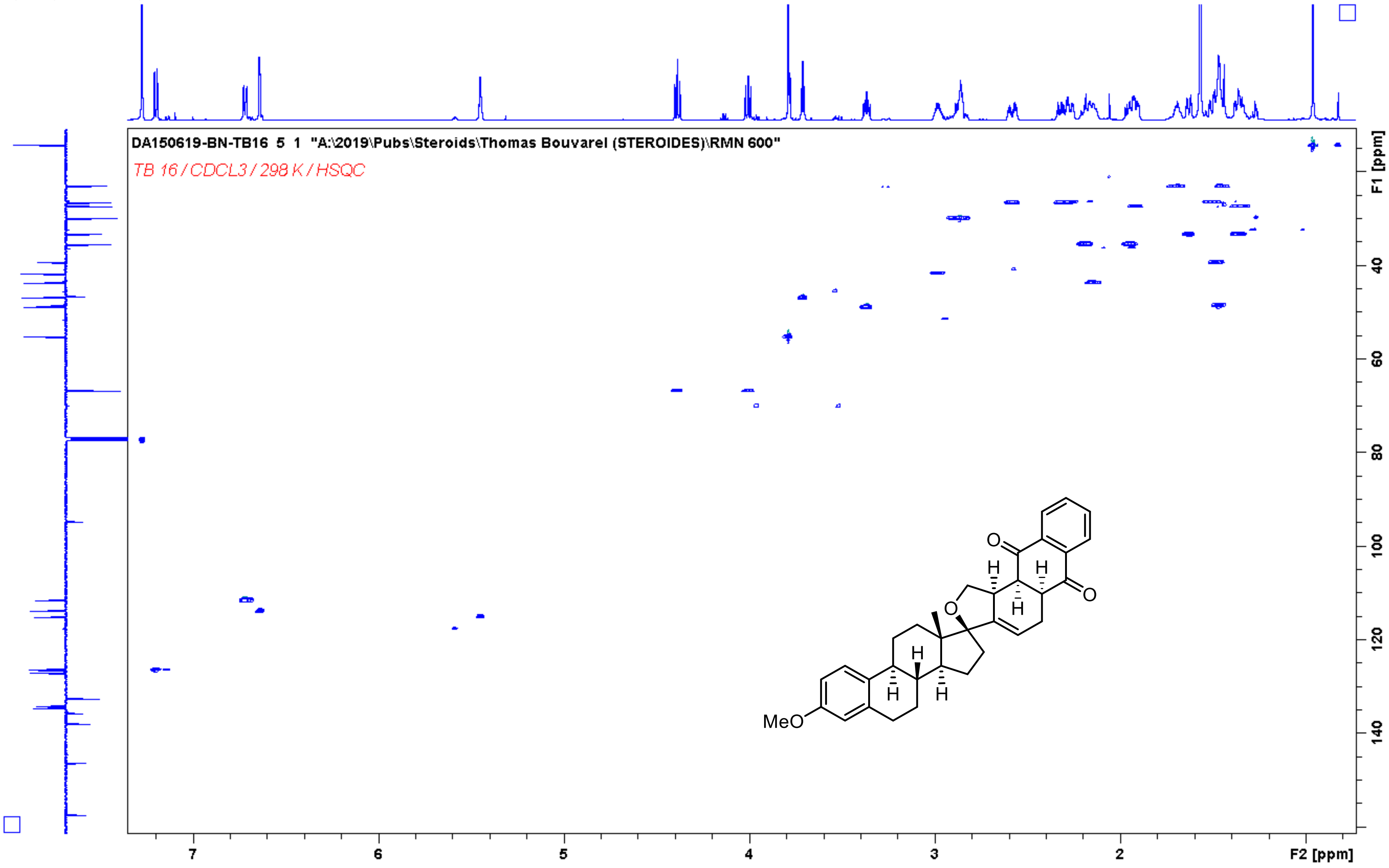

(3*S*,5a*S*,8'*R*,9'*S*,11a*R*,11b*S*,13'*S*,14'*S*)-3'-Methoxy-13'-methyl-5,5a,6',7',8',9',11',12',13',14',15',16'-dodecahydro-1*H*-spiro[anthra[1,2-*c*]furan-3,17'-cyclopenta[*a*]phenanthrene]-6,11-(11a*H*,11b*H*)dione (16e): NOESY NMR (600 MHz, $CDCl_3$)

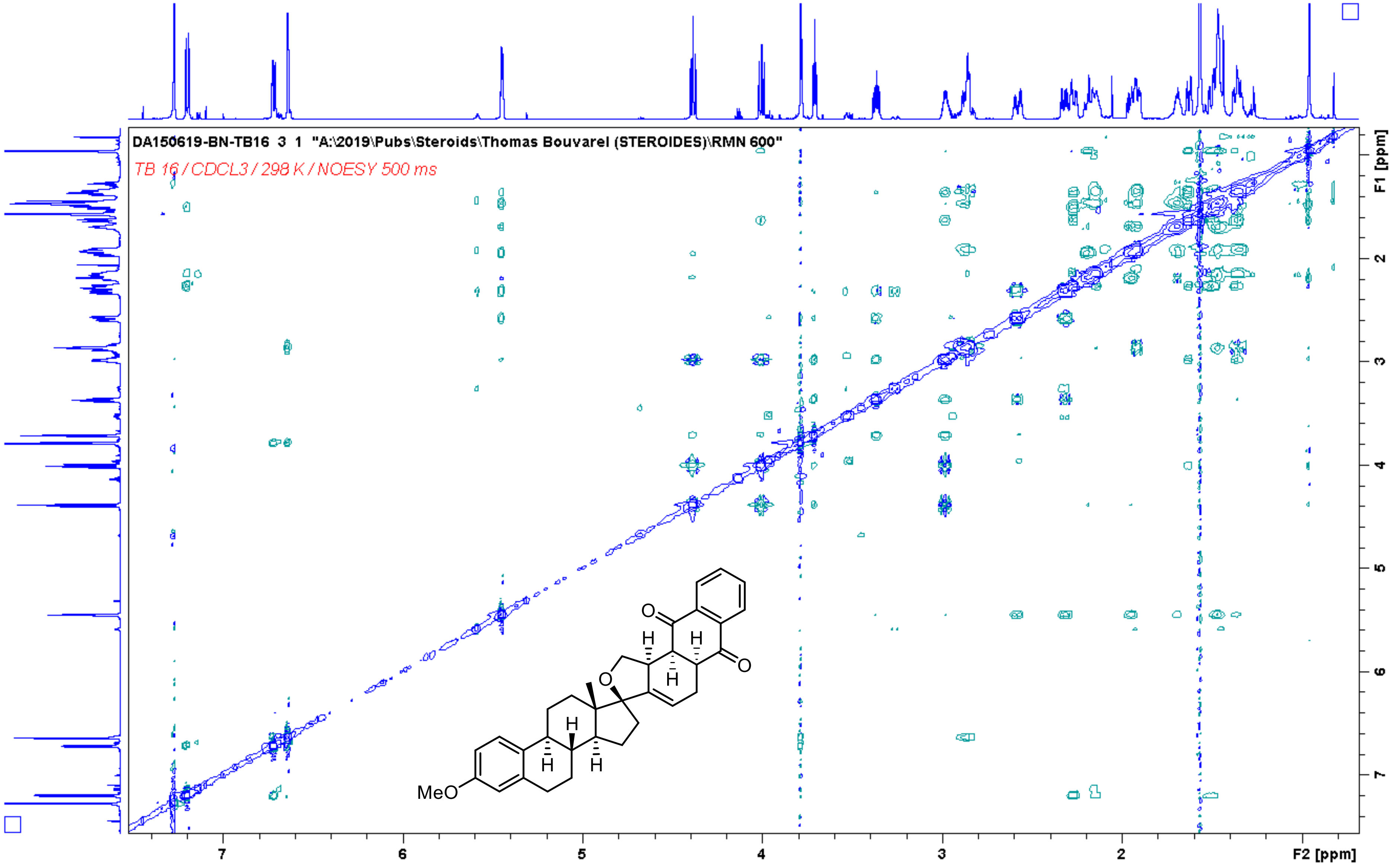

(3a'*S*,6'*S*,8*R*,9*S*,10a'*S*,10b'*R*,13*S*,14*S*)-3-Methoxy-13-methyl-2'-phenyl-3a',4',6,7,8,8',9,9',10',10a',11,12,13,14,15,16-hexadecahydrospiro[cyclopenta[*a*]phenanthrene-17,6'-oxepino[4,3-*e*]isoindole]-1',3'(2'H,10b'*H*)-dione (16f): ^{1}H NMR (600 MHz, CDCl$_3$)

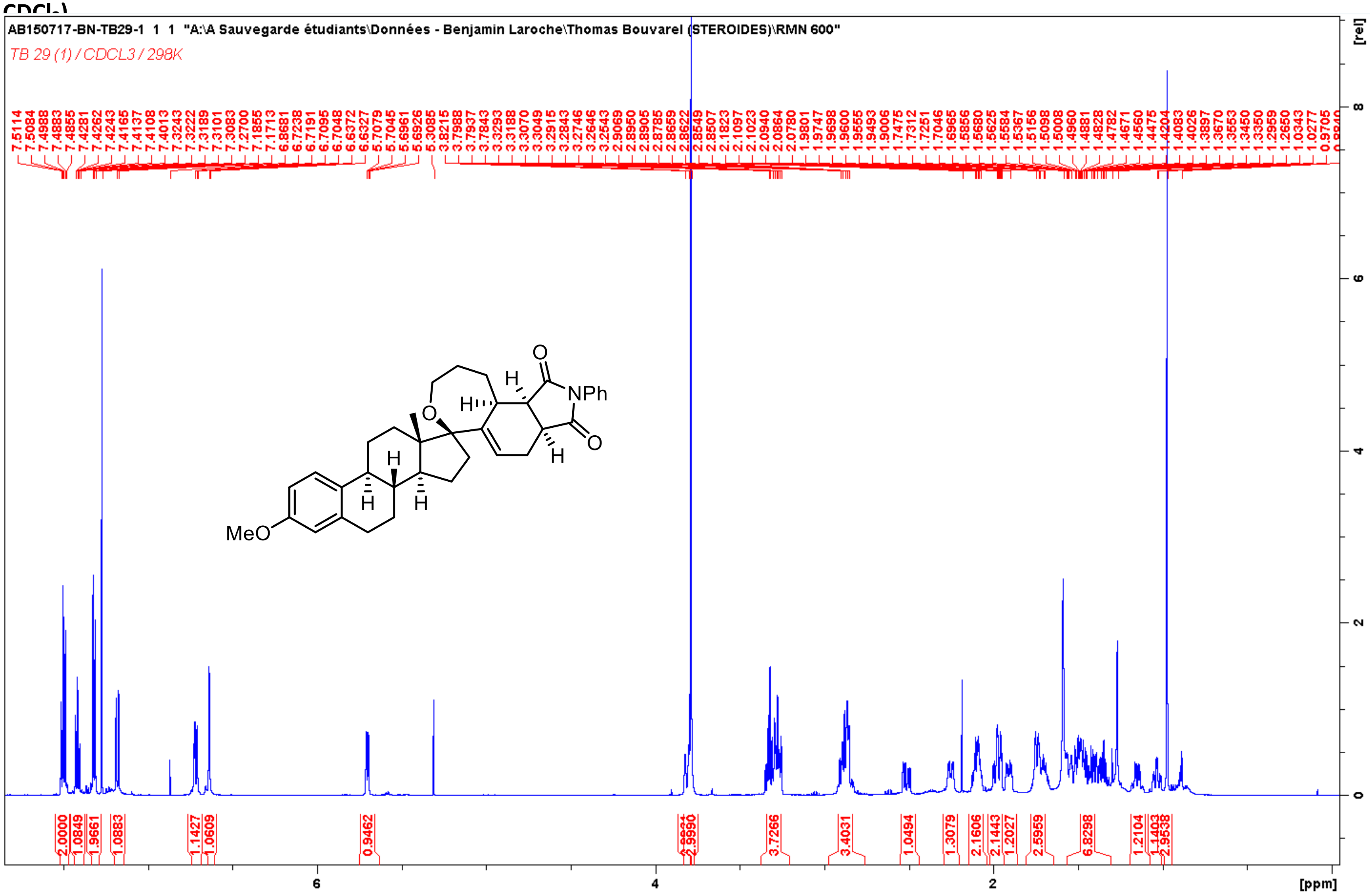

(3a'*S*,6'*S*,8*R*,9*S*,10a'*S*,10b'*R*,13*S*,14*S*)-3-Methoxy-13-methyl-2'-phenyl-3a',4',6,7,8,8',9,9',10',10a',11,12,13,14,15,16-hexadecahydrospiro[cyclopenta[*a*]phenanthrene-17,6'-oxepino[4,3-*e*]isoindole]-1',3'-(2'*H*,10b'*H*)dione (16f): ^{13}C NMR (150 MHz, $CDCl_3$)

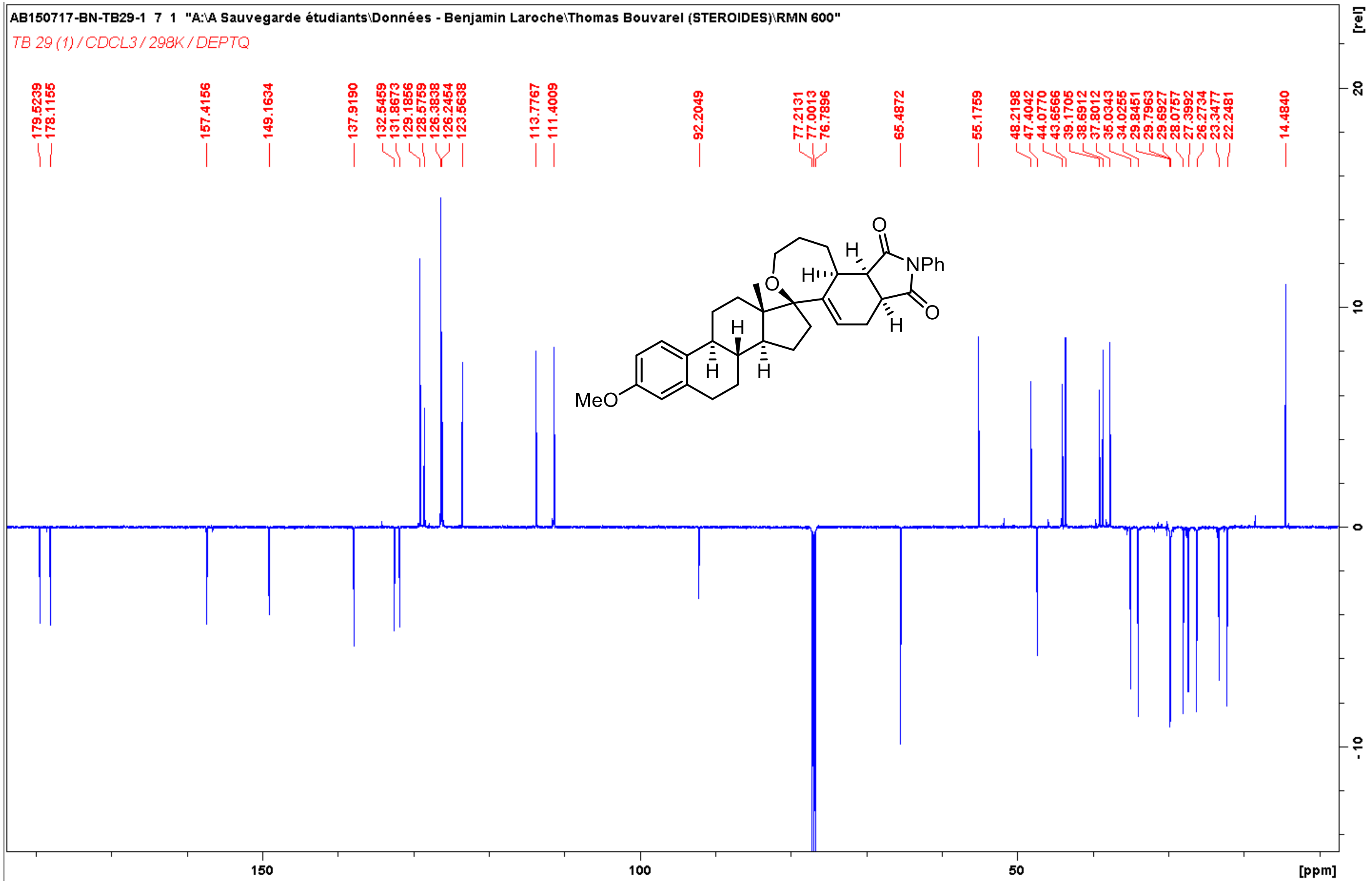

(3a'*S*,6'*S*,8*R*,9*S*,10a'*S*,10*b*'R,13*S*,14*S*)-3-Methoxy-13-methyl-2'-phenyl-3a',4',6,7,8,8',9,9',10',10a',11,12,13,14,15,16-hexadecahydrospiro[cyclopenta[*a*]phenanthrene-17,6'-oxepino[4,3-*e*]isoindole]-1',3'-(2'*H*,10b'*H*)dione (16f): COSY NMR (600 MHz, $CDCl_3$)

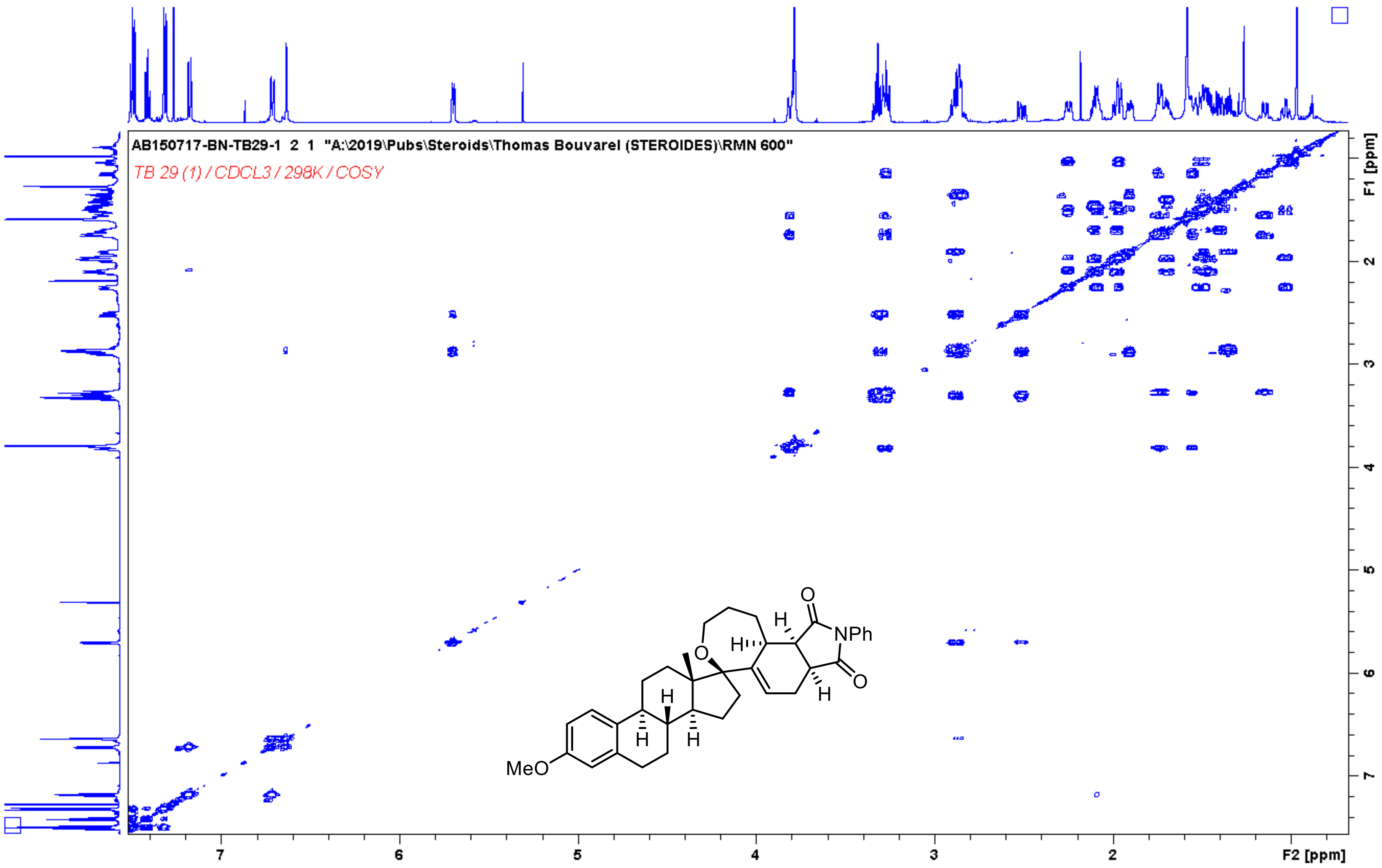

(3a'*S*,6'*S*,8*R*,9*S*,10a'*S*,10b'*R*,13*S*,14*S*)-3-Methoxy-13-methyl-2'-phenyl-3a',4',6,7,8,8',9,9',10',10a',11,12,13,14,15,16-hexadecahydrospiro[cyclopenta[*a*]phenanthrene-17,6'-oxepino[4,3-*e*]isoindole]-1',3'-(2'*H*,10b'*H*)dione (16f): HMBC NMR (600 MHz, $CDCl_3$)

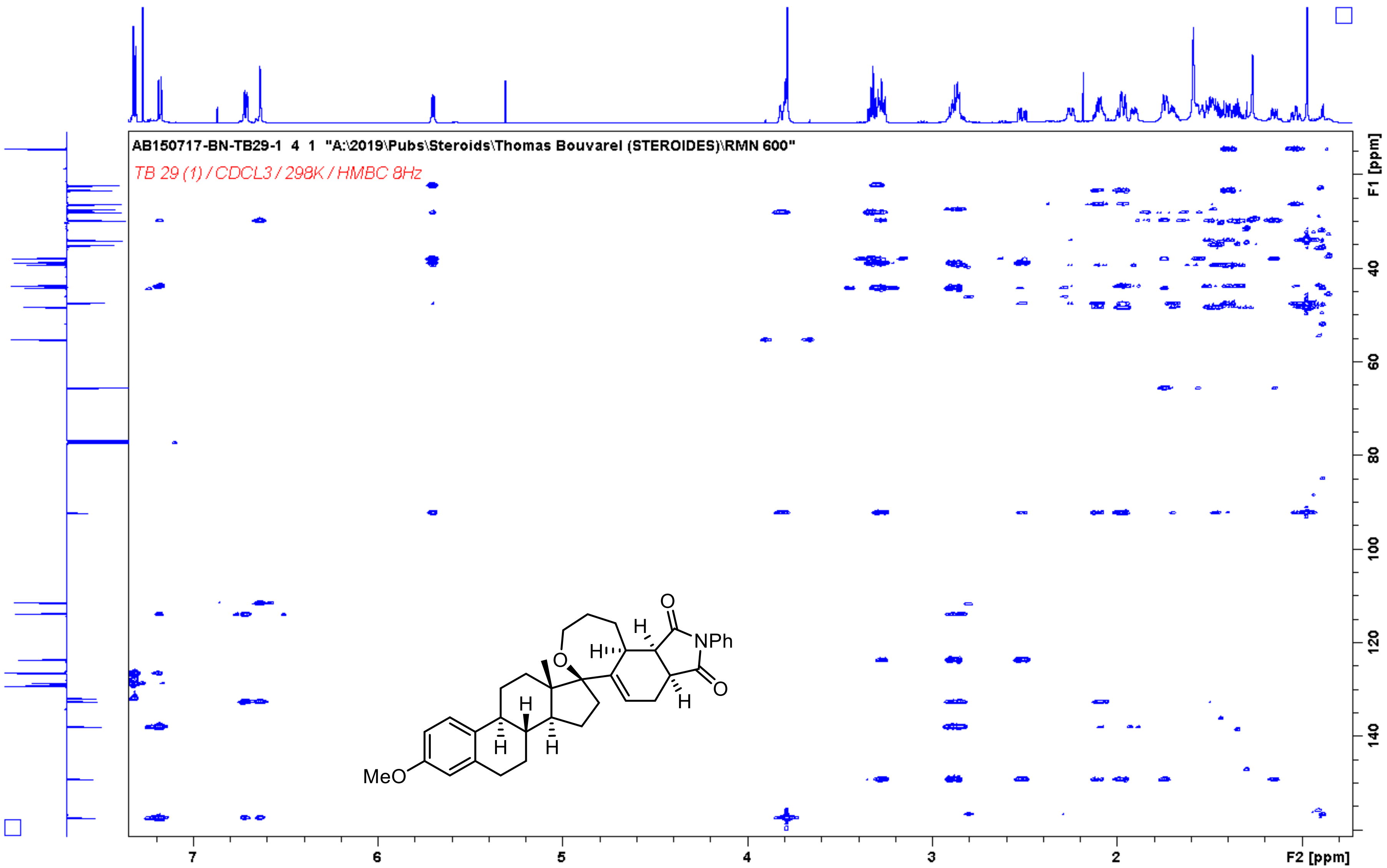

(3a'*S*,6'*S*,8*R*,9*S*,10a'*S*,10b'*R*,13*S*,14*S*)-3-Methoxy-13-methyl-2'-phenyl-3a',4',6,7,8,8',9,9',10',10a',11,12,13,14,15,16-hexadecahydrospiro[cyclopenta[*a*]phenanthrene-17,6'-oxepino[4,3-*e*]isoindole]-1',3'-(2'*H*,10b'*H*)dione (16f): HSQC NMR (600 MHz, $CDCl_3$)

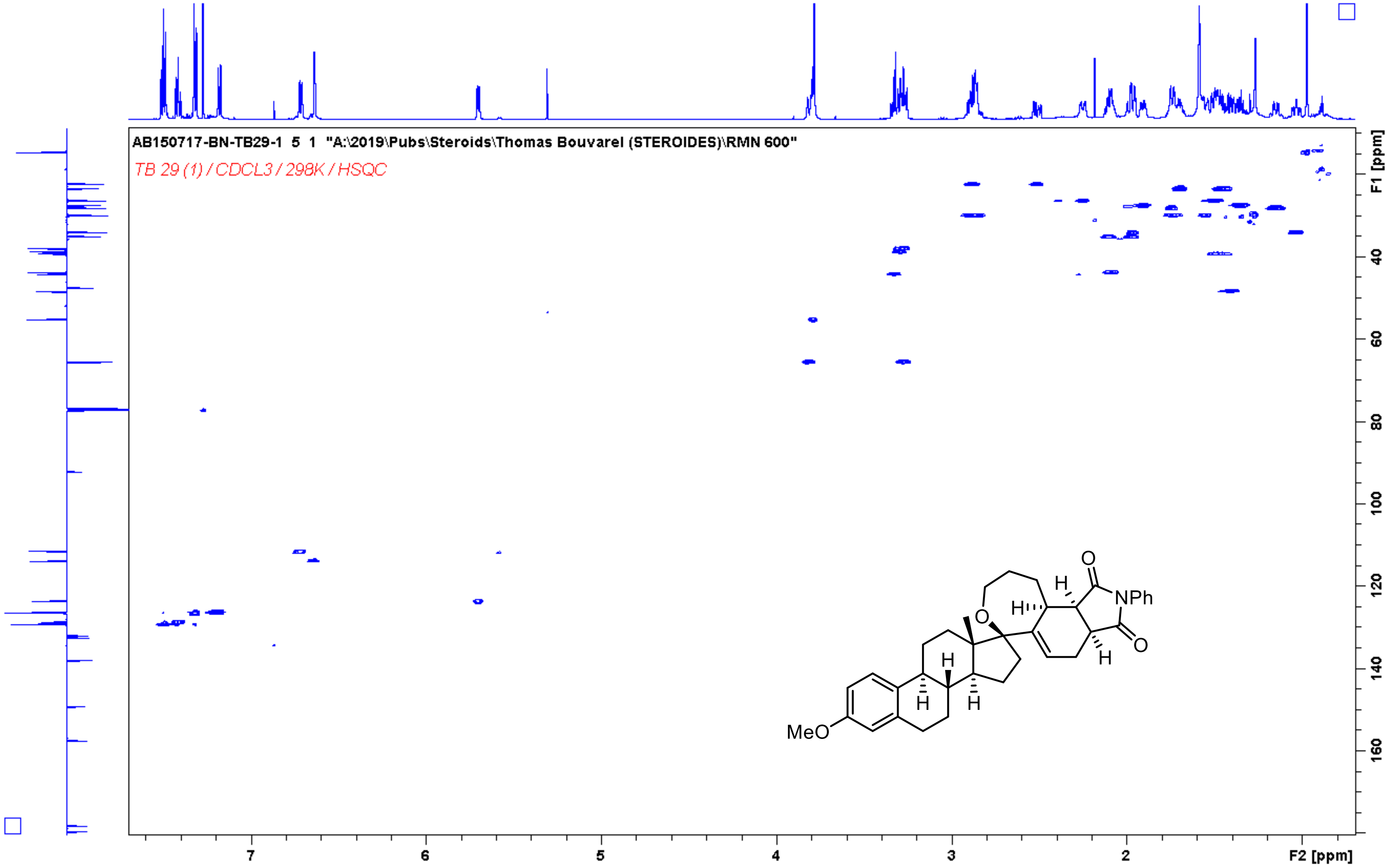

(3a'*S*,6'*S*,8*R*,9*S*,10a'*S*,10b'*R*,13*S*,14*S*)-3-Methoxy-13-methyl-2'-phenyl-3a',4',6,7,8,8',9,9',10',10a',11,12,13,14,15,16-hexadecahydrospiro[cyclopenta[*a*]phenanthrene-17,6'-oxepino[4,3-*e*]isoindole]-1',3'-(2'*H*,10b'*H*)dione (16f): NOESY NMR (600 MHz, $CDCl_3$)

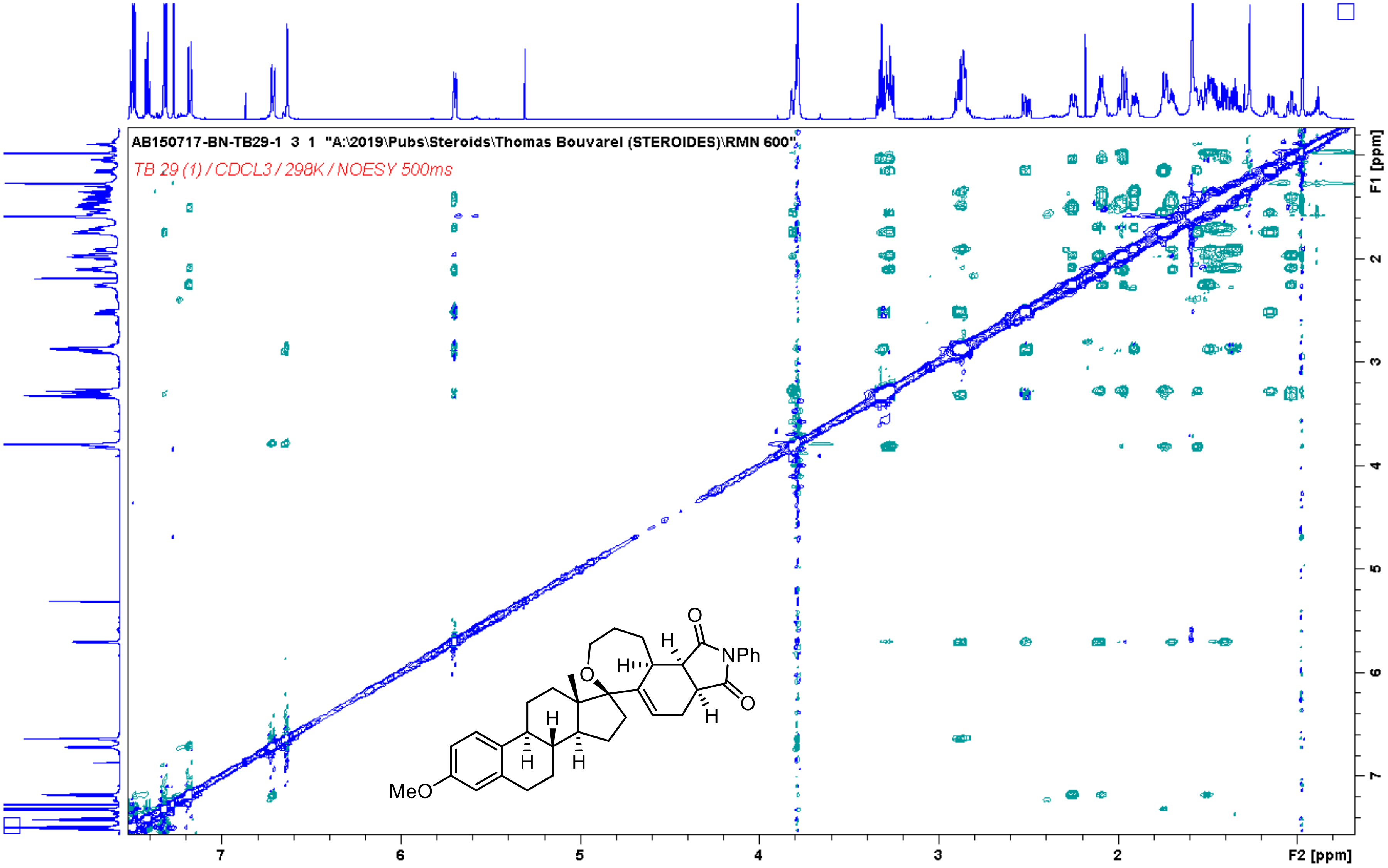

(3'*S*,5a'*S*,8*R*,8a'*R*,8b'*S*,9*S*,10*R*,13*S*,14*S*)-13-Methyl-7'-phenyl-1,1',2,3,5',5a',6,7,8,8b',9,10,11,12,13,14,15,16-octadecahydrospiro[cyclopenta[*a*]phenanthrene-17,3'-furo[3,4-*e*]isoindole]-6',8'-(7'*H*,8a'*H*)dione (17a): ^{1}H NMR (600 MHz, $CDCl_3$)

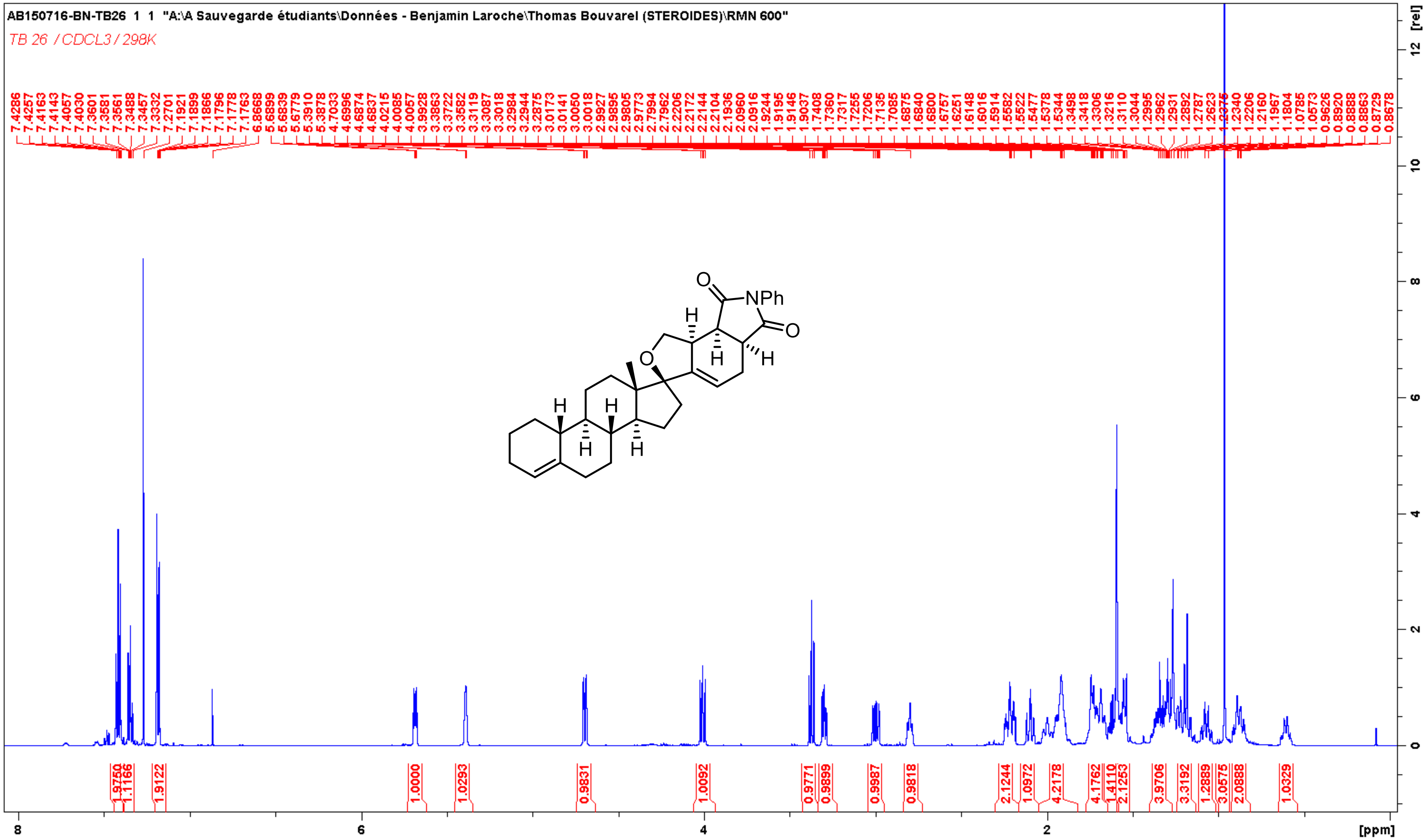

(3'*S*,5a'*S*,8*R*,8a'*R*,8b'*S*,9*S*,10*R*,13*S*,14*S*)-13-Methyl-7'-phenyl-1,1',2,3,5',5a',6,7,8,8b',9,10,11,12,13,14,15,16-octadecahydrospiro[cyclopenta[*a*]phenanthrene-17,3'-furo[3,4-*e*]isoindole]-6',8'-(7'*H*,8a'*H*)dione (17a): ^{13}C NMR (150 MHz, CDCl$_3$)

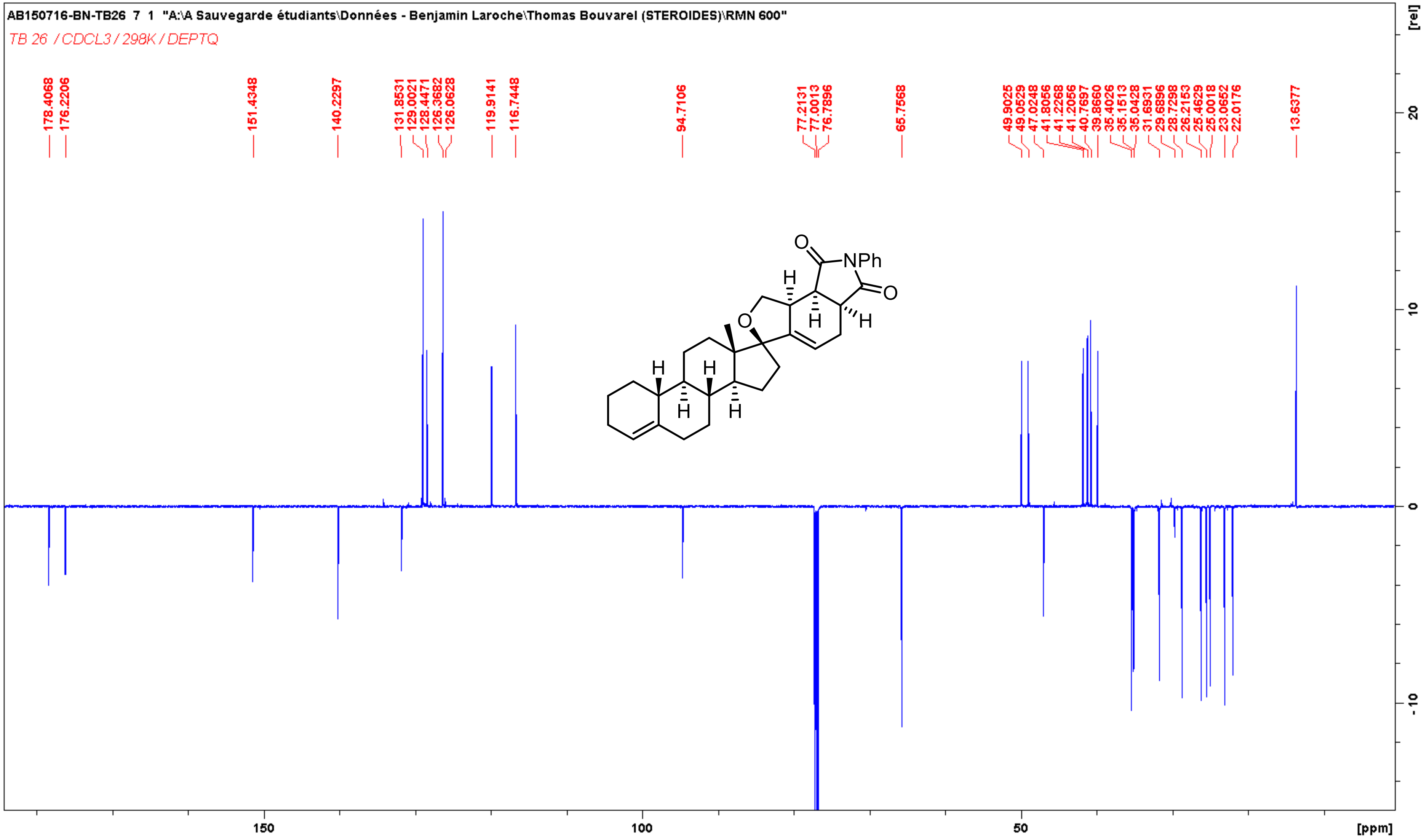

(3'*S*,5a'*S*,8*R*,8a'*R*,8b'*S*,9*S*,10*R*,13*S*,14*S*)-13-Methyl-7'-phenyl-1,1',2,3,5',5a',6,7,8,8b',9,10,11,12,13,14,15,16-octadecahydrospiro[cyclopenta[*a*]phenanthrene-17,3'-furo[3,4-*e*]isoindole]-6',8'-(7'*H*,8a'*H*)dione (17a): COSY NMR (600 MHz, $CDCl_3$)

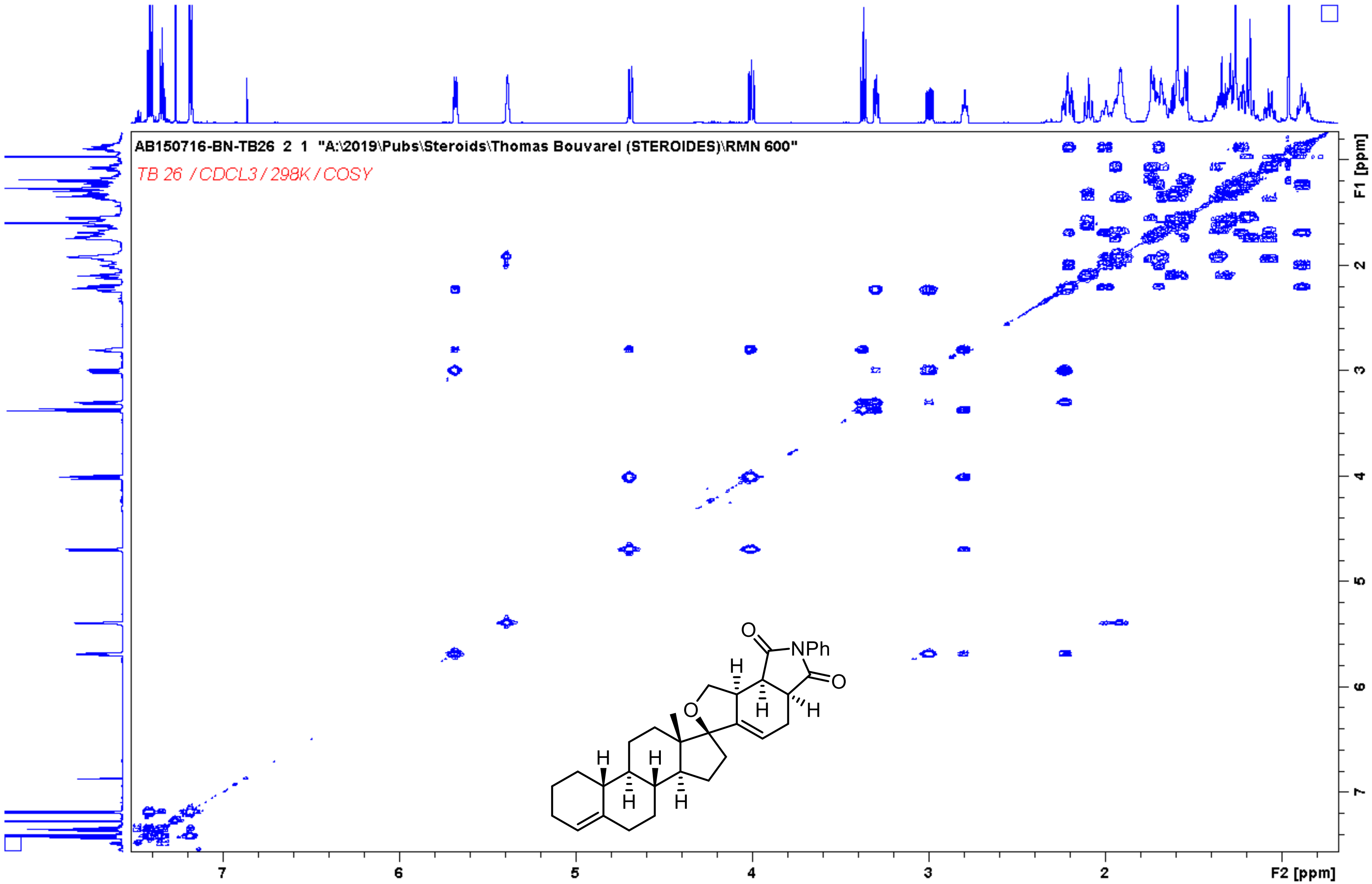

(3'*S*,5a'*S*,8*R*,8a'*R*,8b'*S*,9*S*,10*R*,13*S*,14*S*)-13-Methyl-7'-phenyl-1,1',2,3,5',5a',6,7,8,8b',9,10,11,12,13,14,15,16-octadecahydrospiro[cyclopenta[*a*]phenanthrene-17,3'-furo[3,4-*e*]isoindole]-6',8'(-7'*H*,8a'*H*)dione (17a): HMBC NMR (600 MHz, $CDCl_3$)

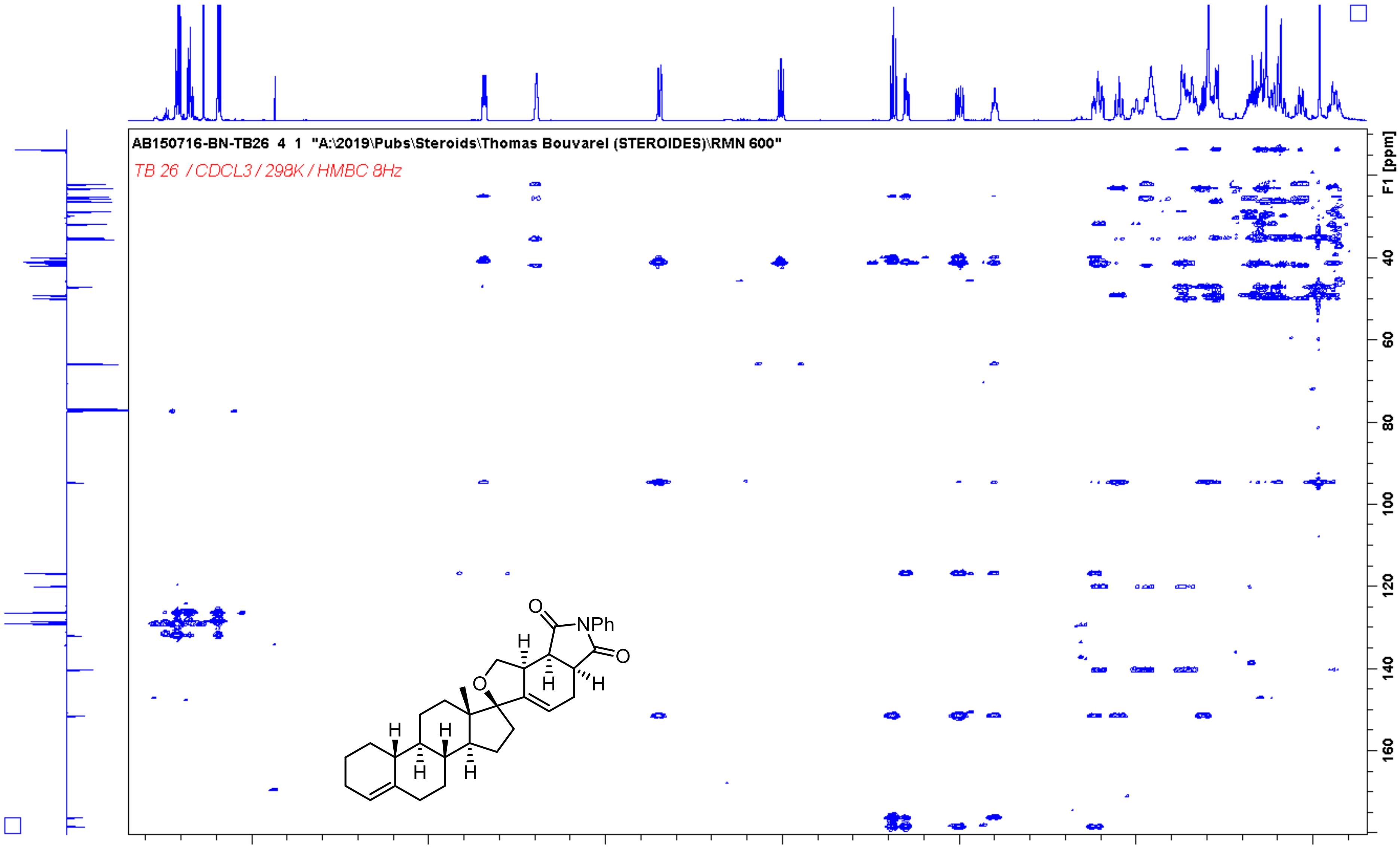

(3'*S*,5a'*S*,8*R*,8a'*R*,8b'*S*,9*S*,10*R*,13*S*,14*S*)-13-Methyl-7'-phenyl-1,1',2,3,5',5a',6,7,8,8b',9,10,11,12,13,14,15,16-octadecahydrospiro[cyclopenta[*a*]phenanthrene-17,3'-furo[3,4-*e*]isoindole]-6',8'-(7'*H*,8a'*H*)dione (17a): HSQC NMR (600 MHz, $CDCl_3$)

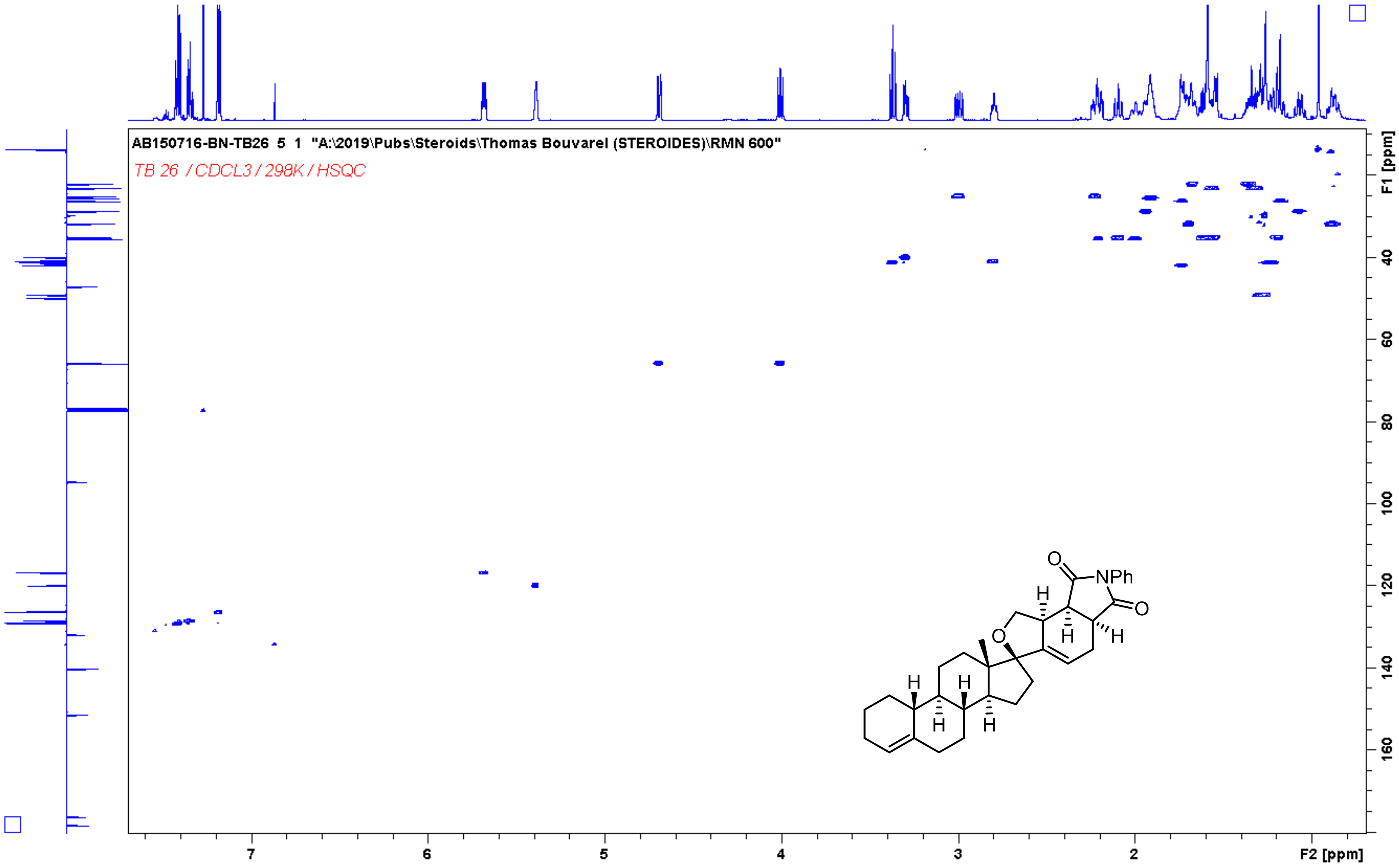

(3'*S*,5a'*S*,8*R*,8a'*R*,8b'*S*,9*S*,10*R*,13*S*,14*S*)-13-Methyl-7'-phenyl-1,1',2,3,5',5a',6,7,8,8b',9,10,11,12,13,14,15,16-octadecahydrospiro[cyclopenta[*a*]phenanthrene-17,3'-furo[3,4-*e*]isoindole]-6',8'(7'H,8a'*H*)dione (17a): NOESY NMR (600 MHz, CDCl₃)

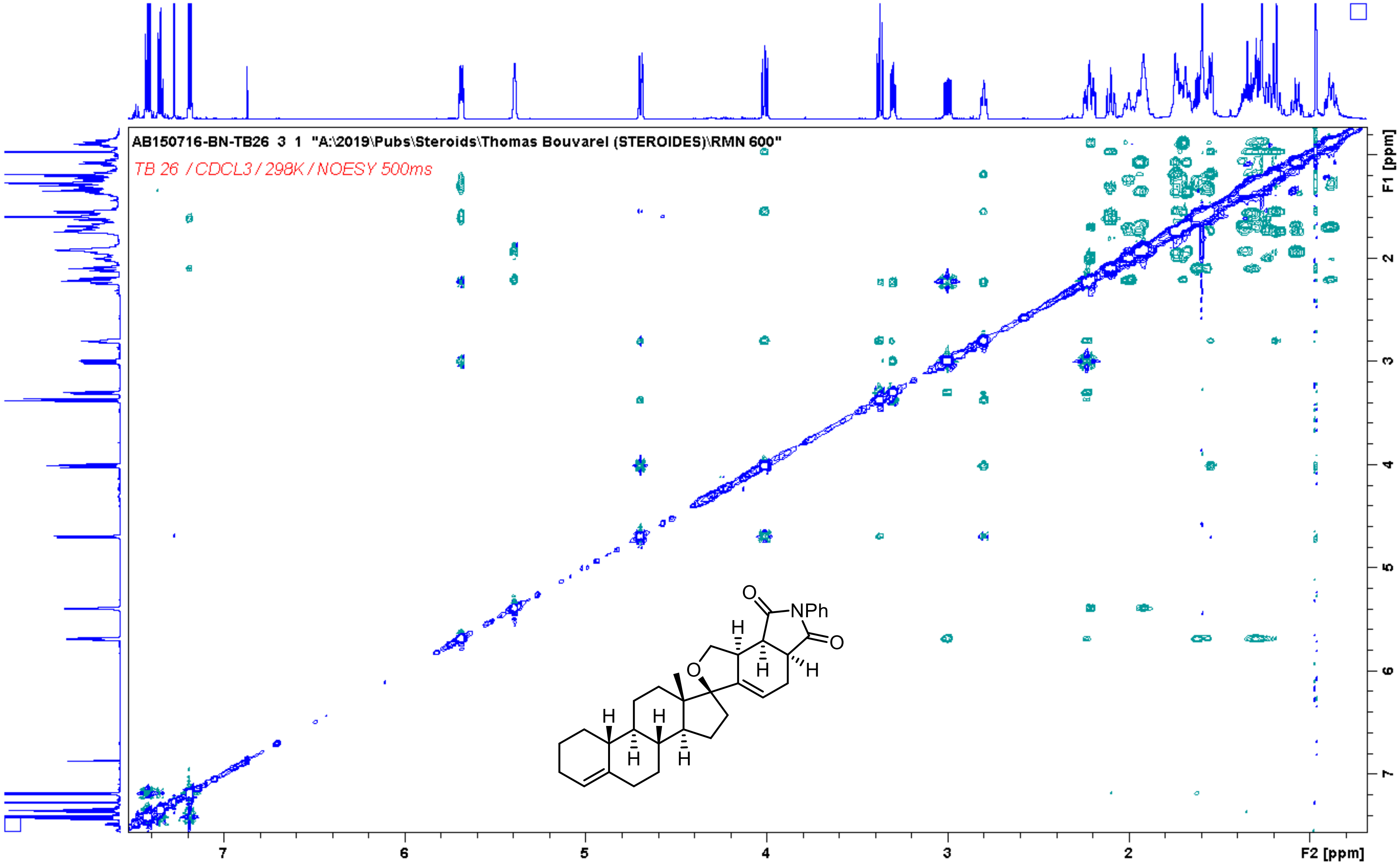

(3a'*S*,6'*S*,8*R*,9*S*,10*R*,10a'*S*,10b'*R*,13*S*,14*S*)-13-Methyl-2'-phenyl-1,2,3,3a',4',6,7,8,8',9,9',10,10',10a',11,12,13,14,15,16-icosahydrospiro[cyclopenta[*a*]phenanthrene-17,6'-oxepino[4,3-*e*]isoindole]-1',3'-(2'*H*,10b'*H*)dione (17b): ^{1}H NMR (600 MHz, CDCl$_3$)

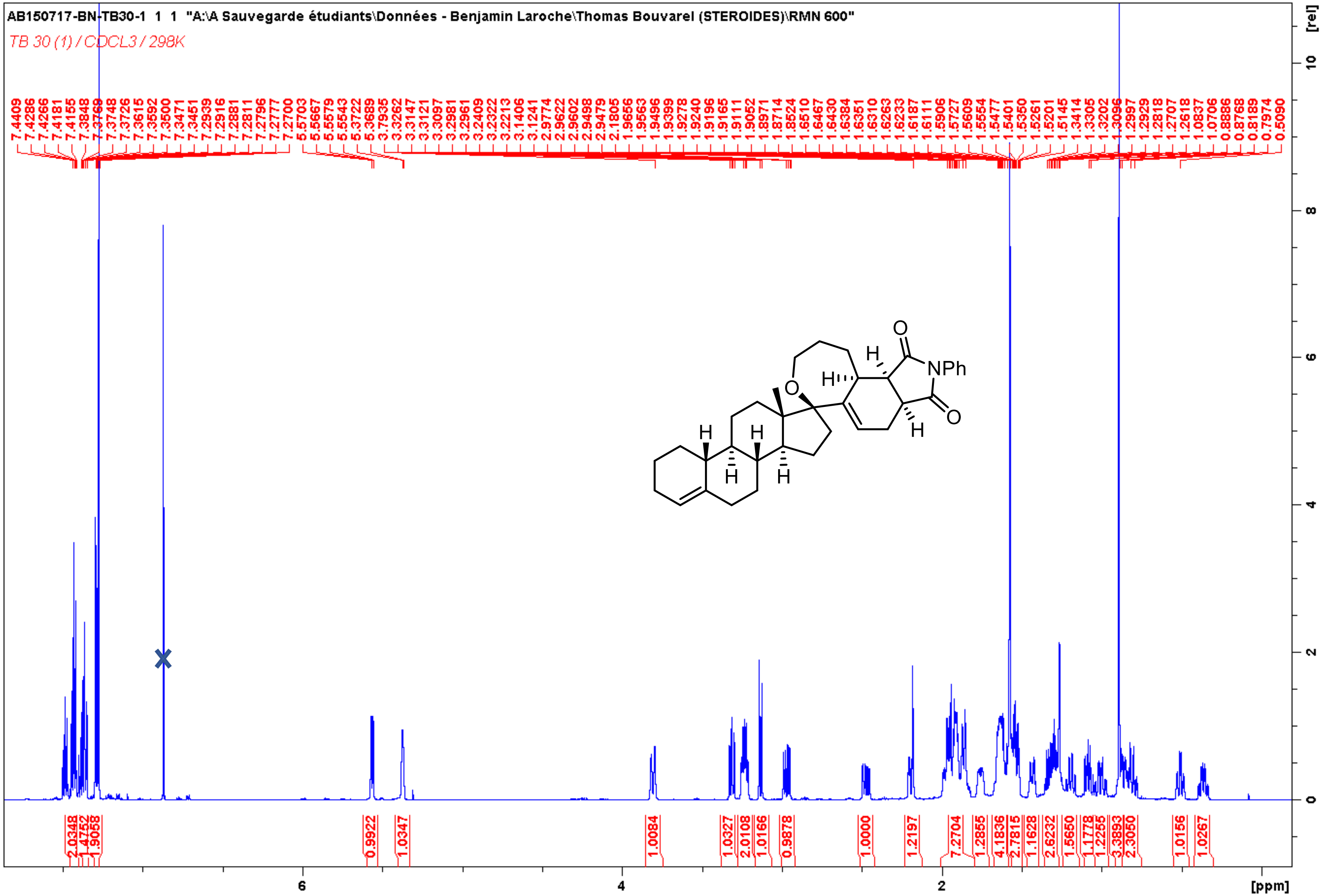

(3a'*S*,6'*S*,8*R*,9*S*,10*R*,10a'*S*,10b'*R*,13*S*,14*S*)-13-Methyl-2'-phenyl-1,2,3,3a',4',6,7,8,8',9,9',10,10',10a',11,12,13,14,15,16-icosahydrospiro[cyclopenta[*a*]phenanthrene-17,6'-oxepino[4,3-*e*]isoindole]-1',3'-(2'*H*,10b'*H*)dione (17b): ^{13}C NMR (150 MHz, $CDCl_3$)

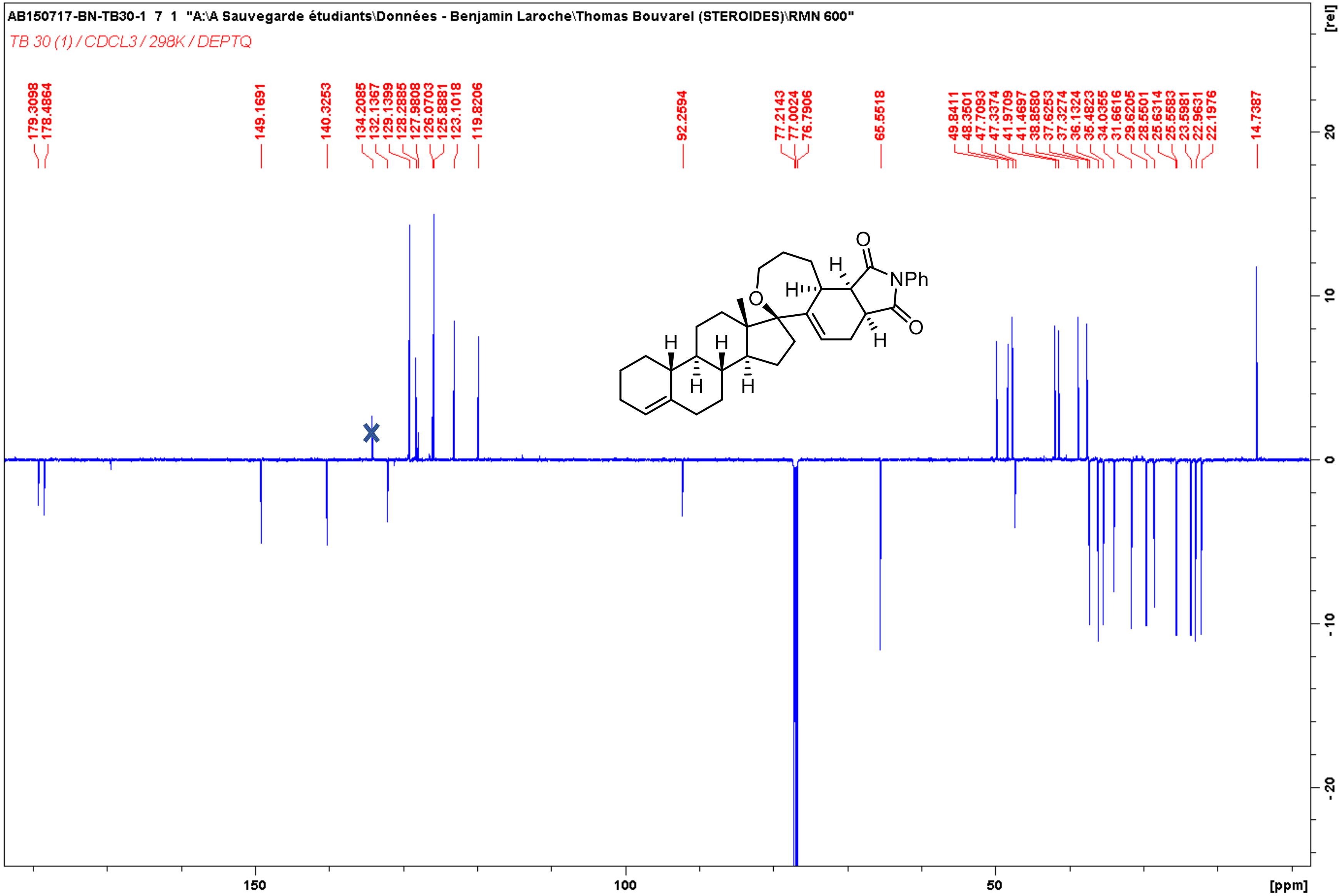

(3a'*S*,6'*S*,8*R*,9*S*,10*R*,10a'*S*,10b'*R*,13*S*,14*S*)-13-Methyl-2'-phenyl-1,2,3,3a',4',6,7,8,8',9,9',10,10',10a',11,12,13,14,15,16-icosahydrospiro[cyclopenta[*a*]phenanthrene-17,6'-oxepino[4,3-*e*]isoindole]-1',3'-(2'*H*,10b'*H*)dione (17b): COSY NMR (600 MHz, $CDCl_3$)

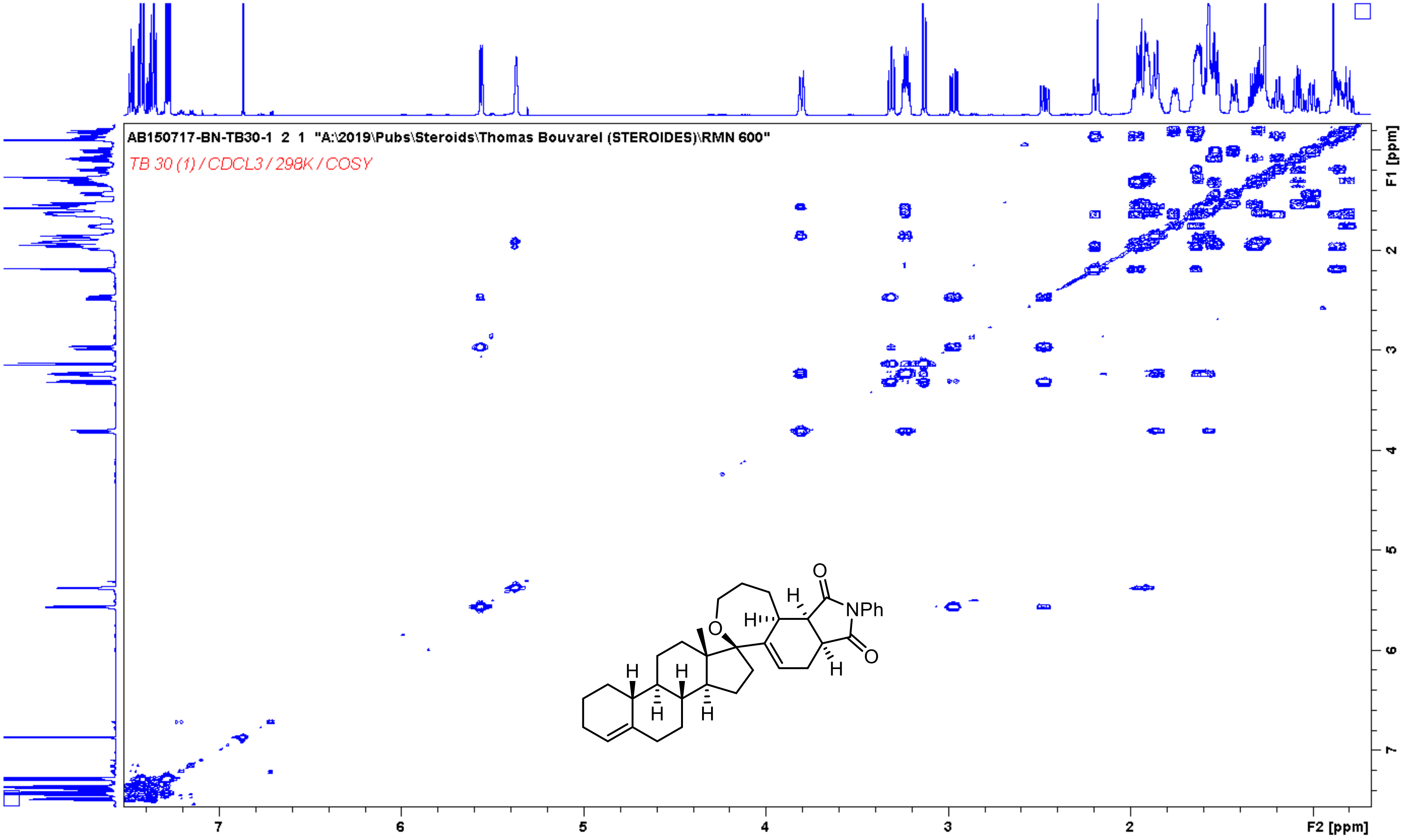

(3a'S,6'S,8R,9S,10R,10a'S,10b'R,13S,14S)-13-Methyl-2'-phenyl-1,2,3,3a',4',6,7,8,8',9,9',10,10',10a',11,12,13,14,15,16-icosahydrospiro[cyclopenta[*a*]phenanthrene-17,6'-oxepino[4,3-*e*]isoindole]-1',3'-(2'H,10b'H)dione (17b): HMBC NMR (600 MHz, $CDCl_3$)

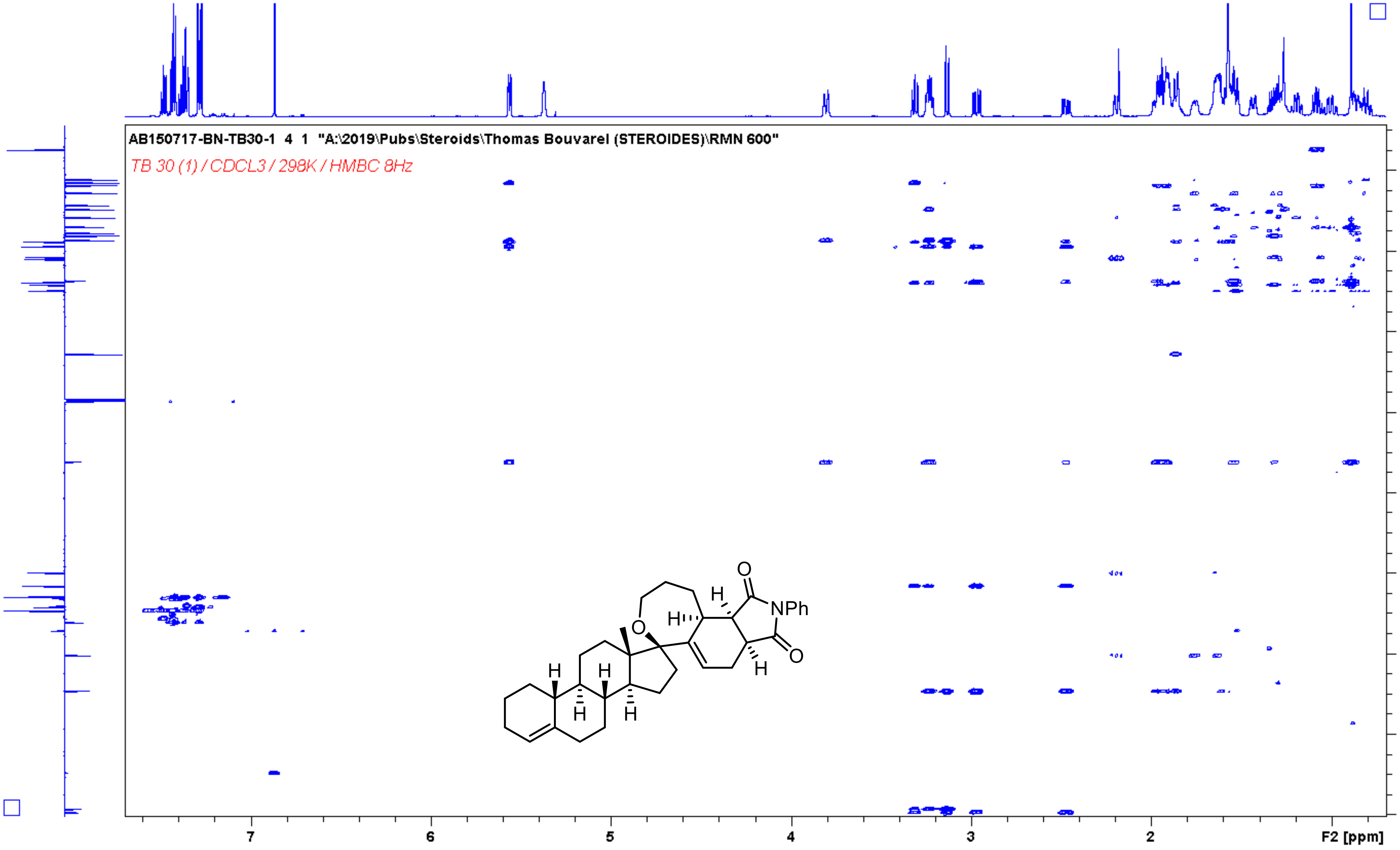

(3a'*S*,6'*S*,8*R*,9*S*,10*R*,10a'*S*,10b'*R*,13*S*,14*S*)-13-Methyl-2'-phenyl-1,2,3,3a',4',6,7,8,8',9,9',10,10',10a',11,12,13,14,15,16-icosahydrospiro[cyclopenta[*a*]phenanthrene-17,6'-oxepino[4,3-*e*]isoindole]-1',3'-(2'*H*,10b'*H*)dione (17b): HSQC NMR (600 MHz, $CDCl_3$)

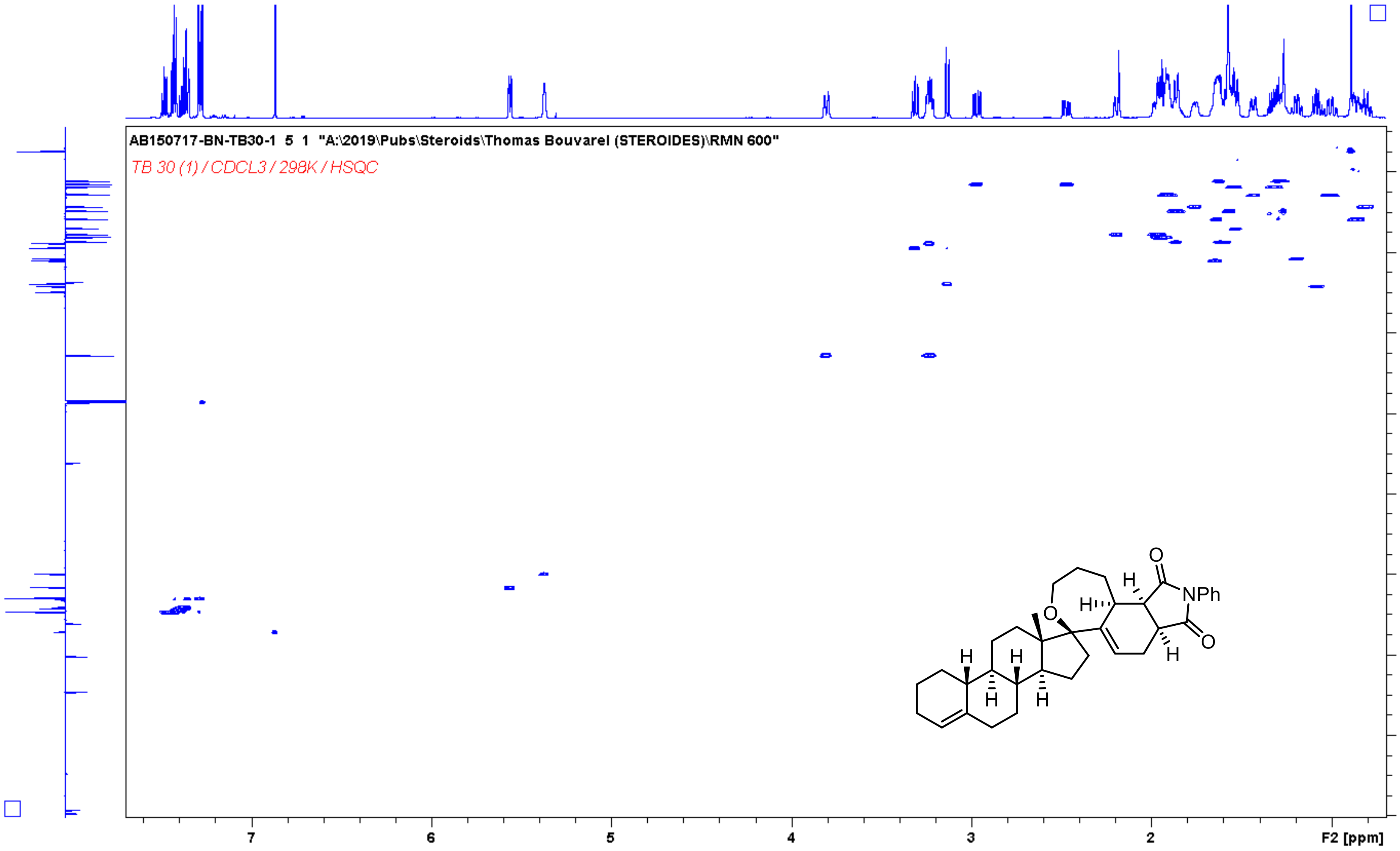

(3a'*S*,6'*S*,8*R*,9*S*,10*R*,10a'*S*,10b'*R*,13*S*,14*S*)-13-Methyl-2'-phenyl-1,2,3,3a',4',6,7,8,8',9,9',10,10',10a',11,12,13,14,15,16-icosahydrospiro[cyclopenta[*a*]phenanthrene-17,6'-oxepino[4,3-*e*]isoindole]-1',3'(2'*H*,10b'*H*)-dione (17b): NOESY NMR (600 MHz, $CDCl_3$)

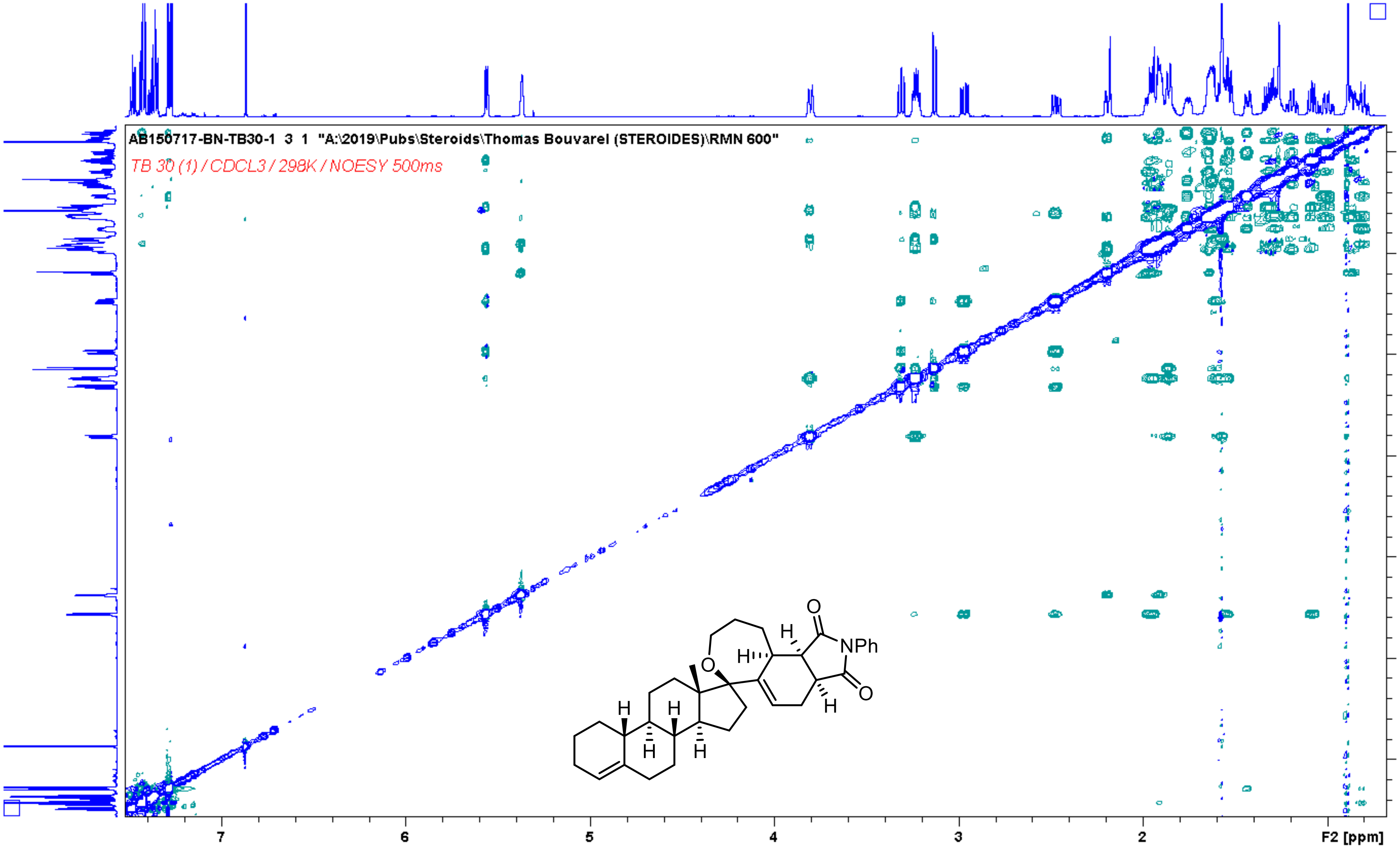

Supplement: File 1 — General experimental details, compound descriptions, 1H and 13C NMR spectra. [file Beilstein_J_Org_Chem-16-880-s001.pdf]
